# Structural similarity networks reveal brain vulnerability in dementia

**DOI:** 10.1002/alz.70973

**Published:** 2025-12-26

**Authors:** Marcella Montagnese, Amir Ebneabbasi, Natalia García‐San‐Martín, Clara Pecci‐Terroba, Rafael Romero‐García, Sarah E. Morgan, James H. Cole, Jakob Seidlitz, Timothy Rittman, Richard A. I. Bethlehem

**Affiliations:** ^1^ Department of Psychology University of Cambridge Cambridge UK; ^2^ Department of Clinical Neurosciences University of Cambridge Cambridge UK; ^3^ Department of Medical Physiology and Biophysics University of Seville Sevilla Spain; ^4^ Department of Psychiatry University of Cambridge Cambridge UK; ^5^ Instituto de Biomedicina de Sevilla (IBiS) HUVR/CSIC/University of Seville / CIBERSAM ISCIII Sevilla Spain; ^6^ School of Biomedical Engineering and Imaging Sciences King's College London London UK; ^7^ Department of Computer Science and Technology University of Cambridge Cambridge UK; ^8^ Department of Computer Science Hawkes Institute University College London London UK; ^9^ Dementia Research Centre UCL Queen Square Institute of Neurology London UK; ^10^ Lifespan Brain Institute The Children's Hospital of Philadelphia and Penn Medicine Philadelphia Pennsylvania USA; ^11^ Department of Psychiatry University of Pennsylvania Philadelphia Pennsylvania USA; ^12^ Department of Child and Adolescent Psychiatry and Behavioral Science The Children's Hospital of Philadelphia Philadelphia Pennsylvania USA; ^13^ Institute for Translational Medicine and Therapeutics University of Pennsylvania Philadelphia Pennsylvania USA; ^14^ Autism Research Centre Department of Psychiatry University of Cambridge Cambridge UK; ^15^ Brain Mapping Unit Department of Psychiatry University of Cambridge Cambridge UK

**Keywords:** Alzheimer's disease, brain networks, Morphometric Inverse Divergence (MIND) networks, morphometric similarity, neurodegeneration, neuropathology, normative modeling, personalized medicine, structural magnetic resonance imaging

## Abstract

**INTRODUCTION:**

Alzheimer's disease (AD) is characterized by inter‐individual heterogeneity in brain degeneration, limiting diagnostic and prognostic precision. We present a novel framework integrating Morphometric Inverse Divergence (MIND) networks with hierarchical Bayesian large‐scale population modeling to identify individual‐level neuroanatomical deviations.

**METHODS:**

MIND networks quantify similarity between brain regions using multivariate magnetic resonance imaging (MRI) features. A normative model of regional MIND values trained on UK Biobank (*N* = 35,133) was applied to the National Alzheimer's Coordinating Center cohort (*N* = 3,567). We examined brain deviations across clinical stages, apolipoprotein E (APOE) genotypes, mortality risk, and neuropathological burden.

**RESULTS:**

Negative deviations (reduced MIND) stratified disease stages (*p* < 0.01) and were concentrated in specific functional networks in AD. Greater negative deviations characterized APOE ε4 homozygotes and correlated with *post mortem* neuropathological severity (*p* = 0.032). Spatially, deviation patterns were associated with maps of neurotransmitter receptor density.

**DISCUSSION:**

This population neuroimaging modeling enables individualized brain mapping with direct utility for diagnosis, prognosis, and understanding of biological mechanisms.

**Highlights:**

- MIND networks were systematically integrated with normative modeling in AD.
- Negative deviations stratify clinical stages and correlate with neuropathology.
- Negative deviation count distinguishes APOE genotypes, highest in ε4 homozygotes.
- Deviations align with neurotransmitter maps.
- Individual brain maps enable precision medicine approaches in dementia.

## 1 BACKGROUND

Alzheimer's disease (AD) represents a complex neurodegenerative syndrome characterized by heterogeneity in clinical presentation, progression, and underlying biological mechanisms.^1^, ^2^ Despite progress, prevailing neuroimaging approaches continue to rely on group‐level comparisons – typically contrasting AD patients with cognitively healthy controls – potentially obscuring meaningful individual differences and limiting precision in diagnosis, prognosis, and treatment.^3^ As personalized medicine gains traction across neurology and psychiatry, there is a pressing need for imaging frameworks that can provide reliable, individual‐level characterizations of neurodegeneration.

Most neuroimaging studies rely on univariate approaches, examining features like cortical thickness (CT) or volume one at a time. While informative, such regionally specific approaches overlook the integrated, multivariate nature of cortical organization.^4^ The brain operates as an interconnected system and growing evidence demonstrates that structural relationships between regions provide insights beyond individual regional properties.^4^, ^5^, ^6^ This integrative approach may therefore offer crucial insights into dementia‐related brain patterns that are missed by traditional approaches. Recent advances enable the integration of multiple features into multivariate similarity networks. Among these, Morphometric Inverse Divergence (MIND)^7^ offers a biologically grounded method for constructing structural brain networks across cortical regions for each person, using more routinely collected structural T1‐weighted MRI, enhancing their translational potential. MIND quantifies how similar each brain region is to all others by estimating similarity between vertex‐level feature distributions across different quantitative morphometric measures like thickness, curvature, and volume. The resulting MIND degree provides a summary measure of how structurally integrated a region is across the cortex, capturing subtle relationships that single‐feature regional analyses may overlook. It also provides a biologically grounded representation of brain architecture that is more closely aligned with cortical cytoarchitectonic patterns and has stronger correspondence to axonal tract‐tracing data than earlier approaches.^7^ By estimating similarity between regions from their multivariate structural features, MIND captures individual‐specific, network‐level information from a single scan, providing a robust basis for detecting personalized deviations and being therefore best suited for our modeling framework.

**RESEARCH IN CONTEXT**

1. **Systematic review**. The literature was reviewed using traditional databases (e.g., PubMed). Prior studies have applied normative modeling to single brain features in neurodegeneration, but systematic assessments of multivariate structural approaches in neurodegeneration is limited. Furthermore, neuroimaging studies mostly rely on group‐average comparisons that obscure individual differences, limiting precision‐medicine approaches.
2. **Interpretation**. We present a systematic integration of MIND networks with normative modeling applied to AD. Our approach revealed that multivariate structural similarity metrics capture disease‐relevant patterns, at the network and regional levels. Negative deviations stratify clinical stages, differentiate apolipoprotein E (APOE) genotype and correlate with neuropathological severity, establishing their biological validity for personalized neuroanatomical characterization.
3. **Future directions**. Future research should focus on longitudinal dynamics of structural similarity breakdown and validation across diverse cohorts. Mechanistic studies linking structural similarity disruption to specific pathological processes and development of automated clinical pipelines represent critical next steps.

Interpreting these complex networks at the individual level requires a statistical framework for distinguishing pathological change from normal variation and healthy ageing. Neuroanatomical population modeling provides such a framework by quantifying how much an individual deviates from expected patterns based on a healthy reference population.^8^, ^9^, ^10^ Rather than comparing patients and controls at the group level, population modeling quantifies how each individual's brain deviates from expected patterns for their demographic profile, with most advanced implementations accounting for confounding factors such as age, sex, and site/scanner effects. This approach creates personalized benchmarks that highlight complex brain patterns unique to each patient, while univariate approaches provide more focused deviations in specific measures. This method has successfully provided individual‐level insights into neurodevelopmental and psychiatric disorders.^11^, ^12^, ^13^, ^14^, ^15^, ^16^, ^17^ While population modeling is well established in these fields, its application to neurodegenerative disease is more recent but rapidly expanding.^18^ Important recent normative work in dementia^19^, ^20^ has successfully applied this approach to CT and regional volume, demonstrating associations with cognitive decline and diagnostic conversion over time.^19^, ^20^, ^21^, ^22^ This work has provided useful insights into disease progression and heterogeneity cross‐sectionally and longitudinally^19^, ^21^ focusing on univariate models. Multivariate, network‐based approaches remain largely unexplored in large‐scale clinical datasets.

Building on this foundation, the National Alzheimer's Coordinating Center (NACC) database^23^ offers a vital opportunity to advance a hybrid normative modeling and multivariate morphometric approach in a clinical context – providing clinical, imaging, genetic, and neuropathological data for robust analyses of brain deviations.

Overall, we present a comprehensive clinical validation of MIND‐based normative modeling in AD and mild cognitive impairment (MCI). While recent work has explored MIND trajectories across the lifespan with alternative normative approaches,^18^ our study provides in‐depth clinical assessment specifically in dementia, leveraging the large, clinically heterogeneous NACC cohort. We integrate MIND with hierarchical Bayesian normative modeling to estimate individualized brain deviation maps and examine how these deviations relate to clinical diagnostic status, genetic risk (with particular focus on APOE ε4 effects), *post mortem* neuropathological burden, and clinical outcomes.

## 2 METHODS

### 2.1 Participants

Data for this study were derived from two datasets: (1) a reference (training) dataset including individuals from the UK Biobank employed for normative modeling and (2) a clinical dataset that included people with AD, MCI, and age‐matched cognitively healthy controls. The reference dataset from the UK Biobank^24^ included *N* = 35,133 participants between 44 and 81 years old (mean age 63.56 ± 7.55) with 53% females and 46.9% males. This dataset has been extensively described in prior work^24^, ^25^ and was used to train the initial normative model. Further details about the demographics and model implementation for this dataset can be found in Supporting Information and Figures S1–S6. The clinical test dataset was obtained from the NACC database,^23^ a publicly accessible multisite cohort (involving multiple ADRCs) that includes a wide range of clinical, demographic, neuropsychological, and neuropathological information. As presented in Table 1, the final samples used for this study included cognitively healthy controls y controls (NC, total *N* = 2,018), patients diagnosed with AD (*N* = 618), and individuals with MCI (total *N* = 931). All clinical diagnostic labels had already been provided for each patient in the NACC database. The MCI group was further subdivided into two categories based on cognitive trajectory: (1) MCI stable (*N *= 632) and (2) MCI progressive (*N *= 299), for those who showed cognitive decline, as measured as changes over 5 years from baseline scanning in the Clinical Dementia Rating (CDR) Dementia Staging Instrument. Data for this study were obtained from the Uniform Data Set (UDS) visits conducted between September 2005 and December 2022. Comprehensive descriptions of neuropsychological assessments, neuroimaging data, and neuropathological measures are provided in the following subsections.

**TABLE 1:** Final samples for NACC dataset showing demographics and clinical characteristics by diagnostic group.

Diagnostic group	Age (mean ± SD)	Gender (%)	Education (mean ± SD)	MMSE (mean ± SD)	CDR (mean ± SD)	MoCA (mean ± SD)
**AD (*n* = 618)**	73.32 ± 9.63	F: 330 (53.40%), M: 288 (46.60%)	14.58 ± 3.55	20.45 ± 5.86^a^	0.99 ± 0.60	14.29 ± 6.14^a^
**MCI stable (*n* = 632)**	71.55 ± 9.09	F: 350 (55.38%), M: 282 (44.62%)	15.10 ± 3.53^a^	27.06 ± 2.54^a^	0.44 ± 0.19	23.09 ± 3.34^a^
**MCI progressive (*n* = 299)**	74.56 ± 7.42	F: 153 (51.17%), M: 146 (48.83%)	15.34 ± 3.32	26.02 ± 2.67^a^	0.51 ± 0.13	20.67 ± 3.44^a^
**NC_test_ (*N* = 394)**	66.11 ± 12.90	F: 279 (70.81%), M: 115 (29.19%)	16.04 ± 2.66^a^	28.96 ± 1.27^a^	0.07 ± 0.17	26.61 ± 2.50^a^
**NC_Train_ (*N* = 1,624)**	66.19 ± 10.57	F: 1121 (69.03%), M: 503 (30.97%)	15.96 ± 2.87	28.97 ± 1.33^a^	0.07 ± 0.17	26.72 ± 2.52^a^

Abbreviations: AD; Alzheimer's disease; CDR; Clinical Dementia Rating; MCI; mild cognitive impairment; MMSE: Mini‐Mental State Examination; MoCA; Montreal Cognitive Assessment; SD; squared deviation.

^a^ Indicates missing values.

### 2.2 NACC participant characteristics and cognitive measures

Key demographic and clinical variables were obtained from the NACC UDS. Years of education reflect the total years of formal schooling as recorded in the UDS. APOE genotype was derived from available genetic data and reflects allele frequencies for ε2, ε3, and ε4, as provided by NACC. Cognitive function was assessed using two tests: the Mini‐Mental State Examination^26^ (MMSE, range 0 to 30) and the Montreal Cognitive Assessment^27^ (MoCA, range 0 to 30). Both are widely used screening tools for global cognitive impairment, with lower scores indicating greater impairment. Global disease severity was evaluated using the CDR Dementia Staging Instrument,^28^ which assesses impairment across six domains (memory, orientation, judgment/problem solving, community affairs, home/hobbies, and personal care) to generate a global score ranging from 0 (no impairment) to 3 (severe dementia). All values represent those recorded at the participants’ initial visit and are summarized in Table 1 below.

### 2.3 Neuropathology *post mortem* measures in NACC

For a subset of participants (*N* = 240) with *post mortem* data, neuropathological assessment had been performed according to National Institute on Aging–Alzheimer's Association (NIA‐AA) guidelines^29^, ^30^ for the assessment of Alzheimer's disease neuropathologic change (ADNC). The primary neuropathological measure was the ABC score (NACC variable: “NPADNC”), a composite ordinal index provided directly by NACC and ranging from 0 (no AD pathology) to 3 (high AD pathology) that integrates amyloid beta (Aβ) deposition, tau neurofibrillary degeneration, and neuritic plaque burden.^31^ Specifically, the ABC score includes these three complementary pathological components: (“A” component) Thal phase^32^ for Aβ plaque distribution (0 to 5): Phase 0 indicates no amyloid; phases 1 and 2 represent initial neocortical involvement; phase 3 adds allocortical regions; phase 4 includes subcortical structures; and phase 5 shows widespread cerebral and cerebellar distribution. (“B” component) Braak stage^33^, ^34^ for neurofibrillary tau pathology (0 to VI): Stages I and II involve trans‐entorhinal regions; III and IV extend to limbic structures; and V and VI indicate extensive neocortical involvement. (“C” component) Consortium to Establish a Registry for Alzheimer's Disease (CERAD) score^35^ for neuritic plaque density (0 to 3): 0 = none, 1 = sparse, 2 = moderate, 3 = frequent neocortical neuritic plaques.

### 2.4 MRI acquisition and preprocessing

Detailed MRI protocols, including T1‐weighted sequences, are available online for both the clinical dataset^23^ and the UK Biobank reference dataset.^25^, ^36^ For the UK Biobank, T1‐weighted images were acquired on Siemens Skyra 3T scanners using a 3D MPRAGE sequence. For NACC, T1‐weighted images were acquired using various scanner manufacturers (Siemens, GE, Philips) at both 1.5T and 3T field strengths across 13 ADRCs included in the current analyses. For the reference cohort FreeSurfer version 6.0.1 was used for preprocessing, while FreeSurfer version 7.2 was used for NACC. For quality controls, following the methodology outlined in previous studies.^8^ We excluded scans with an Euler Index (which quantifies topological defects/holes in the surface reconstruction of both hemispheres) of over *N* = 2 median absolute deviations (MAD). Additionally, to address potential confounding by head size, we removed outliers in estimated total intracranial volume (eTIV), defined as values outside the interquartile range for each diagnostic and sex group. Detailed exclusion numbers and proportions are provided in the Supporting Information (including Figures S1– S6). The final sample composition is detailed in Table 1 and our analyses included data from 13 ADRCs in the adaptation and test set.

### 2.5 MIND network construction and degrees estimation

MIND networks were computed to characterize individualized structural similarity across the cortex and have been described in detail in previous work.^7^ Briefly, this approach estimates pairwise anatomical similarity between brain regions by quantifying divergence in multiple morphometric features derived from T1‐weighted MRI. In our study, following the same methodology of Sebenius and colleagues,^7^ we extracted five features for each vertex on the cortical surface from a participant's T1‐weighted MRI using FreeSurfer's *recon‐all* pipeline, namely: CT, mean curvature, sulcal depth, surface area, and gray matter (GM) volume. These features were chosen in accordance with prior studies^7^, ^37^; sensitivity analyses confirmed that the selected features each made a distinct contribution to the final MIND metric (Figure S7).

In line with Sebenius et al.,^7^ before computing similarity, each feature was *Z*‐scored across all cortical vertices to ensure all measures were on a common scale. The vertex‐level data were then aggregated within each of the 360 cortical regions of the Human Connectome Project Multimodal Glasser parcellation (HCP‐MMP)^38^ to form a regional multivariate distribution. To quantify the similarity between each pair of regions (*a,b*), we first estimated the symmetric Kullback‐Leibler (KL) divergence between their distributions using a *k*‐nearest neighbor algorithm. This divergence value, KL(a,b), was then transformed to produce the final MIND similarity score, bounded between 0 and 1, using the following formula: $MIND\left(a,b\right)=\frac{1}{\left(1+KL(a,b)\right)}$. Higher values in the resulting 360 × 360 symmetric similarity matrix indicate greater morphometric similarity. From this matrix the weighted degree for each node was computed by averaging its similarity values with all other regions, producing a vector of 360 regional similarity scores per subject (one score per cortical region in our parcellation).

We focused on the node‐level weighted degree for two main reasons. First, this aligns with Sebenius et al.,^7^ where weighted degree was the primary nodal measure reported. Second, in large‐scale normative modeling (>50,000 edges per participant), modeling the full edge set would be computationally prohibitive and limit the method's clinical scalability. Weighted degree offers a robust, interpretable, and computationally tractable measure for large datasets. Importantly, here weighted MIND degree reflects the average similarity (in its multivariate morphometric profile) between a region and all others,^7^ which can be thought of as a proxy for how morphometrically integrated it is. Detailed quality control analyses of feature contributions and network construction characteristics are provided in Figure S7.

### 2.6 Normative modeling of MIND Networks

We first trained a normative model on MIND weighted degree (derived as outlined in the previous section) from T1‐weighted MRI data of *N *= 35,133 UK Biobank participants. We employed a hierarchical Bayesian regression (HBR) model^39^ due to its robustness in handling multisite datasets,^40^ allowing for the integration of site‐specific variability while accounting for confounding factors such as scanner differences and sampling biases. This method has proven particularly effective and was used in previous studies.^19^, ^41^ The model was implemented using the openly available Predictive Clinical Neuroscience (PCN) toolkit^42^ (https://github.com/predictive‐clinical‐neuroscience/braincharts) and employed a sinh–arcsinh (SHASHb) likelihood for enhanced modeling flexibility and to capture non‐Gaussian characteristics in the data distributions.^40^ Age and sex were included as fixed effects, while scanning site was modeled as a random effect. This approach appropriately models site differences as random offsets from a group mean while sharing information across sites. We implemented separate normative models for each of the 360 regions in our parcellation. Model performance evaluation including explained variance distributions across regions is shown in Supporting Information. All analyses were run on the Cambridge High‐Performance Cluster.

Following initial training on the UK Biobank dataset, we employed an adaptive transfer learning approach^43^ to calibrate the estimates to our clinical dataset from NACC. We used 80% of the NACC controls (NC_train_, *N *= 1,624) to recalibrate the model parameters, establishing a NACC‐specific normative baseline. The remaining 20% of controls (NC_test_, *N *= 394), along with all patients diagnosed with AD (*N *= 618), MCI stable (*N *= 632), and MCI progressive (*N *= 299) formed the test set for evaluating deviations from the normative model (total test set *N *= 1,943). All sites included in the test set were also represented in the adaptation dataset to ensure proper calibration of site‐specific parameters.

For each participant in the test set, regional *Z*‐scores were calculated after fitting a separate normative model for each region. These *Z*‐scores quantify the degree of deviation from typical structural patterns observed in healthy aging. We defined extreme deviations as regions with *Z*‐scores exceeding |1.96|, corresponding to a *p* value of < 0.05, following thresholds established in previous work.^41^ Regions where *Z*‐scores exceeded this threshold were considered as extreme deviations. Each participant's total deviation count was computed as the sum of brain regions with such extreme deviations, again to stay in line with previous work.^41^ Most analyses were performed separately on positive and negative deviations to better capture the direction‐specific nature of brain changes, as these deviations may reflect different underlying neurodegeneration processes. To account for the potential confounding effect of head size, we residualized regional *Z*‐scores against eTIV separately for each demographic group (by sex and diagnosis), ensuring that findings reflected disease‐related changes rather than anatomical variations due to head size (full details in Supporting Information). Quality control analyses confirmed model robustness, with no significant correlation between age and deviations (r = −0.026, *p* = 0.250). Further details on model implementation, parameters, and quality control can be found in Supporting Information (Figures S1–S7). All analyses were run in Python 3.12.4.

### 2.7 Comparative analyses with univariate metrics

We later conducted sensitivity analyses to see whether MIND's multivariate integration provided advantages over simpler univariate approaches. We repeated the complete normative modeling framework detailed in previous sections, but this time using CT and GM as alternative input features. This involved training separate normative models for each metric (2 metrics × 360 cortical regions = 720 additional models) on the same UK Biobank reference dataset (*N* = 35,133) and NACC adaptation set, following identical procedures to those described above for MIND. The same deviation quantification, statistical analyses (diagnostic group comparisons, APOE associations, neuropathology prediction), were applied to CT and GM deviation counts. Complete methodological details and results are provided in the Supporting Information, (including Figures S8 and S9 and Table S1).

### 2.8 Group‐level deviations and network‐specific targeting

To assess how MIND‐based deviations stratify clinical stages and target known brain systems, we first compared deviations across all diagnostic groups. For each participant, positive and negative deviation counts – defined as the total number of cortical regions with *Z*‐scores exceeding +1.96 or below −1.96, respectively – were calculated, corresponding to the top and bottom 2.5% of the normative distribution (two‐tailed *p* < 0.05). We used deviation counts as a summary metric of an individual's total burden, an approach in line with previous work,^19^ which is suitable for group‐level statistical comparisons and subsequent regression analyses. Separate one‐way ANOVAs were conducted to compare positive and negative total deviation counts across groups (AD, MCI progressive, MCI stable, and controls). Post hoc comparisons were performed using Tukey's Honestly Significant Difference (HSD) test to identify pairwise group differences.

To examine spatial patterns of deviations, we implemented a regional overlap analysis based on work by Segal et al.^16^ For each region and diagnostic group, we computed the proportion of individuals with positive and negative deviations. We then subtracted the corresponding overlap rate in the held‐out control group (nc_test_) from each clinical group to create deviation difference maps, highlighting regional increases or decreases in deviation prevalence. To assess statistical significance, we used permutation testing (*N* = 10,000), shuffling diagnostic labels to generate null distributions of regional overlap differences while preserving group sizes. Directional *p* values were computed for each region (both patient > control and control > patient), and false discovery rate (FDR) correction was applied.

We next tested whether deviations aggregated within known brain systems using a network‐wise overlap analysis, based once again on previously published work in psychiatric cohorts by Segal and colleagues.^16^ For each of the Yeo and Krienen seven functional networks,^44^ we computed the proportion of individuals in each group with at least one extreme deviation in the network (|Z| > 1.96). Normative benchmarks were computed from held‐out NC participants. Δ‐overlap maps were derived by subtracting this control overlap map from each clinical group's map.

To test whether extreme deviations were statistically over‐represented within specific functional systems, we implemented two complementary null models. First, we used group‐label permutation testing (*N *= 10,000), which preserved group size but randomly reassigned diagnostic labels. Second, to ensure observed network differences could not be explained by chance spatial structure or global deviation burden, we generated spatially constrained null maps using the Hungarian algorithm with the *neuromaps* toolbox,^45^ preserving the spatial structure of cortical maps while randomizing spatial location. Both nulls were used to generate empirical *p* values, with FDR correction applied across networks.

Finally, to complement this categorical network‐level approach with a continuous group‐level contrast, we performed permutation‐based significance testing of regional Cohen's *d* effect sizes across three pairwise diagnostic comparisons: MCI stable versus MCI progressive, MCI stable versus AD, and MCI progressive versus AD. For each comparison, we calculated observed Cohen's *d* values between groups for each region of interest (ROI)'s deviation scores, with effect directionality set such that the more severe diagnostic group was always subtracted from the less severe group to aid interpretability. Statistical significance was determined through *N *= 10,000 random permutations of diagnostic labels, generating empirical two‐tailed *p* values, with FDR correction applied to address multiple comparisons. These effect size maps quantified structural deviations across key stages of clinical progression within the Glasser atlas parcellation. While Cohen's *d* comparisons highlight robust group‐level effects, they assume within‐group homogeneity. To assess this assumption, we computed pairwise Hamming distances between individual binary deviation maps for negative and positive deviations separately.

### 2.9 Genetic stratification by APOE genotype

We next explored whether deviations reflected underlying genetic risk. In line with established APOE classifications used in recent work,^46^ participants were grouped into four APOE genotype categories: ε3 homozygotes (reference group), ε2 carriers (ε2/ε2, ε2/ε3), ε4 heterozygotes (ε3/ε4), and ε4 homozygotes (ε4/ε4). Individuals with ε2/ε4 genotypes (*N* = 32) were excluded due to opposing risk profiles, and those missing APOE data were removed.^46^ Final group sizes were: ε3 homozygotes (*N* = 556), ε4 heterozygotes (*N* = 517), ε4 homozygotes (*N* = 133), and ε2 carriers (*N* = 93).

We analyzed two deviation metrics derived from MIND normative modeling: (1) positive deviation count (number of regions with *z* > 1.96), and (2) negative deviation count (number of regions with z < −1.96). For each outcome, we fitted a series of nested ordinary least squares (OLS) regression models with APOE group as a categorical predictor (ε3 homozygotes as reference) and age and sex as covariates. To assess the presence of interactions, we systematically tested (1) a basic additive model, (2) models including APOE × age interactions, (3) models including APOE × sex interactions, and (4) a full model with all two‐way and three‐way interactions. Model selection was performed using Akaike information criterion (AIC) with likelihood ratio tests used to evaluate the significance of interaction terms against the basic model. Post hoc pairwise comparisons between APOE groups were conducted using Tukey's test.

### 2.10 Prognostic analysis: mortality

To evaluate whether brain deviations predicted mortality, we used Cox proportional hazards regression. Consistent with prior work of normative modeling in AD,^21^, ^47^ for this analysis we focused on negative deviations only, which more reliably reflect clinical progression. Survival time was defined as the interval (in years) between age at MRI acquisition and either age at death (for deceased individuals) or age at last follow‐up (for censored individuals). A total of *N* = 558 participants died during follow‐up (AD: 330, MCI progressive: 134, MCI stable: 94), while *N* = 991 participants (AD: 288, MCI progressive: 165, MCI stable: 538) were right‐censored at their last known follow‐up, determined from longitudinal NACC dataset records.

We used the previously defined negative deviation count (number of cortical regions with *Z*‐scores < −1.96) as a continuous predictor in the survival model. A Cox proportional hazards model was initially fit including age at MRI, sex, negative deviation count, and diagnostic group as covariates. Proportional hazards assumptions were assessed using scaled Schoenfeld residuals. While individual covariates (age, sex, negative deviation count) met the proportional hazards assumption (all *p* > 0.05), a violation was observed for the diagnostic group variable (*p* < 0.001). To address this violation, we employed two complementary approaches: (1) a stratified Cox model by diagnostic group that allowed different baseline hazard functions while estimating a common effect of deviation count across groups and (2) Cox models fitted separately for each diagnostic group to evaluate within‐group effects and assess potential heterogeneity in prognostic value across disease stages.

For visualization purposes, the total count of cortical regions with *Z*‐scores below −1.96 was binarized at the cohort median to create a high versus low deviation group. Kaplan–Meier survival curves were plotted using this binary classification, with survival differences evaluated using log‐rank tests. All survival analyses were conducted in R version 4.5.0.

### 2.11 Neurobiological decoding of structural deviations

To enhance mechanistic interpretations of structural deviations identified through normative modeling, we performed spatial co‐location analyses between deviation maps and a comprehensive set of cortical reference maps. Following established practice in the field and from previous directly relevant work,^16^, ^21^, ^47^ we used regional *Z*‐scores for this analysis, as they provide a continuous, region‐specific measure of deviation required for spatial correlation analyses. We bilaterally averaged regional deviation values by pairing left–right homologues in the HCP–MMP atlas and computing their mean, generating one bilateral value per region. This approach improves compatibility with heterogeneous reference datasets and reduces reliance on hemisphere‐specific patterns.

We examined spatial correspondence between MIND deviation patterns and neurotransmitter receptor density maps derived from positron emission tomography meta‐analyses (e.g., for serotonin 5‐HT2a, dopamine D1, GABAa, and nicotinic α4β2 receptors – originally compiled by Hansen et al.^48^). Neurotransmitters are especially relevant in the context of the MIND metric and AD because they not only shape large‐scale inter‐regional coupling^49^, ^50^ but are also highlighted in AD pathology (e.g., cholinergic and glutamatergic dysfunction^51^, ^52^, ^53^). Anchoring MIND deviations to these neurochemical gradients, therefore, provides a biologically meaningful link between structural network alterations and known mechanisms of AD vulnerability. Exploratory analyses with additional maps selected based on prior work^48^, ^54^, ^55^ and covering a wide range of cortical properties relevant to brain network organization, cortical hierarchy, and disease vulnerability patterns can be found in the Supporting Information. All these maps were retrieved from prior original work^48^, ^54^, ^55^, ^56^, ^57^, ^58^, ^59^ or from the *neuromaps* toolbox.^45^ To account for spatial autocorrelation when evaluating cortical map correlations, we applied spin permutation testing using the Hungarian rotation method. Cortical centroid coordinates were rotated on a spherical surface to generate *N* = 5000 spatially permuted versions of the parcellation. These permutations were then used to reorder the deviation maps, producing null distributions of correlation values for each biological reference map. Empirical spatial correlations were compared against these null distributions to derive spin‐corrected *p* values, which were used to assess statistical significance.

### 2.12 Relationship with *post mortem* pathology

To validate the biological relevance of MIND deviations, we tested whether deviation counts were associated with *post mortem* pathology. This exploratory analysis addressed the need for biological validation of structural similarity networks against histological benchmarks.^4^ For a subset of participants (*N *= 240) with autopsy‐confirmed pathology in NACC (see further details in Supporting Information), we therefore examined associations between total deviation count and a combined neuropathological score available in this cohort.^60^ Neuropathological analyses were performed in accordance with the NIA‐AA guidelines^29^, ^30^ for the assessment of ADNC. In line with previous work,^61^ the primary measure used for stratification was the ABC score (NACC variable: NPADNC), a composite ordinal index ranging from 0 = “No AD pathology” to 3 = “High AD pathology,” and that integrates three key pathological processes associated with AD (as described in more detail in Section 2.3): Thal phase (“A” component), Braak stage (“B” component), and CERAD rating of neuritic plaque density (“C” component).

To assess whether the number of deviations – positive and negative, respectively – was associated with the severity of neuropathological burden, we modeled the relationship between deviation count and ABC score using ordinal logistic regression. Age at MRI, sex, and time to death were included as covariates in all models. A basic model adjusted for demographic and clinical variables provided an initial assessment. An age interaction model introduced interaction terms between ABC score and age at MRI to investigate age‐dependent neuroimaging and cognitive performance effects. A third model, with time‐to‐death interaction, explored disease progression dynamics. Model comparison was based on the AIC. The main analysis focused on the composite ABC score, with exploratory analyses of the individual A, B, and C components conducted and reported in the Supporting Information. Participants with missing or invalid pathology scores (i.e., values of 8, 9, or −4 in the NACC dataset) were excluded from all relevant analyses. All regression models were implemented using the *statsmodels* Python library. Post hoc comparisons were performed using Tukey's test to evaluate pairwise differences between ABC levels while controlling for multiple comparisons. A two‐sided significance threshold of *p* < 0.05 was used throughout.

## 3 RESULTS

### 3.1 Group differences in positive and negative deviation counts

There were no significant group differences in positive deviation count (ANOVA: F[3,1939] = 0.59, *p* = 0.62). In contrast, negative deviation count differed significantly across groups (ANOVA: F[3,1939] = 24.39, *p* < 0.001). Post hoc Tukey tests revealed that individuals with AD had significantly higher negative deviation counts compared to all other groups (AD vs MCI progressive: *p* = 0.001; AD vs MCI stable and controls (NC): *p* < 0.001). MCI progressive individuals also showed more negative deviations than controls (*p* = 0.049), but not significantly more than MCI stable (see Table S2 for full ANOVA and post hoc results). Additional sensitivity analyses compared negative‐deviation counts derived from MIND, CT, and GM. One‐way ANOVA showed significant group effects across all metrics (all *p* < 0.001), though with different effect sizes: CT showed the largest overall effect (*η* ^2^ = 0.067), followed by GM (*η* ^2^ = 0.056) and MIND (*η* ^2^ = 0.036). However, post hoc Tukey HSD tests revealed that only MIND detected significant separation between MCI progressive and controls (*Δ* = 2.28, 95% CI [0.01, 4.56], adjusted *p* = 0.049), while all three metrics robustly separated AD from other groups. This indicates MIND provides added sensitivity for early‐stage clinical stratification. Full details and results are in the Supporting Information.

### 3.2 Spatial overlap and significance of individual deviations

**FIGURE 1:**
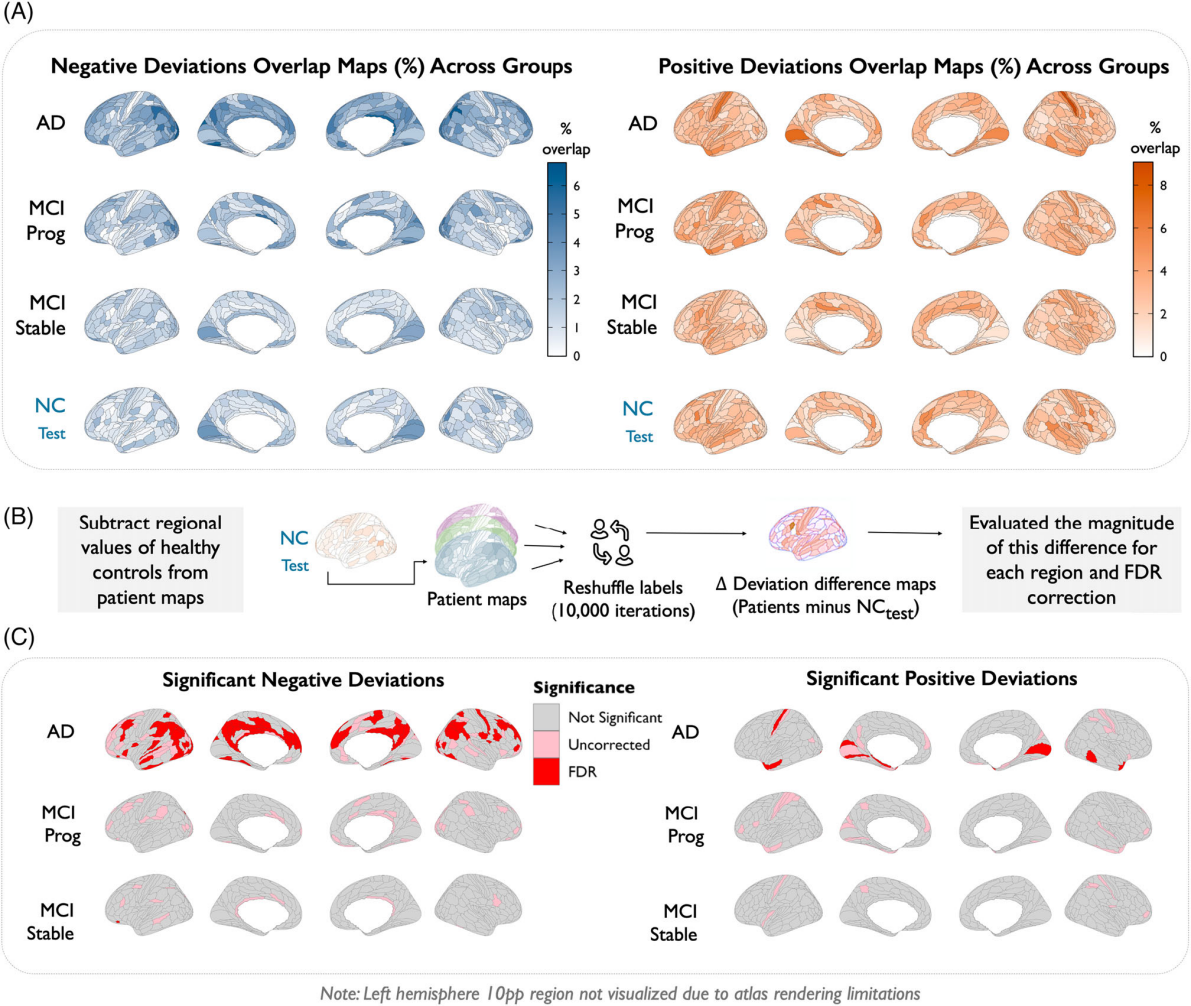
Group‐level spatial overlap and statistical significance of cortical deviations. (A) Left in blue: Regional overlap maps show percentage of individuals within each diagnostic group exhibiting extreme negative deviations (*Z* < −1.96). Right in orange: Regional overlap maps show percentage of individuals within each diagnostic group exhibiting extreme positive deviations (*Z* > 1.96). (B) Schematic diagram explaining how we calculated the deviation difference maps (C) Left: Cortical regions with significantly higher negative deviation overlap relative to controls, based on permutation testing (*N* = 10,000). Right: cortical regions with significantly higher positive deviation overlap relative to controls, based on permutation testing (*N* = 10,000). In both panels, red indicates regions significant after FDR correction, and pink shows uncorrected significance (*p* < 0.05). NC,  normal controls (test set); MCI, mild cognitive impairment.

To assess whether cortical deviations aggregated non‐randomly across diagnostic groups, we computed the proportion of individuals with extreme positive or negative deviations for each region, generating group‐level overlap maps (Figure 1A). While deviation overlap was generally low across the cortex, AD exhibited notably higher rates of overlap, particularly for negative deviations in medial temporal, lateral temporal, inferior parietal, and posterior cingulate cortices. Positive deviations were more spatially restricted and mostly in visual and ventral temporal regions.

To determine whether observed patterns exceeded those expected by chance, we computed difference maps by subtracting control group overlap from each clinical group and used permutation testing to assess statistical significance (schematics shown in Figure 1B). Extensive clusters of FDR‐significant negative deviations were observed in AD, with circumscribed effects in MCI progressive and only very few significant regions observed in MCI stable, although these did not survive FDR correction (shown in lighter pink in left panel of Figure 1C). Positive deviation analysis showed fewer significant regions across all groups, with only some effects evident in AD remaining statistically significant after multiple comparison correction (right panel of Figure 1C).

### 3.3 Network‐level enrichment of cortical deviations

**FIGURE 2:**
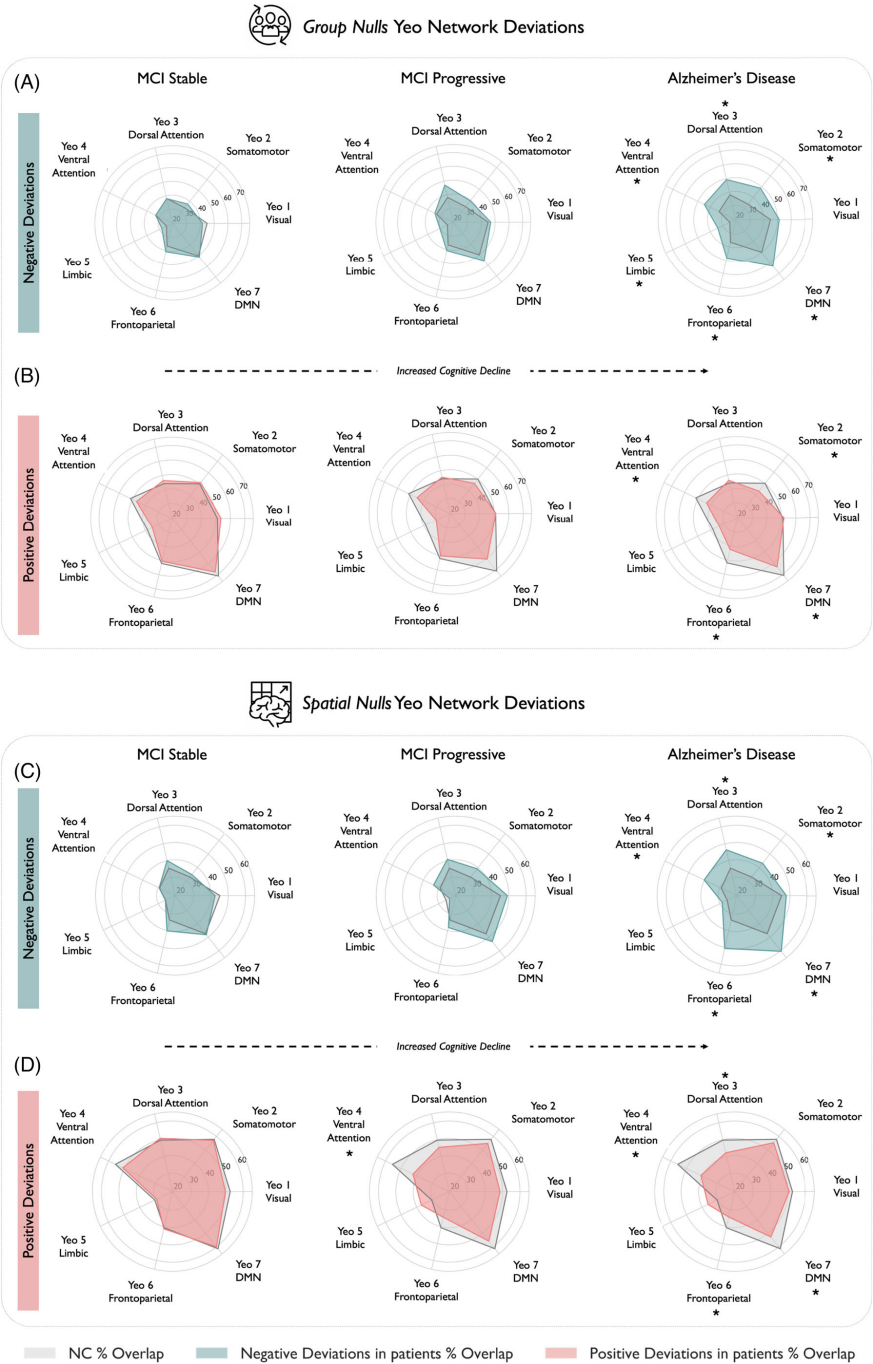
Network‐level enrichment of cortical deviations in AD and prodromal stages. (A and B) Group‐label permutation‐based deviation overlap for (A) negative and (B) positive deviations across the Yeo seven‐network parcellation. Radar plots display percentage of individuals in each diagnostic group (MCI stable, MCI progressive, AD) exhibiting at least one extreme deviation (|Z| > 1.96) within each network. (C and D) Equivalent results using spatially constrained null models that preserve the spatial autocorrelation of cortical maps. Deviations are computed relative to a held‐out control benchmark, with shaded regions representing differential overlap in patients versus controls. Asterisks indicate statistically significant differences (*p *< 0.05). AD, Alzheimer's disease; MCI, mild cognitive impairment.

We next assessed whether cortical deviations preferentially aggregated within each of the seven Yeo functional networks (Figures 2A–D). Compared to controls, AD showed markedly elevated overlap for negative deviations in most networks, including the default mode network (DMN), dorsal and ventral attention networks, frontoparietal network, and somatomotor network. The limbic network was significant for the group nulls, but not for the spatial‐null analyses, with the visual network not related to negative deviations in AD in either analysis. No significant differences were found when comparing either group of MCI patients to controls for negative deviations.

Positive deviations were more spatially diffuse, with both group‐ and spatial‐null models converging to show a significantly diminished overlap in AD compared to controls in ventral attention, somatomotor, DMN, and frontoparietal networks. Spatial‐null models also showed the same pattern for the ventral attention network in the MCI progressive group.

#### 3.3.1 Group‐level comparisons of deviation magnitude

**FIGURE 3:**
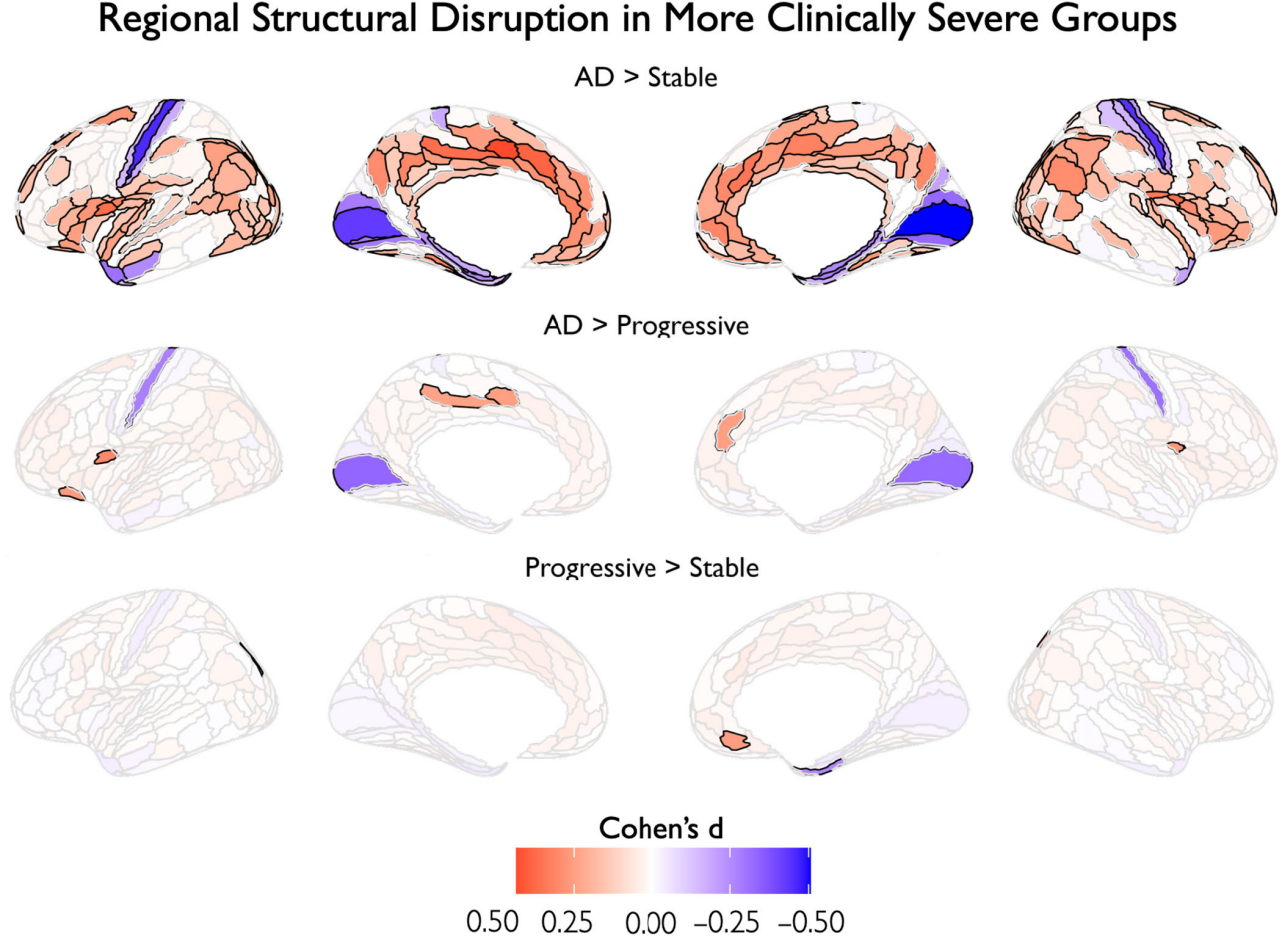
Cohen's *d* maps show pairwise diagnostic group differences in MIND deviation Z‐scores. Maps display Cohen's *d* effect sizes thresholded by FDR‐corrected significance (black borders). In these comparison maps, the colors are aligned with the Cohen's *d* effect sizes between the more severe and less severe clinical groups. Red (positive Cohen's *d*) indicates the more severe group has higher absolute Z‐scores for negative deviations (greater structural disruption), and blue (negative Cohen's *d*) indicates the more severe group has higher absolute Z‐scores for positive deviations (greater structural similarity). Effect sizes reflect three pairwise comparisons: AD versus MCI stable, AD versus MCI progressive, and MCI progressive versus MCI stable. AD, Alzheimer's disease; MCI, mild cognitive impairment; MIND, Morphometric Inverse Divergence.

To complement the individual deviation patterns shown in Figure 1, we performed pairwise diagnostic group comparisons using Cohen's *d* effect sizes. While Figure 1 quantifies each diagnostic group's deviations relative to controls, these analyses (visually shown in Figure 3) directly contrast clinical groups against each other to identify regions showing stage‐specific differences in deviation magnitude. In these comparison maps, the colors are aligned with the Cohen's *d* effect sizes between the more severe and less severe clinical groups. Red regions (positive Cohen's *d*) indicate the more severe group has higher absolute *Z*‐scores for negative deviations, reflecting greater structural disruption. Blue regions (negative Cohen's *d*) indicate the more severe group has higher absolute *Z*‐scores for positive deviations, reflecting greater structural similarity to controls relative to the less severe group. After FDR correction (full details in Table S3), Cohen's *d* effect sizes for deviations between key diagnostic comparisons showed that the largest effect sizes were observed, as expected in the comparison between AD and MCI stable (top row, Figure 3). For this comparison, widespread FDR‐significant effects were observed across medial temporal regions (e.g., bilateral PHA2 in the parahippocampal gyrus) and retrosplenial and posterior cingulate areas (e.g., bilateral 23c, Retrosplenial Cortex (RSC)). These disruptions extended into lateral and parietal cortices (e.g., POS2, PGi), ventrolateral prefrontal cortex (e.g., 47s, FOP3), and medial prefrontal regions (10v), closely tracking known hubs of the default mode and frontoparietal control networks. AD also showed significant disruption relative to MCI progressive in overlapping areas (middle row, Figure 3), though with much more limited spatial extent (e.g., 23c, 24v, and 47s in the left frontal lobe) and with blue color (indicating a negative Cohen's *d* capturing more positive deviations in AD than MCI progressive) in primary visual cortex (bilateral V1). Finally, MCI progressive individuals differed from the MCI stable group only in a region of the dorsal margin of inferior parietal cortex (IPO) and in the perirhinal entorhinal cortex in the temporal lobe. Overall, these results reinforce the graded nature of deviation accumulation across clinical stages, with the most robust structural disruptions emerging in regions linked to memory, executive function, and the default mode network.

These group‐level Cohen's *d* results should be interpreted with caution, as they assume homogeneity within diagnostic groups – an assumption we directly tested using Hamming distance analysis, which revealed moderate inter‐individual variability within each group. For negative deviations, median Hamming distances went from 7.0 in controls and MCI stable to 8.0 in MCI progressive and 11.0 in AD, with corresponding increases in interquartile ranges (IQR) (controls and MCI stable: IQR = 8.0; MCI progressive: IQR = 10.0; AD: IQR = 16.0). For positive deviations, median Hamming distances were as follows: 13.0 in controls (IQR = 17.0), 12.0 in MCI stable (IQR = 16.0), 12.0 in MCI progressive (IQR = 17.0), and 12.0 in AD (IQR = 20.0).

### 3.4 APOE analyses

Having established that MIND‐based structural deviations are sensitive to disease stage, we next examined whether these deviations also reflected underlying genetic risk. Specifically, we tested for associations between APOE genotype and the number of cortical regions exhibiting extreme deviations. For positive deviations, the basic additive model showed the best fit (AIC = 11,130.3), with interaction models providing no improvement (likelihood ratio tests: age interaction *p* = 0.407, sex interaction *p* = 0.367). For negative deviations, the sex interaction model was the best one (AIC = 10,397.7), representing a significant improvement over the basic model (likelihood ratio test: *p* = 0.040). The full three‐way interaction model (age*sex*deviations) showed no further improvements for either outcome (Table S4). The sex composition for the groups was as follows: ε3 homozygotes: *N* = 289 females and *N* = 267 males; ε2 carriers: *N* = 39 females and *N* = 54 males; ε4 heterozygotes: *N* = 283 females and *N* = 234 males; ε4 homozygotes: *N* = 62 females and *N* = 71 males.

**FIGURE 4:**
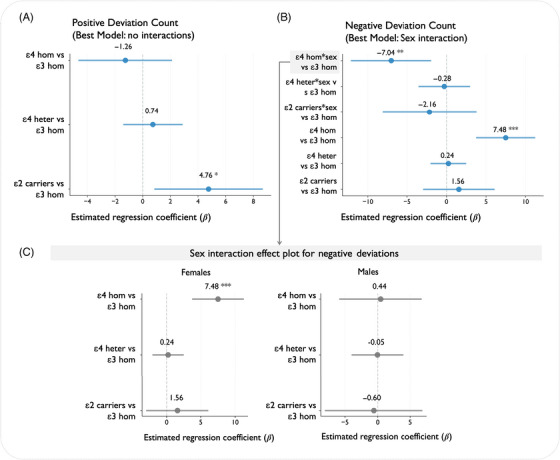
Apolipoprotein E (APOE) genotype effects on brain structural deviation counts. (A) Forest plot displays regression coefficients (*β* ± 95% CI) for positive deviation count from best‐fitting model (no interactions). All estimates are relative to ε3 homozygotes and adjusted for age and sex. (B) Regression coefficients from sex interaction model for negative deviation count, showing both main effects and interaction terms (APOE × sex). (C) Sex‐stratified effects for negative deviations. Points represent effect estimates with 95% CI. *p < 0.05, **p < 0.01, ***p < 0.001. CI, confidence interval.

For positive deviations, no interactions with age or sex were detected (Table S5). ε2 carriers exhibited significantly more positive deviations than ε3 homozygotes (*β* = 4.76, *p* = 0.016; Figure 4A). Neither ε4 heterozygotes (*β* = 0.74, *p* = 0.490) nor ε4 homozygotes (*β* = −1.26, *p* = 0.459) differed significantly from ε3 homozygotes.

For negative deviations, we observed a significant APOE*sex interaction (likelihood ratio test: *p* = 0.040), indicating sex‐specific APOE effects. Overall, ε4 homozygotes showed substantially more negative deviations than ε3 homozygotes (Figure 4B), and this effect was predominantly observed in females (*β* = 7.48, *p* < 0.001; Figure 4C). In males, the ε4 homozygote effect was markedly attenuated (sex interaction term: β = −7.04, *p* = 0.006), resulting in a minimal overall effect in this group (*β* = 0.44). No age interactions were detected for negative deviations (*p* = 0.42). All details for these models and tests can be found in Tables S5 and S6.

Additional sensitivity analyses (Supporting Information) using CT and GM revealed that all three metrics detected directionally consistent sex‐specific ε4 homozygotes effects in females, but with different levels of statistical significance when evaluated using matched model structures (MIND: *β* = 7.48, *p* < 0.001; GM: *β* = 5.46, *p* = 0.003; CT: *β* = 7.52, *p* = 0.052 not reaching significance). These findings suggest differences in statistical power or signal‐to‐noise characteristics across metrics, with MIND showing more robust detection of this genetic association. When allowing each metric to select its optimal model independently, CT and GM required more complex interaction structures but showed unstable parameter estimates. For the positive deviations, ε2 carriers exhibited significantly more positive deviations than ε3 homozygotes only for the MIND metric, but not when looking at the same analysis with CT or GM (CT: *β* = −2.08 (SE = 1.50), 95% CI = [−5.02, 0.86], *p* = 0.16; GM: *β* = 0.61 (SE = 1.62), 95% CI = [−2.57, 3.78], *p* = 0.71).

### 3.5 Negative deviations do not predict mortality risk in AD

**FIGURE 5:**
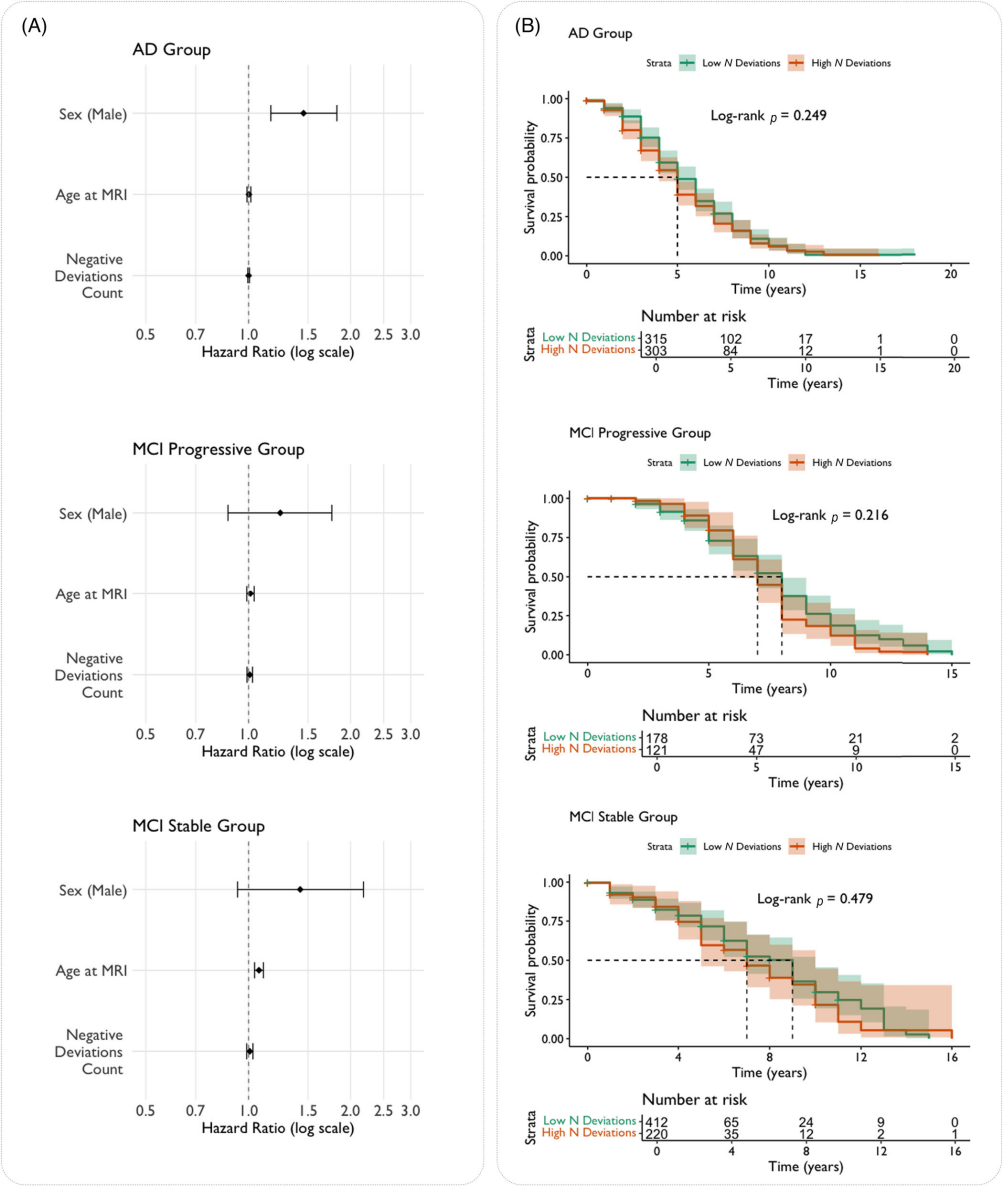
(A) Forest plots showing (HRs ± 95% CI) from separate Cox proportional hazards models for each diagnostic group, assessing the relationship between negative deviation count and survival. (B) Kaplan–Meier survival curves stratified by high versus low structural deviation burden (median split within each diagnostic group) for AD, MCI progressive, and MCI stable groups. Shaded areas denote 95% CI. Log‐rank *p* values indicate no significant survival differences between high and low burden groups in any diagnostic category. *p < 0.05, **p < 0.01, ***p < 0.001. AD, Alzheimer's disease; CI, confidence intervals; HR, hazard ratios MCI, mild cognitive impairment.

To assess whether individual structural brain deviations predicted survival, we ran two main analyses. The stratified Cox model revealed that continuous negative deviation count was not significantly associated with mortality risk (HR = 1.002, 95% CI: 0.997 to 1.007, *p* = 0.45). Separate Cox models for each diagnostic group (Figure 5A) confirmed that structural deviation burden was not predictive of mortality in any disease stage: AD (HR = 1.0005, 95% CI: 0.995 to 1.006, *p* = 0.86), MCI progressive (HR = 1.008, 95% CI: 0.990 to 1.027, *p* = 0.40), and MCI stable (HR = 1.008, 95% CI: 0.988 to 1.029, *p* = 0.43). Age at MRI showed the strongest prognostic value in MCI stable patients (HR = 1.073, 95% CI: 1.044 to 1.102, *p* < 0.001), while showing minimal effect in AD (HR = 1.002, 95% CI: 0.989 to 1.016, *p* = 0.71) and MCI progressive groups (HR = 1.013, 95% CI: 0.988 to 1.039, *p* = 0.31). Male sex remained a significant mortality predictor in AD patients (HR = 1.46, 95% CI: 1.17 to 1.81, *p* = 0.001) but not in MCI progressive (HR = 1.24, 95% CI: 0.87 to 1.76, *p* = 0.23) or MCI stable groups (HR = 1.42, 95% CI: 0.90 to 2.25, *p* = 0.11).

Kaplan–Meier survival curves stratified by high versus low structural deviation burden (divided at the cohort median) confirmed the Cox regression findings (Figure 5B). Log‐rank tests revealed no statistically significant survival differences between high and low burden groups in any diagnostic category (all *p* > 0.05), consistent with the continuous analyses.

### 3.6 Neurobiological decoding of normative MIND deviations

**FIGURE 6:**
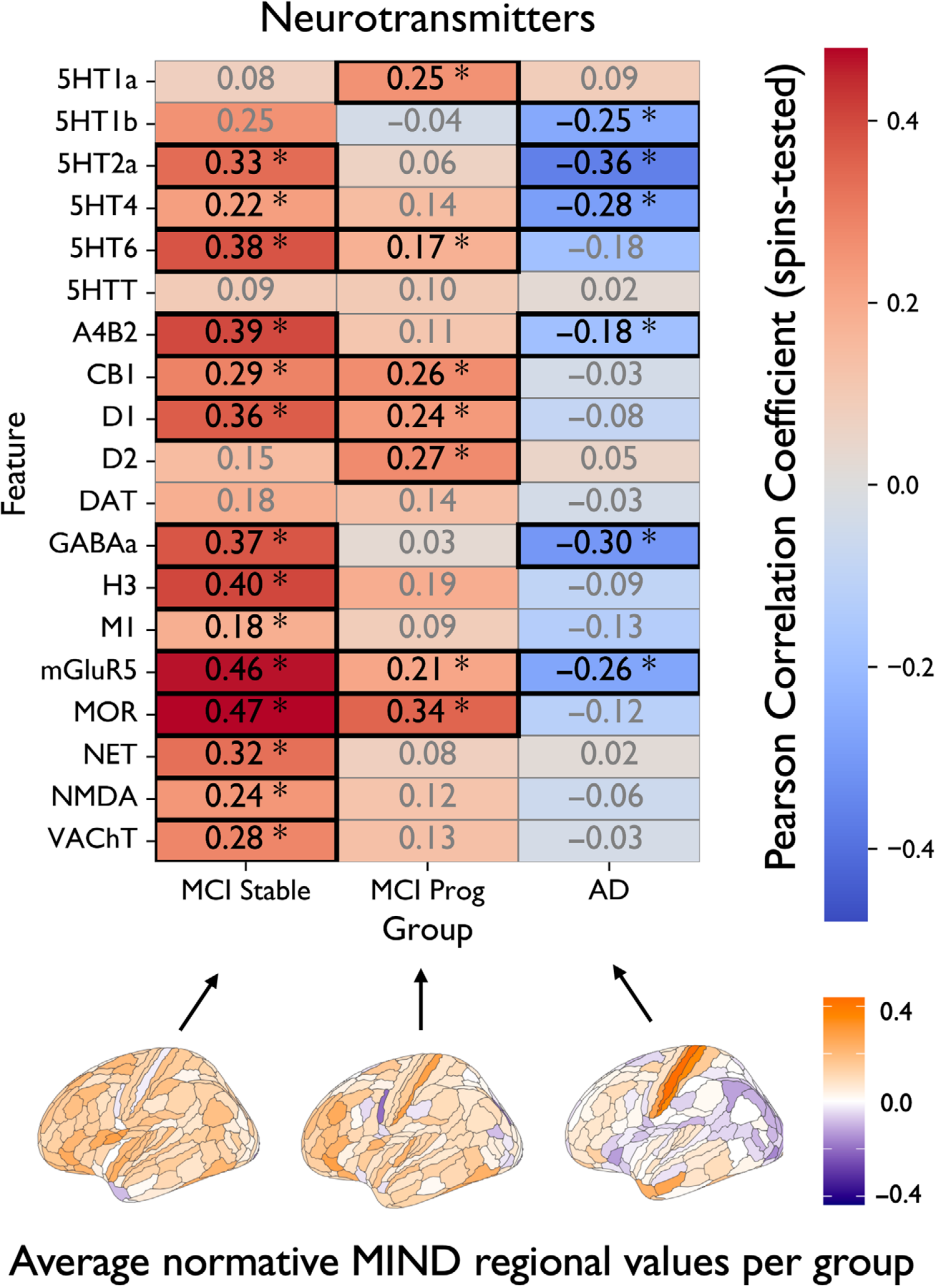
Biological decoding of group‐average deviation maps. Heatmaps show Pearson correlation coefficients (*r*) between average regional MIND deviation maps (*Z*‐scores) across both hemispheres for each diagnostic group (columns: MCI stable, MCI progressive, AD) and different neurotransmitter receptor density maps (rows). Correlations were tested for statistical significance using spin permutation testing (*N* = 5000) to control for spatial autocorrelation; significant values (*p* < 0.05, spin‐corrected) are marked with asterisks and outlined in black. Color indicates the direction and magnitude of the correlation (red = positive, blue = negative). Group‐average deviation maps are visualized below for reference with their corresponding legend. * Indicates statistically significant differences (*p* < 0.05). MCI, Mild Cognitive Impairment; MIND, Morphometric Inverse Divergence; AD: Alzheimer's disease

We assessed the spatial correspondence between regional normative deviations and different neurotransmitter density maps across diagnostic groups (Figure 6). When assessing different neurotransmitters, higher average deviations in the MCI stable group – characterized by predominantly positive *Z*‐scores (see corresponding brain plot at the bottom of Figure 6) – showed significant positive correlations with several receptor maps, including mGluR5 (*r* = 0.46, *p* < 0.001), MOR (*r* = 0.47, *p* < 0.001), GABAa (*r* = 0.37, *p* = 0.003), and multiple 5HT receptors. These associations were less strong in MCI progressive and were reversed in AD, where deviations were mostly negative. In fact, significant negative correlations in AD were observed with 5HT2a (*r* = −0.36, *p* = 0.001), 5HT4 (*r* = −0.28, *p* = 0.017), GABAa (*r* = −0.30, *p* = 0.006), and mGluR5 (*r* = −0.26, *p* = 0.011), indicating that regions where MIND decreased compared to what was expected from age‐ and sex‐matched healthy population references were partly spatially co‐localized with areas of higher normative serotonergic, GABAergic, and glutamatergic density.

Additional biological properties (such as microstructure and cytoarchitectonics) were also explored with additional maps as secondary analyses – for full details please see the detailed maps descriptions in the Supporting Information, with all the results shown in Figure S10 and Table S7.

### 3.7 Prognostic analysis: more negative deviations are linked to higher *post mortem* pathology

**FIGURE 7:**
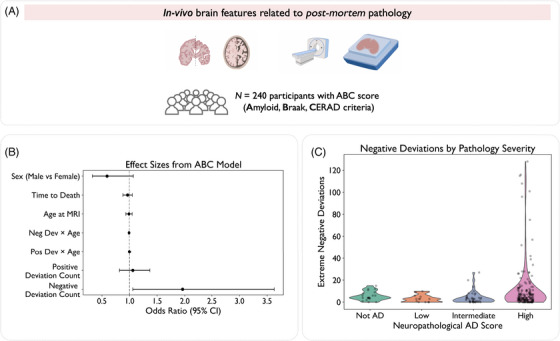
Structural deviation burden is associated with neuropathological severity. (A) Overview of subsample (*N* = 240) from NACC with in vivo magnetic resonance imaging and subsequent ABC scoring (amyloid, Braak, and CERAD criteria). (B) Forest plot showing odds ratios and 95% confidence intervals from the ordinal logistic regression model predicting ABC score. (C) Violin plots of extreme negative deviations across neuropathological severity levels (where neuropathological AD score refers to the ABC Scores). CERAD, Consortium to Establish a Registry for Alzheimer's Disease; NACC, National Alzheimer's Coordinating Center.

We investigated whether the burden of structural deviations was associated with neuropathological severity in a subset of *N* = 240 participants for whom autopsy data were available in NACC (schematics shown in Figure 7A, with further details in Table S8 and distribution of ABC scores across diagnostic categories shown in Figure S11). Ordinal logistic regression revealed a significant association between the total number of extreme negative deviations and higher ABC score (Figure 7B). The best‐fitting model included an interaction term with age at MRI (AIC = 427.8), indicating that the relationship between structural disruption and AD pathology was age‐dependent. Specifically, the main effect of negative deviations was significant (OR = 1.96, 95% CI: 1.06 to 3.63, *p* = 0.032) with an additional negative interaction with age (*p* = 0.041). In contrast, the number of extreme positive deviations was not associated with ABC score in any models (all *p* > 0.1), and time‐to‐death interactions did not improve model fit. Details of all models can be found in Table S9, with stratified model results shown in Table S10.

The distribution of deviation burden across ABC pathology levels is shown in Figure 7C. Diagnostic stratification showed that this effect generalized across AD and progressive MCI but was attenuated in MCI stable participants, consistent with lower pathology burden in that group. Exploratory models examining each A, B, and C score as outcomes did not reveal consistent associations with deviation count and are reported in Table S11.

As additional sensitivity analyses, we repeated the ordinal logistic regressions using CT and GM deviation counts (Supporting Information). All three metrics showed associations with neuropathology in their optimal model specifications, though these differed. When using matched model structures (age interactions) across all metrics, only MIND maintained significant associations (*p* = 0.032), while CT (*p* = 0.670) and GM (*p* = 0.384) did not reach significance. However, when allowing each metric to independently select its optimal model via AIC, both CT (AIC = 417.2) and GM (AIC = 423.6) achieved significance through time‐to‐death interaction models (both *p* < 0.01).

## 4 DISCUSSION

### 4.1 Summary of key findings

This study presents a comprehensive integration of MIND networks with hierarchical Bayesian normative modeling for AD and one of the first systematic clinical applications of MIND in dementia. Our framework reveals individual‐level neuroanatomical changes relevant to disease progression and biological vulnerability.

Our main findings show that negative deviations were highly sensitive to different aspects of AD pathology, effectively stratifying clinical stages. The number of negative deviations increased progressively from individuals with stable MCI, to those with progressive cognitive decline, and finally to those with a diagnosis of AD. Our APOE ε4 analyses also revealed strong sex‐specific effects, with ε4 homozygote females showing substantially more negative deviations than other genotypes. Spatially, deviations co‐localized with neurotransmitter receptor density maps. For prognosis, although there was no significant effect between deviations and mortality, individuals with more negative deviations showed greater *post mortem* neuropathological burden, establishing a direct link between our in vivo neuroimaging markers and underlying disease pathology. Importantly, our overlap analyses revealed low regional concordance across individual patients (<10%), indicating substantial individual heterogeneity that traditional group‐average approaches would overlook. This heterogeneity suggests that clinical applications may benefit from individualized deviation approaches, while highlighting the value of population modeling for detecting meaningful patterns amid this individual variability. Complementary evidence for the clinical relevance of morphometric similarity metrics also comes from recent lifespan work^18^ that used an alternative normative modeling framework to chart MIND across the lifespan and to detect disorder‐specific deviations.

Additionally, we conducted systematic comparisons with univariate measures. While deviations using CT and GM were also useful in identifying dementia‐related changes, MIND showed some improvements by detecting early‐stage clinical separation and demonstrating more robust sex‐specific genetic associations, suggesting complementary clinical utility across metrics (with some caveats discussed in our limitations).

### 4.2 Disrupted cortical integration in AD

Our findings contribute to evidence that AD reflects not only regional atrophy but also disrupted cortical organization.^62^, ^63^ The predominance of negative deviations, indicating that regions are becoming less morphometrically similar to the rest of the cortex than expected is consistent with a potential alteration in the coordinated structural organization of the cortex. Notably, significant negative deviations were predominantly located in association areas along the sensorimotor‐association axis,^58^ while positive ones were more in primary sensory regions. Our findings may reflect a disruption of homophilic cortical architecture, i.e., the principle that structurally and transcriptionally similar regions are preferentially aligned and coordinated in the brain.^4^ While further validation is needed, these results support the view that structural disintegration at the network level may be a relevant dimension of Alzheimer's pathophysiology.

The presence of more negative deviations, particularly in the default mode, attention, and frontoparietal networks, supports the network degeneration hypothesis.^64^ However, our findings also demonstrate selective vulnerability, with a contrast between affected somatomotor networks and spared visual networks – potentially suggesting that MIND‐based measures detect disruption of structural alignment in systems that are otherwise considered more functionally resilient in dementia. Importantly, while our regional overlap analyses showed low rates across individual patients, network‐level analyses revealed that when regions were grouped into functional systems, preferential targeting patterns emerged. This suggests that AD may affect specific networks consistently, but with individual variation in which regions within those networks show deviations. This pattern of network‐level consistency amid regional and inter‐individual heterogeneity would be missed by traditional approaches that focus on group‐average regional effects.

Spatial co‐location analyses provided biological context for our results. In AD, regions with *decreased* morphometric similarity overlapped with areas of high serotonergic (5‐HT2a, 5‐HT4), GABAergic, and glutamatergic (mGluR5) receptor density. Although these represent group‐averaged receptor maps, these findings are consistent with emerging evidence for neurotransmitter system dysfunction in AD^65^, ^66^ and suggest that MIND‐based deviations may be sensitive to disruption in regions supporting modulatory network coordination.^48^ Interestingly, in MCI stable participants, we observed the opposite pattern: regions with higher‐than‐expected structural similarity aligned with areas rich in mGluR5, MOR, and GABAergic receptors (alongside many others, spanning from nicotinic acetylcholine receptor to one dopaminergic receptor and more). Overall, this pattern of positive deviations in MCI stages shifting to negative deviations in AD, may reflect a transition from structural resilience to breakdown as the disease progresses, although no direct causal link can be made here. The observed results suggest that structural deviations can be biologically decoded by taking into account underlying neurotransmitter systems.^48^, ^59^

In secondary exploratory analyses, we observed stage‐specific relationships with cortical organizational properties including evolutionary expansion and allometric scaling from our Supporting Information. These results, although exploratory, are consistent with the “last‐in, first‐out” principle,^67^ whereby brain regions that develop relatively late during adolescence also show accelerated degeneration in aging and heightened vulnerability to neurodegenerative diseases.^67^

### 4.3 Genetic architecture and sex‐specific vulnerability

Our findings reveal marked sex specificity of APOE ε4 effects on negative deviations: sex‐specific vulnerability in the form of increased number of negative deviations among ε4 homozygous females. This aligns with evidence that APOE ε4 risk for AD is greater in women^68^, ^69^ and could not be explained by sample imbalances, suggesting fundamental differences in how genetic risk manifests structurally. Sensitivity analyses revealed this sex‐specific ε4 homozygote effect was also present when looking at negative deviations in CT and GM models, although the results showed similar magnitudes but different statistical robustness. Interestingly, ε2 carriers showed significantly elevated positive MIND deviations compared to ε3 homozygotes, potentially representing some neuroimaging evidence of ε2's protective effects at the network level (this was not observed with CT and GM). While the underlying mechanisms remain to be clarified, this finding is consistent with evidence for ε2's protective role in AD.^70^

### 4.4 Prognostic implications and clinical translation

The association between structural deviations and *post mortem* neuropathological severity suggests that morphometric similarity networks may hold promise as marker of disease burden, although using negative deviation count from univariate metrics was also found to relate to neuropathology. All these measures could contribute to more refined patient stratification in research settings and inform individualized assessments. The prognostic value of our normative modeling‐derived measures is consistent with recent longitudinal evidence that higher outlier burden over time predicts clinical dementia progression.^19^ Further validation is required to determine appropriate thresholds for clinical use, assess reproducibility across diverse cohorts and imaging protocols, and establish how these measures compare to other imaging biomarkers. While we also present group‐level analyses to validate our approach, the core strength of our framework lies in calculating individual‐level deviation maps. This directly addresses AD heterogeneity and enables precision medicine approaches for personalized patient assessment and prognosis.

### 4.5 Limitations and future work

Several limitations should be acknowledged. First, while our approach reveals individual‐level deviations at single time points, it prevents direct assessment of temporal changes. Longitudinal studies are necessary to clarify how MIND‐based deviations evolve over time and establish their prognostic value, as shown for traditional morphometric measures.^15^ Second, the choice of morphometric features inevitably influenced network construction; using alternative features might provide different biological insights. Although we selected our features based on those widely used in previous work,^5^, ^7^, ^71^ it remains an open question as to how many and what would be the “optimal” choice.^4^ A related limitation is that we only used T1‐derived FreeSurfer measures. It is increasingly recognized that other MRI measures, such as diffusion MRI or myelin‐sensitive imaging, may provide a more sensitive index of disease. While our choice ensured compatibility across datasets and with prior work, the MIND framework could incorporate such modalities in future studies to capture additional dimensions of disease‐related change. Additionally, MIND networks were restricted to cortical regions, and it would be useful to extend analyses to the subcortex.^72^ Our cohort also consisted primarily of individuals of European ancestry, potentially limiting generalizability to other populations and requiring validation across diverse groups before broad clinical application. Related to this, validating our combined normative MIND approach on portable low‐field MRI scanners^73^ will be important to ensure its accessibility and scalability in real‐world clinical settings, supporting broader adoption across diverse healthcare environments. Crucially, while our neuropathological and spatial co‐location results are encouraging, more direct validation through *post mortem* studies or molecular imaging would strengthen mechanistic interpretations. Finally, the sensitivity analyses comparing our results with univariate measures suggest that metric choice may depend on the research question. MIND showed modest but consistent advantages (higher model fit, unique separation of MCI progressive versus controls, and more robust sex‐specific APOE effects), but CT and GM provided strong discrimination for AD and were valuable for characterizing dementia‐related changes. For applications with a focus on genetic associations or early clinical stratification, MIND's multivariate approach may offer statistical advantages. However, for simpler descriptive analyses or when computational resources are limited, modeling of univariate metrics may be preferred.

## 5 CONCLUSION

This study introduces a novel hybrid framework that integrates MIND networks with hierarchical Bayesian normative modeling to map individual‐level deviations in cortical structure. Our multivariate approach captures biologically grounded markers of individual brain disruption in AD and not only stratifies disease stages and genetic risk but also links neuroanatomical variation to severity of *post mortem* pathology. Taken together, these findings highlight the promise of multivariate, network‐based normative models as scalable tools for precision mapping of brain disorders.

## CONFLICT OF INTEREST STATEMENT

Jakob Seidlitz holds equity in and is a director of Centile Bioscience. Richard A.I. Bethlehem holds equity in and is a director of Centile Bioscience. All other authors report no disclosures relevant to the manuscript. All author disclosures are available in the Supporting Information.

## CONSENT STATEMENT

All data collection protocols were approved by the Institutional Review Board of each cohort, and informed written consent was obtained from all participants in the original studies.

## Supporting information



Supporting Information

Supporting Information
