# Supplementary material for: Structural similarity networks reveal brain vulnerability in dementia
Source: Alzheimers Dement. 2025 Dec 26;21(12):e70973. doi: 10.1002/alz.70973 (PMC12741943; doi:10.1002/alz.70973)
Supplement: Supplementary file 1 — Supporting Information [file ALZ-21-e70973-s001.docx]

**Supplementary Material**

## **Normative Modelling Implementation and Quality Controls**

**Model Configuration and Specifications**

We implemented a hierarchical Bayesian regression model using the PCN toolkit (<https://github.com/amarquand/PCNtoolkit> ) with a specific configuration to optimize performance and balance model complexity. The model used *bspline* basis functions with linear terms for both mean (μ) and variance (σ) parameters. We included random intercepts for the mean to appropriately account for site effects, while excluding random slopes and sigma random effects to maintain model parsimony and stability. For sampling, we utilized the No-U-Turn Sampler (NUTS) with these specifications: Number of chains: 4; Samples per chain: 5000; Tuning samples: 2000; Target acceptance rate: 0.99. We trained normative models on Morphometric INverse Divergence (MIND) weighted degree values from 35,133 UK Biobank participants to serve as our reference dataset. For the NACC dataset, we assigned controls to both the transfer/adaptation set (HCtrain; N=1624) and the test set (HCtest; N= 394) sets in an 80:20 ratio. The HCtrain set was used for model recalibration, while the HCtest set along with patient groups (AD: N=624; MCI Stable: N=643; MCI Progressive: N=300) were used for assessing deviations from normative brain structure.

**MCMC Convergence Assessment**
To assess the convergence of our HBR normative models, the Gelman–Rubin statistic ($\hat{R}$) was computed for all model parameters in line with what recommended for the PCNToolkit (see available tutorial online at the following link: <https://github.com/predictive-clinical-neuroscience/PCNtoolkit-demo/blob/81967c1e158feeaa3651aed6decee80f3018a563/tutorials/HBR_SHASH/HBR_Tutorial.ipynb>). $\hat{R}$ quantifies agreement between chains by comparing within-chain and between-chain variance. Ideal convergence corresponds to an $\hat{R}$ value of 1.00, while values below 1.05, or a more stringent 1.01 used here, are widely accepted as evidence of good convergence (Vehtari et al., 2021). As a representative example, we visualized $\hat{R}$ values across all parameter groups for one model (see Supplementary Figure 1). Most parameters across mean and variance components had $\hat{R}$ values well below 1.01, indicating good mixing. A small number of early spline coefficients (e.g., in sigma_intercept_mu and offset_slope_mu) slightly exceeded 1.01 but remained below 1.05, likely reflecting minor local uncertainties rather than substantive convergence failures. No parameters showed evidence of non-convergence ($\hat{R}$ > 1.1). These findings confirm the overall stability and reliability of the posterior estimates used in downstream analyses.





**Supplementary Figure 1-** $\hat{R}$ convergence plots for all model parameters from one representative ROI model. The red line indicates the $\hat{R}$= 1.01 threshold. Most parameters show values at or near 1.00.

**Data Preparation and Site Handling**

To facilitate proper handling of multi-site data, unique site identifiers were assigned to each location, creating batch effect matrices for both the training and test sets. This approach allowed the model to parse out site variability effectively while maintaining the ability to detect true biological differences. All sites included in the test set were also represented in the training set to ensure proper calibration of site-specific parameters.

**Model Performance Evaluation**

We evaluated the explained variance of the model to assess its performance across regions. Variance plots were generated to verify the distribution of Explained Variance (which quantifies the proportion of variance in the ROI that is explained by the model) and Standardized Mean Squared Error (measures the average squared difference between the predicted and observed values, standardized by the variance in the data). See Supplementary Figure 2.


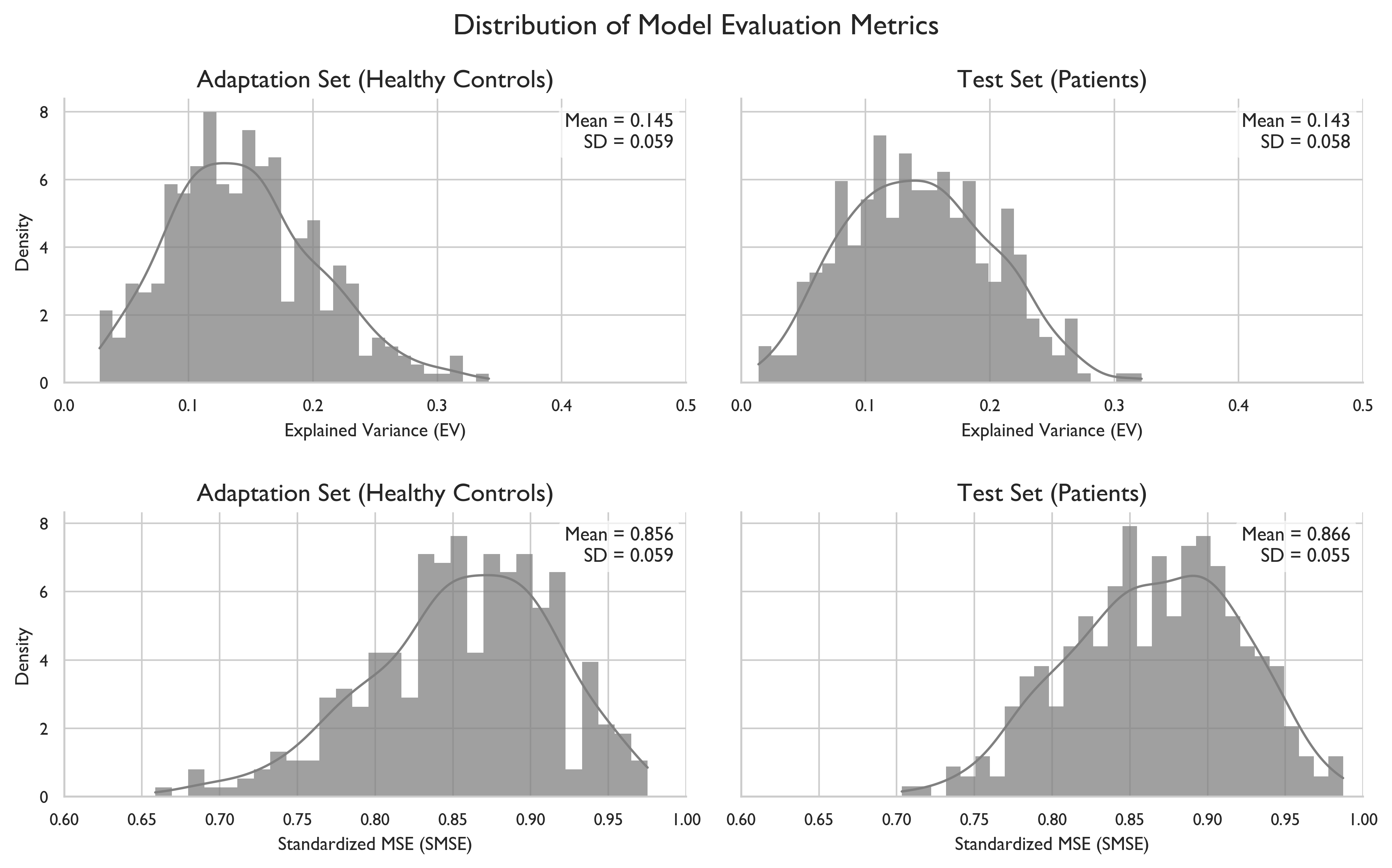


**Supplementary Figure 2**- Distribution of Model Evaluation The distributions of i) explained variance (EXPV; higher is better) in the top two rows, and ii) Standardized mean squared error (SMSE; lower is better) in the lower panel, across 360 ROIs in the trasfer/adaptation sample and in the test cohort.

We generated age distribution density plots for controls in the UK Biobank training set and both the adaptation/transfer set (HC_train_) and test set of the NACC dataset (AD, MCI Stable, MCI Progressive, HC_test_) to ensure appropriate age coverage across cohorts. See panels in Supplementary Figure 3 below. We also plotted age distributions by site and gender for both sets to ensure no demographic or site-related imbalances. In addition, site-wise distributions of participants’ age in the adaptation and test samples were plotted for each site. See Supplementary Figure 4.


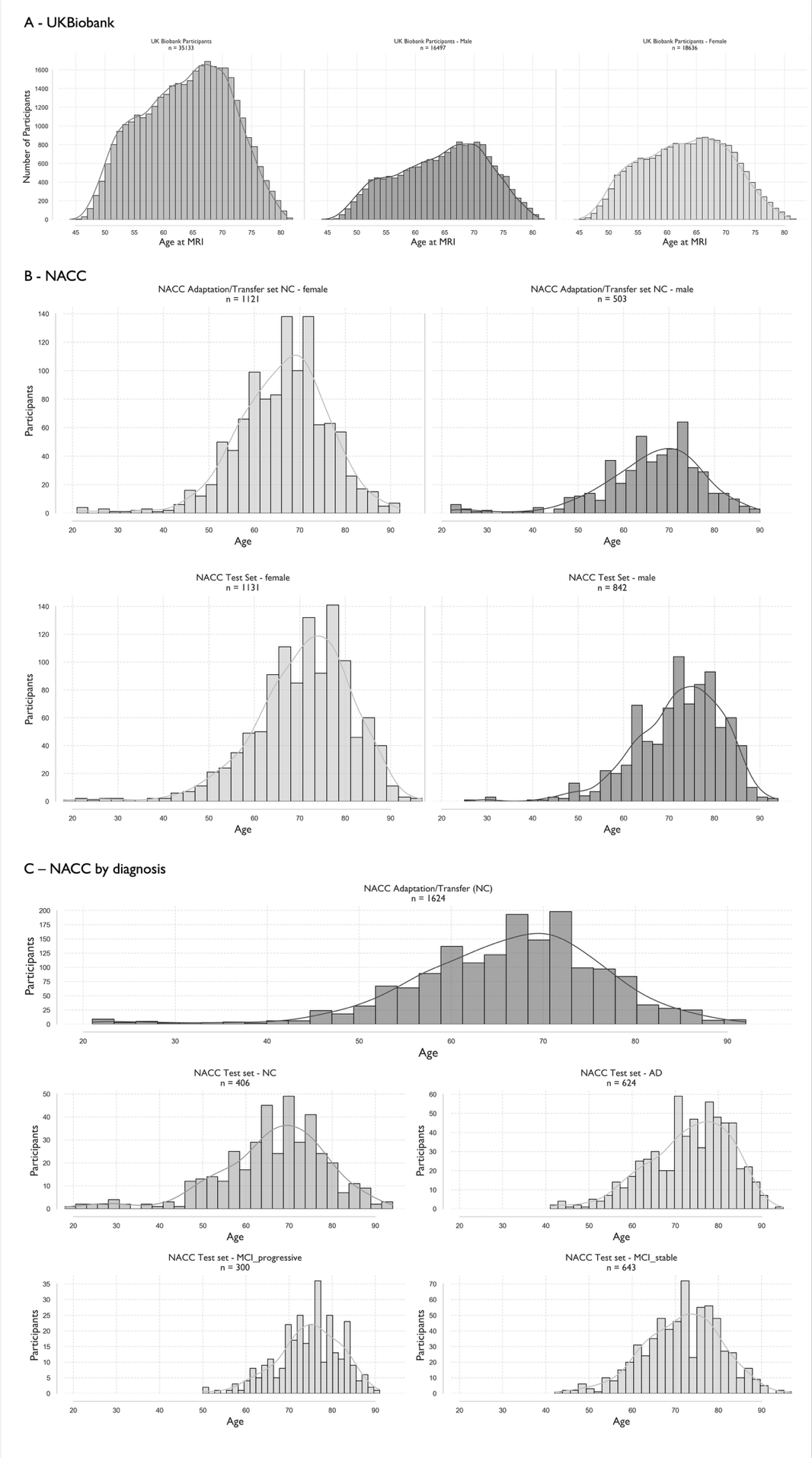


**Supplementary Figure 3** – (**A)** Histograms showing age distributions of participants from the UK Biobank normative model training set, stratified by sex. **(B)** Age distributions for the NACC adaptation (transfer) and test cohorts, stratified by sex. Kernel density estimates (KDEs) are overlaid to visualize distribution shape. **(C)** Age distributions in the NACC test set stratified by diagnostic group: Alzheimer's Disease (AD), Mild Cognitive Impairment (MCI; stable and progressive), and cognitively normal controls. These plots confirm appropriate age coverage and diagnostic representation in the model adaptation and test sets.


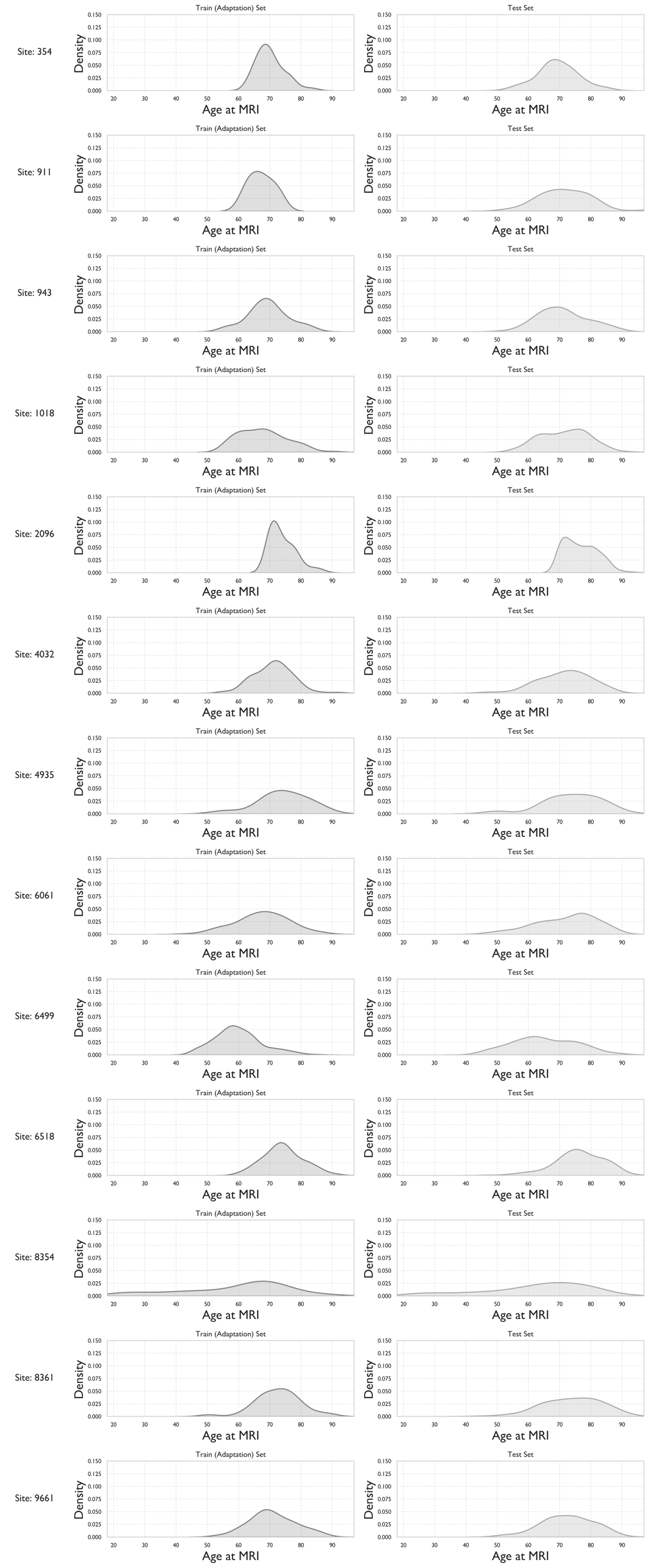


**Supplementary Figure 4**- Site-wise distributions of participant age at MRI scan in the adaptation and test NACC cohort. For each site, the kernel density estimate (KDE) of age is shown separately for the adaptation set (on the left) and test set (on the right).

**Controlling for Total Intracranial Volume in Normative Modelling**

Initial analysis revealed significant correlations between estimated Total Intracranial Volume (eTIV) and the number of outlier regions (defined as z-scores exceeding ±1.96) across diagnostic groups. These correlations could potentially confound interpretation of disease-related abnormalities since variations might be partly attributable to head size rather than pathology. See Supplementary Figure 5 below.


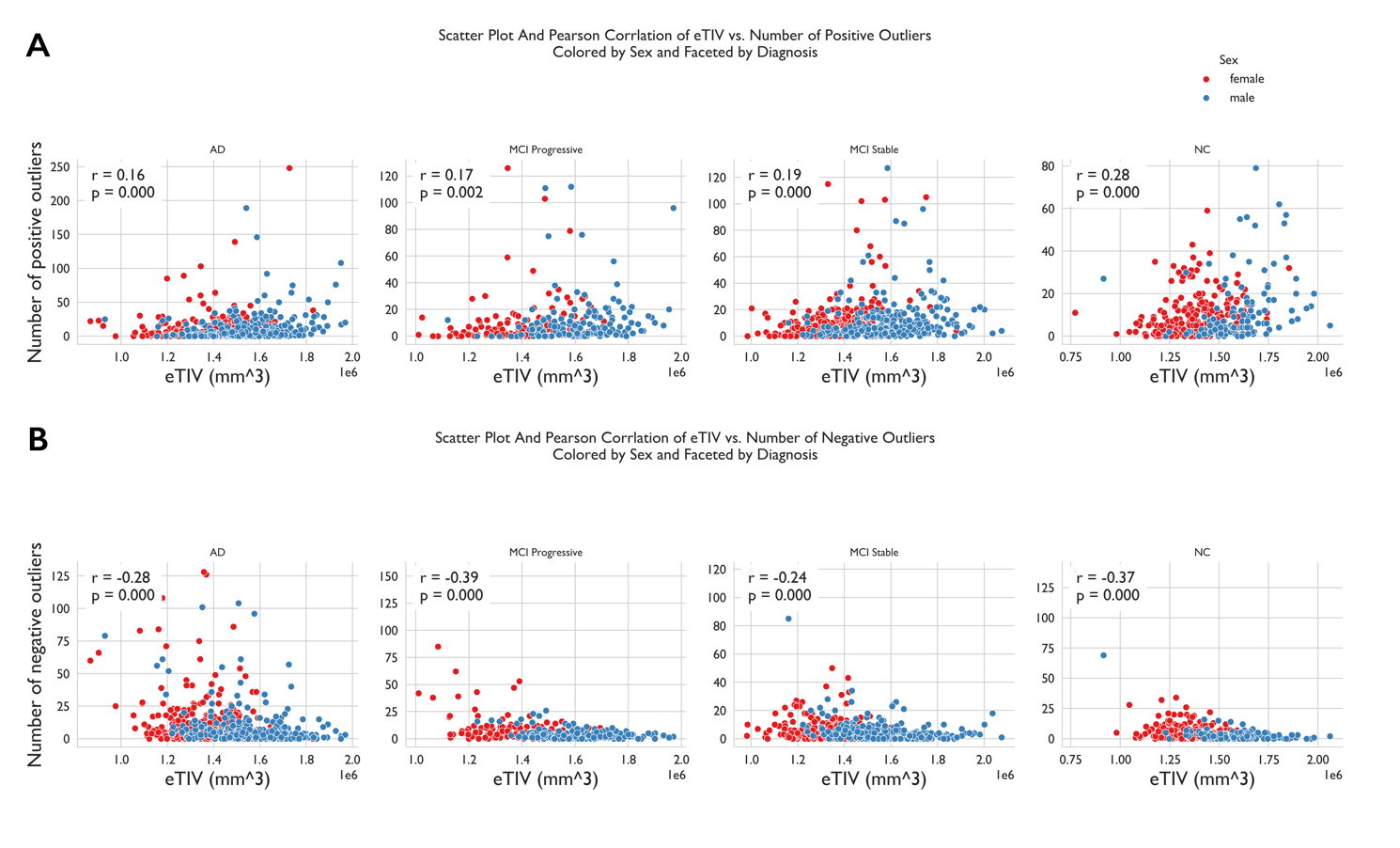


**Supplementary Figure 5** - **Correlation between estimated total intracranial volume (eTIV) and cortical deviation burden across diagnostic groups.** (A) Scatter plots showing the relationship between eTIV and the number of extreme positive deviations (Z > 1.96) for each diagnostic group: Alzheimer's Disease (AD), MCI Progressive, MCI Stable, and cognitively normal controls (NC). Points are coloured by sex, and Pearson correlation coefficients are reported per group. Significant positive correlations were observed in AD and MCI Progressive groups, suggesting that head size may confound deviation estimates if uncorrected. (B) Equivalent plots for negative deviations (Z < –1.96). Correlation strengths and directions vary by group and sex, reinforcing the necessity of residualizing regional deviation scores for eTIV to mitigate bias.

We first ensured removal of outliers in eTIV for each diagnosis and sex (defined as being outside the IQR range). This led to the removal of 19 females (AD: 5, MCI Stable: 9, NC: 5) and N=11 males (AD: 1, MCI Progressive: 1, MCI Stable: 2, NC: 7). We subsequently performed residualisation separately for each demographic group (defined again by sex and diagnosis) to account for potentially different relationships between brain measures and head size across these populations. For each brain region and demographic group, we fit a linear regression model predicting the regional z-score from eTIV, calculated residuals (observed z-score minus predicted z-score), added back the group mean to maintain the original scale and interpretability, and verified the effectiveness of residualisation by testing for remaining correlations. See Supplementary Figure 6 below.


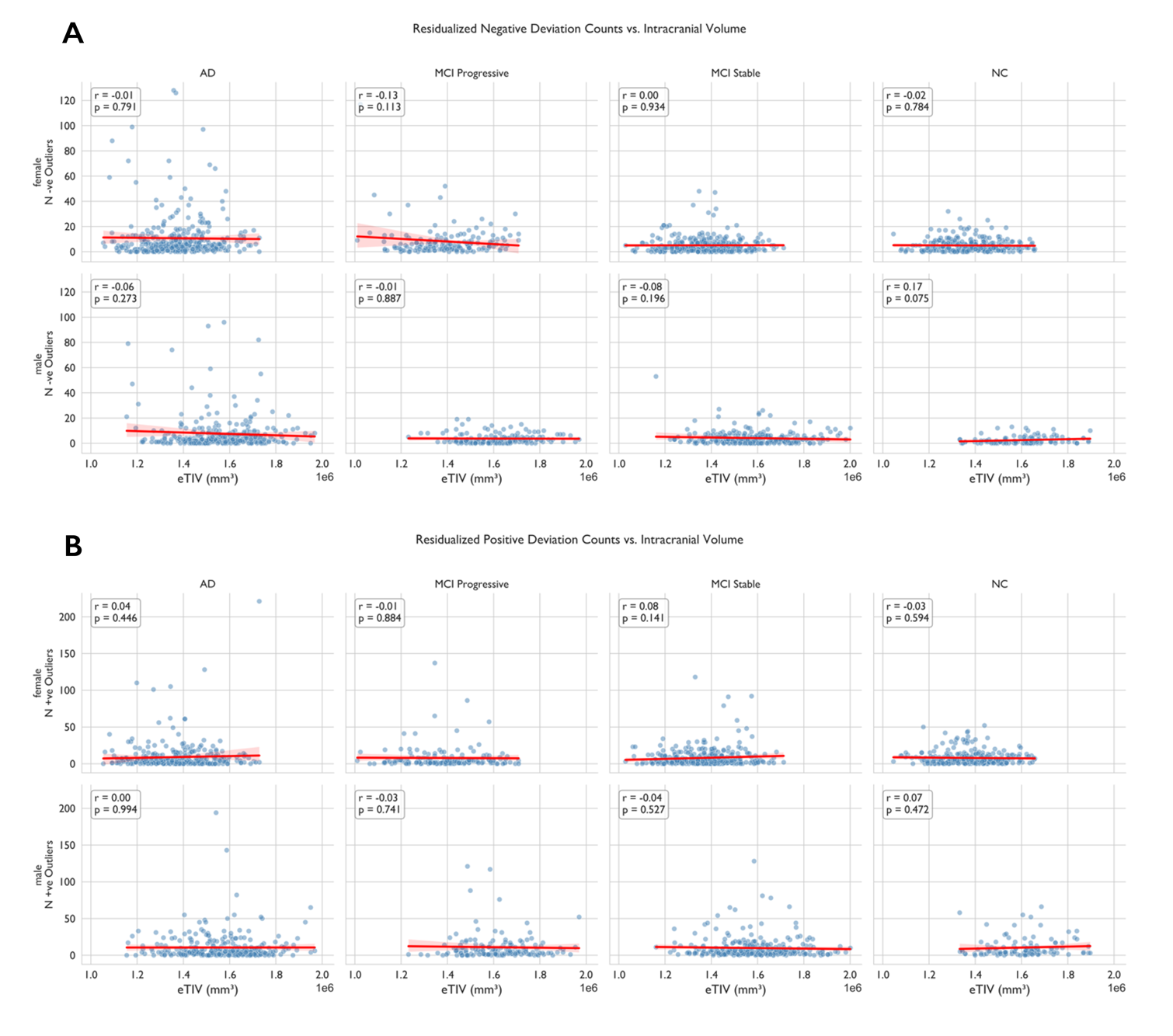


**Supplementary Figure 6**- Relationship between residualised deviation counts and intracranial volume after eTIV correction. (A) Scatter plots showing the association between eTIV and residualised negative deviation counts (Z < –1.96) across diagnostic groups following group- and sex-specific eTIV adjustment. Each panel displays Pearson’s correlation coefficient (r) and corresponding p-value. No significant associations were observed, confirming effective removal of eTIV-related confounding. (B) Equivalent analysis for residualised positive deviation counts.

This group-specific approach was chosen over global residualisation because relationships between brain structure and head size often differ between males and females. After residualisation, the correlation between eTIV and deviation counts was substantially reduced (average correlation coefficient reduction: 0.24 for positive deviations, 0.27 for negative deviations), with most correlations no longer statistically significant. The residualised z-scores provide a more specific representation of disease-related brain abnormalities, adjusting for the confounding effect of head size while preserving the interpretability of the normative modelling framework.
 **Evaluation of Age-Related Bias in Deviation Estimates**

Lastly, we assessed the correlation between age and deviation burden, quantified as the mean absolute Z-score across all regions of interest, in the final test set. This was done to evaluate whether model predictions were biased by age, particularly at the extremes of the age distribution, where model uncertainty tends to be greater due to fewer observations. Results indicated no significant correlation between age and deviations (Pearson’s *r* = -0.026, *p* = 0.250), suggesting that individual-level deviation estimates were not systematically influenced by age-related factors.

## **Relationship Between MIND Networks and Morphometric Features**

To investigate the anatomical correlates of MIND-derived deviation maps, we computed regional Pearson correlations between the weighted degree values for MIND (i.e., the MIND values before modelling) and the five morphometric MRI features derived from FreeSurfer that were used to construct the MIND networks, namely: cortical thickness (CT), mean curvature (MC), sulcal depth (SD), surface area (SA), and grey matter volume (GMV), across 360 Glasser regions and separately for each diagnostic group (Healthy Controls (NC), MCI Stable, MCI Progressive, and AD). These features were chosen in line with previous work. As shown in Supplementary Figure 7 below, the strongest and most consistent positive associations were observed with GMV (r between 0.43 in controls to 0.46 in AD) and SA (r from 0.36 to 0.41), and by robust negative correlations with SD (r between -0.40 and -0.45). These effects were statistically significant across all groups, with FDR-corrected p-values. Correlations with MC (r between 0.24 and 0.28) and with CT (r between 0.11 and 0.13) were weaker but remained significant. The only non-significant feature was CT in the AD group. These findings support the anatomical interpretability of MIND networks.


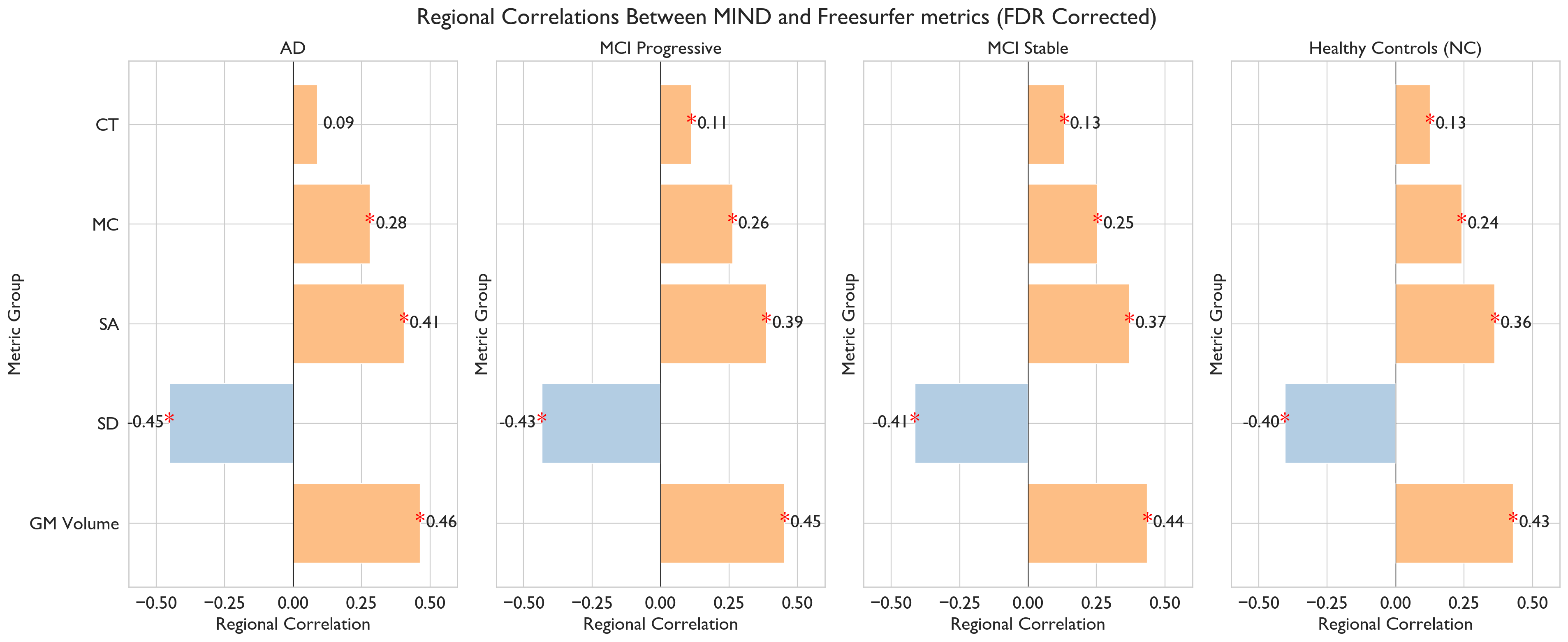


**Supplementary Figure 7** – Pearson Correlations Between MIND and morphometric MRI Metrics derived from Freesurfer and used to construct the MIND networks. Asterisks (*) indicate significant correlations (P-val after FDR correction).

## **Comparison with grey matter volume and cortical thickness univariate measures**

Sensitivity analyses were done to assess whether MIND provides added value over simpler univariate metrics. For this aim, we conducted comprehensive parallel analyses repeating the complete normative modelling framework using cortical thickness (CT) and grey matter volume (GM) as inputs. This involved training entirely separate normative models for each metric on UK Biobank data (N=35,133 scans) and on the same NACC transfer/adaptation set and the test set. The NACC training set was used for model recalibration, while the test set of controls along with patient groups was used for assessing deviations from normative brain structure. The computational scope was substantial: training two additional complete sets of region-specific normative models (2 metrics × 360 regions = 720 independent models), followed by model adaptation and deviation quantification in the clinical sample – all following the same exact procedure detailed in **Supplementary Section 1**.

Overall, these findings demonstrate that all three metrics are useful. They all effectively stratify diagnostic groups, with some differences in statistical properties. While cortical thickness showed the largest overall effect size for diagnostic discrimination using negative deviations burden, MIND uniquely detected significant differences between MCI-Progressive and controls, a clinically important early-stage distinction. MIND was more statistically robust for detecting sex-specific APOE4 effects. The three metrics all show overlapping but also distinct spatial patterns and, while all show associations with aggregate neuropathology outcomes in their optimal models (via AIC-based model selection), they do so in different ways (age-modulated vulnerability for MIND vs time-to-death for the univariate features), suggesting again that they capture overlapping but not identical biological signals.

**3.1 Proportion of explained variance for the different models**

We compared explained variance (EXPV) of the normative models themselves across metrics. In the test set (N=1,943 including patients and controls), MIND showed the highest average explained variance (0.143±0.058) compared to cortical thickness (0.126±0.065) and grey matter volume (0.109±0.050) - **Supplementary Figure 10.** These findings indicate on average a small but higher explained variance across ROIs when using the MIND metric compared to univariate CT and GM metrics.


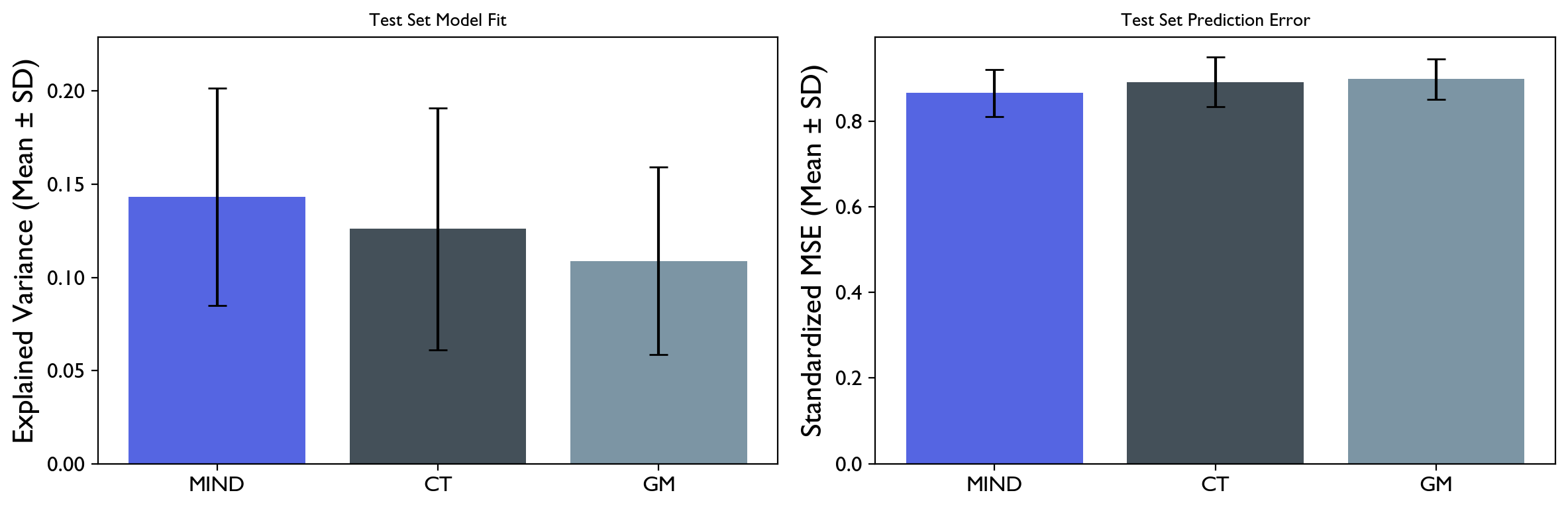


**Supplementary Figure 8** **- Normative model fit comparison across metrics.** Explained variance (EXPV) computed for each of 360 cortical regions. Higher EXPV indicates better model fit. In the test set (patients and controls; N=1,943), MIND demonstrated superior per-region fit. Values represent mean ± SD across regions.

**3.2 Clinical Discriminability**
We assessed each metric's ability to stratify diagnostic groups (AD, MCI Progressive, MCI Stable, Controls) using one-way ANOVA on negative deviation counts (number of regions with z < -1.96), followed by Tukey HSD for pairwise tests. All three metrics showed significant group effects, with (p<0.001) with CT showing the largest effect size (MIND F(3,1939)=24.39, η²=0.036, p<0.001; CT F(3,1939)=46.37, η²=0.067, p<0.001; GM F(3,1939)=38.45, η²=0.056, p<0.001).

**Tukey HSD pairwise contrasts
(Δ = difference in mean negative-deviation counts; 95% CI; adjusted pval):**

- **MIND**
  - **AD > MCI-Prog:** Δ=3.04 [0.95, 5.13], p=0.001*
  - **AD > MCI-Stable:** Δ=4.83 [3.14, 6.51], p<0.001*
  - **AD > NC:** Δ=5.33 [3.41, 7.24], p<0.001*
  - **MCI-Prog > NC:** Δ=2.28 [0.01, 4.56], p=0.049*
  - MCI-Prog vs MCI-Stable: Δ=1.78 [−0.30, 3.87], p=0.124
  - MCI-Stable vs NC: Δ=0.50 [−1.41, 2.41], p=0.907
- **CT**
  - **AD > MCI-Prog:** Δ=11.24 [6.90, 15.59], p<0.001*
  - **AD > MCI-Stable:** Δ=13.53 [10.04, 17.02], p<0.001*
  - **AD > NC:** Δ=15.28 [11.31, 19.26], p<0.001*
  - MCI-Prog vs MCI-Stable: Δ=2.29 [−2.04, 6.61], p=0.526
  - MCI-Prog vs NC: Δ=4.04 [−0.69, 8.77], p=0.124
  - MCI-Stable vs NC: Δ=1.76 [−2.20, 5.71], p=0.665
- **GM**
  - **AD > MCI-Prog:** Δ=4.79 [2.59, 6.98], p<0.001*
  - **AD > MCI-Stable:** Δ=6.46 [4.70, 8.22], p<0.001*
  - **AD > NC:** Δ=6.81 [4.80, 8.82], p<0.001*
  - MCI-Prog vs MCI-Stable: Δ=1.68 [−0.51, 3.86], p=0.200
  - MCI-Prog vs NC: Δ=2.03 [−0.36, 4.42], p=0.129
  - MCI-Stable vs NC: Δ=0.35 [−1.65, 2.35], p=0.969

Taken together, **only MIND detects an early separation between MCI-Progressive and controls**, while all three metrics robustly separate AD from the other groups. This suggests that the contribution of normative modelling of MIND over the other metrics is that it adds sensitivity for **pre-dementia clinical stratification** without sacrificing overall disease-related discrimination.

**3.3 Spatial Differences**
Visual comparison of deviation overlap patterns revealed distinct spatial signatures across metrics (**Supplementary Figure 11**). The metrics showed different deviation distributions both from the MIND ones shown in Figure 1 and between the two univariate metrics explored, with CT showing wider negative burden and GM showing more distributed posterior patterns in AD. Both CT and GM did not show many areas of increased deviation burden (significant positive deviations after FDR correction). These distinct spatial signatures suggest these metrics capture partially non-overlapping aspects of brain pathology.


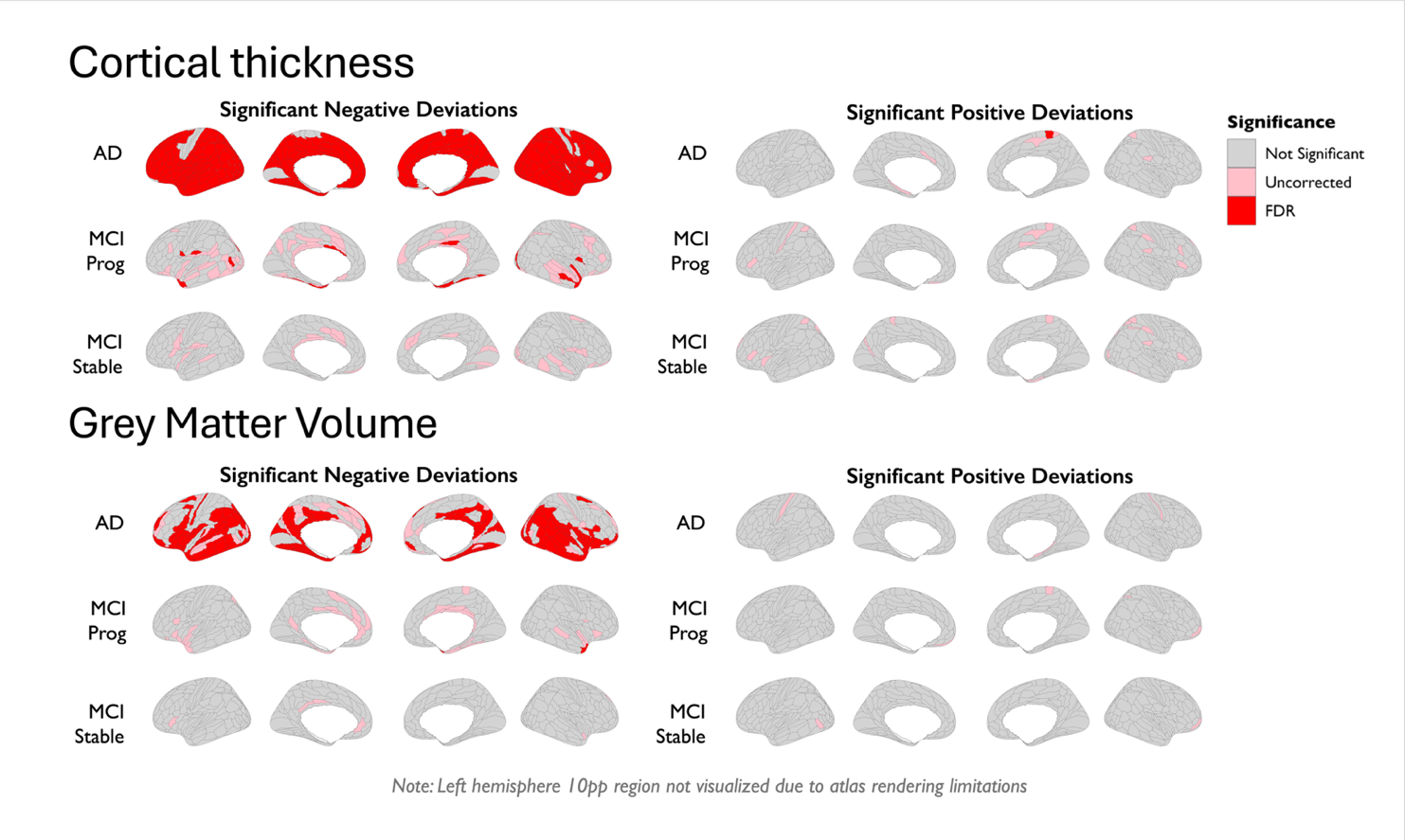


**Supplementary Figure 9-** **Group-level cortical deviations maps for cortical thickness and grey matter volume.** Left: Cortical regions with significantly higher negative deviation overlap relative to controls, based on permutation testing (N= 10,000). Right: Cortical regions with significantly higher positive deviation overlap relative to controls, based on permutation testing (N= 10,000). In all panels, red indicates regions significant after FDR correction, and pink shows uncorrected significance (p < 0.05). NC = normal controls (test set); MCI = mild cognitive impairment.

**3.4 Neuropathology analyses**
Lastly, we also compared the metrics with respect to prognostic value for predicting neuropathology changes in the subset of people with postmortem data (demographic and clinical characteristics of this subset are summarized in Supplementary Table 7). We repeated the same ordinal logistic regression analyses reported in the main text, but using CT and GM deviation counts (both positive and negative). We first used matched model structures to those used initially for MIND to allow for direct comparison. Additionally, we also conducted exploratory analyses allowing each metric to independently select its optimal model via Akaike Information Criterion (AIC).

**Matched Model Comparison**Using the age-interaction model structure (optimal for MIND, AIC=427.8), we found that only MIND maintained a significant association between number of negative deviations and ABC scores (β=0.67, SE=0.31, p=0.032). Both GM (β=-0.21, SE=0.24, p=0.384) and CT (β=-0.03, SE=0.08, p=0.670) showed non-significant associations under this model specification.

**Metric-Optimised Models**When allowing each metric to select its optimal model via AIC, different patterns emerged. Both GM (AIC=423.6) and CT (AIC=417.2) achieved better fit with time-to-death interaction models, with significant main effect of negative deviation count (GM: β=0.38, p=0.007; CT: β=0.18, p=0.004) and significant interaction between number of negative deviations and time-to-death (GM: β=-0.033, p=0.012; CT: β=-0.015, p=0.005).

These similar associations likely reflect that all three metrics capture variance associated with aggregate neuropathological changes, although MIND captures more robust age-related pathology signals, whereas univariate metrics do not.

**7.5 Genetic analyses**

MIND has previously demonstrated strong correspondence with brain transcriptomic co-expression networks and higher heritability than univariate morphometric measures (Sebenius et al., 2023), suggesting enhanced sensitivity to genetic variation. We therefore directly assessed whether MIND's multivariate integration provides meaningful advantages over using CT or GM for detecting gene-brain relationships relevant to precision medicine.

The same ordinary least squares (OLS) regressions were run to evaluate the effect of APOE genotype on the number of extreme **positive** and **negative** deviations, using ε3 homozygotes as the reference group and controlling for age and sex. For both outcomes, we also examined interaction terms with age and sex. Our primary analysis used matched model structures to those used originally for MIND (and detailed in the main manuscript Section 3.4). This allowed for direct comparison. Additionally, we also conducted exploratory analyses allowing each metric to independently select its optimal model via Akaike Information Criterion (AIC). This approach tests whether alternative model specifications might reveal genetic effects in CT or GM.

**Positive deviations**
For positive deviations, the best model across all three metrics (MIND, CT, and GM) was the model without interactions (main effects only – AIC for MIND= 11130, AIC for CT = 10419, and AIC for GM=10619). We already knew from our prior analysis that MIND showed a ε2 carrier effect (β=4.76, p=0.016), potentially reflecting preserved brain structure in this protective genotype. When looking at CT and GM:

- For CT, APOE effects for ε2 carriers (vs ε3 homozygotes) were non-significant (β= -2.08 (SE= 1.50), 95%CI=[ -5.02, 0.86], p=0.16), same for ε4 heterozygotes (β=-0.82 (SE=0.81), 95%CI=[ -2.42, 0.77], p=0.31) and ε4 homozygotes (β= -2.11 (SE= 1.30), 95%CI=[-4.65, 0.44], p=0.10).
- For GM, APOE effects for ε2 carriers (vs ε3 homozygotes) were also non-significant (β= 0.61 (SE= 1.62), 95%CI=[ -2.57, 3.78], p=0.71), same for ε4 homozygotes (β= 1.02 (SE= 1.40), 95%CI=[ -1.73, 3.76], p=0.47). However, there was a significant effect with ε4 heterozygotes having fewer positive deviations than ε3 homozygotes (β= -1.96 (SE= 0.88), 95%CI=[ -3.68, -0.23], p=0.03).

These patterns indicate that positive-deviation biology differs across metrics and may capture distinct aspects of brain preservation or compensation mechanisms, with MIND uniquely capturing an ε2-related signal in this cohort.

**Negative** **deviations**

**a) Matched Model Comparison**
Following model selection in MIND that identified sex-specific APOE effects (detailed in main text Section 3.4), we applied identical sex interaction models (negative_deviation_count ~ APOE_group × sex + age) across all three metrics to enable fair comparison (Supplementary Table 11). All three metrics showed evidence of sex-specific ε4 vulnerability, but with different statistical robustness forming a clear hierarchy:

- MIND (highest sensitivity): Highly robust detection with significant overall model fit (F=5.45, p<0.001). Female ε4 homozygotes showed substantially elevated negative deviation counts compared to ε3 homozygotes (β=7.48, SE=1.86, p<0.001, 95%CI=[3.84, 11.13]), representing approximately 7-8 additional regions with z-scores below -1.96. This effect was absent in males (β=0.44, SE=1.76, p=0.80, 95%CI=[-3.02, 3.90]), with significant sex × ε4 interaction (p=0.006). – this is the same analysis presented in the main text
- Grey matter volume (good moderate sensitivity): The same biological signal was detected with moderate statistical power (overall model: F=2.23, p=0.023). Female ε4 homozygotes showed elevated negative deviations (β=5.46, SE=1.82, p=0.003, 95%CI=[1.90, 9.02]), an effect absent in males (β=-0.70, SE=1.72, p=0.69, 95%CI=[-4.07, 2.67]), with significant sex interaction (p=0.014). Effect magnitude was reduced compared to MIND (~5-6 vs ~7-8 regions).
- Cortical thickness (low sensitivity): Evidence of the same pattern int term of effect but with low statistical robustness and no statistical significance. Despite similar effect size for the ε4 homozygotes female effect size (β=7.52, SE=3.86, p=0.052, 95%CI=[-0.06, 15.10]), both this effect (p=0.052) and the overall model did not reach significance (F=1.27, p=0.254). Same for the ε4 male effect: β=-2.99, SE=5.00, p=0.42, 95%CI=[-12.80, 6.82]. This pattern of similar coefficient magnitude but substantially reduced statistical power, suggests CT captures similar biological signal but with insufficient sensitivity for reliable detection.

**Supplementary Table 1** **- Sex-Specific APOE4 Homozygote Effects: Matched Model Comparison.** Effects represent additional deviant regions (z<-1.96) in APOE4 homozygotes vs APOE3 homozygotes. Standard errors in parentheses. Similar coefficient magnitudes indicate all metrics detect the same biological signal, but with differences in statistical confidence. ***p<0.001, **p<0.01, *p<0.05

| **Metric** | **Overall Model** | **Female e4 effect** | **Male e4 effect** | **Sex interaction** |
| --- | --- | --- | --- | --- |
| **MIND** | F=5.45, p<0.001*** | β=7.48 (1.86), p<0.001*** | β=0.44 (1.76), p=0.80 | p=0.006** |
| **GM** | F=2.23, p=0.023* | β=5.46 (1.82), p=0.003** | β=-0.70 (1.72), p=0.69 | p=0.014* |
| **CT** | F=1.27, p=0.254 | β=7.52 (3.86), p=0.052 | β=-2.99 (5.00), p=0.42 | p=0.048* |

These findings reveal a hierarchy of genetic sensitivity (MIND > GM > CT) with potential implications for applications in precision medicine. The similar coefficient magnitudes across metrics (MIND β=7.48, CT β=7.52, GM β=5.46) validate that all three detect the same underlying biological phenomenon of female-specific APOE4 vulnerability, but with different signal-to-noise ratios. MIND's superior signal extraction provides statistical robustness required for clinical risk stratification, whereas CT's marginal detection (p≈0.052) would be unsuitable for actionable predictions. This represents a higher advantage in statistical confidence (p<0.001 vs p=0.003 vs p=0.052).

**b) Metric-Optimised Models**

To test whether alternative model specifications might reveal clearer genetic effects in CT or GM, we also conducted exploratory analyses allowing each metric to independently select its optimal model via Akaike Information Criterion (AIC).

- For CT negative deviations, a full 3-way interaction model (APOE × age × sex) provided better fit (AIC=12,290.5). However, parameter estimates were highly unstable (e.g., e4 homozygotes: β=65.9, SE=34.3, 95%CI=[-1.5, 133.2], p=0.055), with severe multicollinearity (condition number=7,360).
- For GM negative deviations, also the full interaction model was AIC-optimal (AIC=10,329.6 vs 10,334.6 for basic model, ΔAIC=5.0), but similarly showed high collinearity and unstable parameters.

These secondary analyses reinforce that CT and GM have lower sensitivity than MIND to detect genetic associations reliably when allowed to explore complex model structures, as the results could suggest overfitting, particularly given smaller sample sizes in some of the APOE × sex × age strata. MIND's consistent detection of sex-specific ε4 homozygotes effects across both matched and AIC-selected approaches demonstrates it to be a more robust metric for this specific analysis.

## **Group ANOVA For Positive and Negative Deviation Counts**

Supplementary Table 1 provides detailed statistical outputs for group comparisons of deviation counts. One-way ANOVAs were performed separately for positive and negative deviations, followed by Tukey’s HSD post-hoc tests to assess pairwise group differences. Positive and negative deviation counts were calculated as the number of cortical regions with Z-scores greater than 1.96 or less than -1.96, respectively. The analysis included all cortical regions assessed using the MIND normative model. Full results, including confidence intervals and adjusted p-values, are shown in Supplementary Tables 2 and 3 below.

**Supplementary Table 2**- ANOVA and Tukey HSD post-hoc test results for positive and negative deviation counts across diagnostic groups.

| **Deviation Type** | **Comparison** | **Mean Diff** | **Lower CI** | **Upper CI** | **p-adj** | **Significant** | **ANOVA F** | **ANOVA p-value** |
| --- | --- | --- | --- | --- | --- | --- | --- | --- |
| **Positive** | AD vs. MCI Progressive | -0.42 | -3.36 | 2.53 | 0.98 | No | 0.59 | 0.62 |
|  | AD vs. MCI Stable | -1.11 | -3.47 | 1.26 | 0.62 | No |  |  |
|  | AD vs. NC | -1.03 | -3.73 | 1.66 | 0.76 | No |  |  |
|  | MCI Progressive vs. MCI Stable | -0.69 | -3.62 | 2.24 | 0.93 | No |  |  |
|  | MCI Progressive vs. NC | -0.62 | -3.82 | 2.59 | 0.96 | No |  |  |
|  | MCI Stable vs. NC | 0.07 | -2.61 | 2.75 | 1.00 | No |  |  |
| **Negative** | AD vs. MCI Progressive | -3.04 | -5.13 | -0.95 | 0.00 | Yes | 24.39 | <0.001 |
|  | AD vs. MCI Stable | -4.83 | -6.51 | -3.14 | <0.001 | Yes |  |  |
|  | AD vs. NC | -5.33 | -7.24 | -3.41 | <0.001 | Yes |  |  |
|  | MCI Progressive vs. MCI Stable | -1.78 | -3.87 | 0.30 | 0.12 | No |  |  |
|  | MCI Progressive vs. NC | -2.28 | -4.56 | 0.00 | 0.05 | Yes |  |  |
|  | MCI Stable vs. NC | -0.50 | -2.41 | 1.41 | 0.91 | No |  |  |

**Supplementary Table 3 –** Full details and p-values of the Cohen’s d analyses with FDR correction.

| **ROI  (lh, rh  hemi)** | **Comparison** | **Less  Severe Group** | **More  Severe  Group** | **Mean  Less  Severe** | **Mean  More  Severe** | **Cohens d observed** | **P FDR** |
| --- | --- | --- | --- | --- | --- | --- | --- |
| lhV1 | MCI Progressive_vs_AD | MCI Progressive | AD | -0.099 | 0.261 | -0.335 | 0.0090 |
| rhV1 | MCI Progressive_vs_AD | MCI Progressive | AD | -0.140 | 0.206 | -0.324 | 0.0090 |
| rh3b | MCI Progressive_vs_AD | MCI Progressive | AD | 0.116 | 0.427 | -0.309 | 0.0090 |
| lh3b | MCI Progressive_vs_AD | MCI Progressive | AD | 0.155 | 0.436 | -0.277 | 0.0120 |
| lh23c | MCI Progressive_vs_AD | MCI Progressive | AD | 0.224 | -0.027 | 0.241 | 0.0432 |
| rhd32 | MCI Progressive_vs_AD | MCI Progressive | AD | 0.279 | 0.017 | 0.248 | 0.0320 |
| lh24dv | MCI Progressive_vs_AD | MCI Progressive | AD | 0.151 | -0.094 | 0.249 | 0.0315 |
| lh47s | MCI Progressive_vs_AD | MCI Progressive | AD | 0.233 | -0.030 | 0.258 | 0.0309 |
| rhFOP2 | MCI Progressive_vs_AD | MCI Progressive | AD | 0.176 | -0.086 | 0.262 | 0.0120 |
| lhFOP3 | MCI Progressive_vs_AD | MCI Progressive | AD | 0.222 | -0.075 | 0.303 | 0.0090 |
| lh24dv | MCI Stable_vs_AD | MCI Stable | AD | 0.317 | -0.094 | 0.434 | 0.0006 |
| lhp32pr | MCI Stable_vs_AD | MCI Stable | AD | 0.295 | -0.073 | 0.387 | 0.0006 |
| lhFOP3 | MCI Stable_vs_AD | MCI Stable | AD | 0.281 | -0.075 | 0.366 | 0.0006 |
| lh23c | MCI Stable_vs_AD | MCI Stable | AD | 0.337 | -0.027 | 0.362 | 0.0006 |
| rhFOP2 | MCI Stable_vs_AD | MCI Stable | AD | 0.264 | -0.086 | 0.361 | 0.0006 |
| rha32pr | MCI Stable_vs_AD | MCI Stable | AD | 0.362 | 0.030 | 0.346 | 0.0006 |
| rhOP2-3 | MCI Stable_vs_AD | MCI Stable | AD | 0.210 | -0.117 | 0.338 | 0.0006 |
| lhFOP2 | MCI Stable_vs_AD | MCI Stable | AD | 0.277 | -0.033 | 0.321 | 0.0006 |
| rh24dv | MCI Stable_vs_AD | MCI Stable | AD | 0.226 | -0.085 | 0.317 | 0.0006 |
| lhp24 | MCI Stable_vs_AD | MCI Stable | AD | 0.360 | 0.059 | 0.316 | 0.0006 |
| lhPHA2 | MCI Stable_vs_AD | MCI Stable | AD | 0.244 | -0.059 | 0.314 | 0.0006 |
| rhp32 | MCI Stable_vs_AD | MCI Stable | AD | 0.280 | -0.018 | 0.311 | 0.0006 |
| lh31pd | MCI Stable_vs_AD | MCI Stable | AD | 0.239 | -0.047 | 0.307 | 0.0006 |
| rhPOS2 | MCI Stable_vs_AD | MCI Stable | AD | 0.265 | -0.033 | 0.302 | 0.0006 |
| rhd32 | MCI Stable_vs_AD | MCI Stable | AD | 0.322 | 0.017 | 0.299 | 0.0006 |
| lhPOS2 | MCI Stable_vs_AD | MCI Stable | AD | 0.235 | -0.054 | 0.298 | 0.0006 |
| rhPGi | MCI Stable_vs_AD | MCI Stable | AD | 0.204 | -0.067 | 0.294 | 0.0006 |
| rhFOP3 | MCI Stable_vs_AD | MCI Stable | AD | 0.222 | -0.055 | 0.289 | 0.0006 |
| lha24 | MCI Stable_vs_AD | MCI Stable | AD | 0.314 | 0.035 | 0.285 | 0.0006 |
| lhd32 | MCI Stable_vs_AD | MCI Stable | AD | 0.179 | -0.103 | 0.283 | 0.0006 |
| lha24pr | MCI Stable_vs_AD | MCI Stable | AD | 0.301 | 0.040 | 0.279 | 0.0010 |
| rhIP2 | MCI Stable_vs_AD | MCI Stable | AD | 0.119 | -0.147 | 0.275 | 0.0006 |
| lhFOP4 | MCI Stable_vs_AD | MCI Stable | AD | 0.241 | -0.021 | 0.273 | 0.0006 |
| rhIP0 | MCI Stable_vs_AD | MCI Stable | AD | 0.150 | -0.107 | 0.271 | 0.0006 |
| lhp24pr | MCI Stable_vs_AD | MCI Stable | AD | 0.259 | 0.003 | 0.271 | 0.0006 |
| rhPHA2 | MCI Stable_vs_AD | MCI Stable | AD | 0.267 | 0.011 | 0.271 | 0.0006 |
| rh31pd | MCI Stable_vs_AD | MCI Stable | AD | 0.183 | -0.075 | 0.271 | 0.0006 |
| lh6a | MCI Stable_vs_AD | MCI Stable | AD | 0.202 | -0.048 | 0.267 | 0.0006 |
| rhp32pr | MCI Stable_vs_AD | MCI Stable | AD | 0.247 | -0.011 | 0.267 | 0.0006 |
| rha24 | MCI Stable_vs_AD | MCI Stable | AD | 0.373 | 0.108 | 0.265 | 0.0006 |
| rh23c | MCI Stable_vs_AD | MCI Stable | AD | 0.309 | 0.041 | 0.264 | 0.0006 |
| rhAVI | MCI Stable_vs_AD | MCI Stable | AD | 0.154 | -0.098 | 0.262 | 0.0006 |
| lh24dd | MCI Stable_vs_AD | MCI Stable | AD | 0.173 | -0.078 | 0.258 | 0.0006 |
| lh47s | MCI Stable_vs_AD | MCI Stable | AD | 0.223 | -0.030 | 0.257 | 0.0006 |
| lh31a | MCI Stable_vs_AD | MCI Stable | AD | 0.255 | 0.008 | 0.254 | 0.0006 |
| rh24dd | MCI Stable_vs_AD | MCI Stable | AD | 0.230 | -0.011 | 0.249 | 0.0006 |
| rhp24pr | MCI Stable_vs_AD | MCI Stable | AD | 0.270 | 0.027 | 0.248 | 0.0006 |
| lhAVI | MCI Stable_vs_AD | MCI Stable | AD | 0.120 | -0.128 | 0.248 | 0.0006 |
| lhPFm | MCI Stable_vs_AD | MCI Stable | AD | 0.128 | -0.123 | 0.248 | 0.0006 |
| lhPI | MCI Stable_vs_AD | MCI Stable | AD | 0.239 | 0.004 | 0.246 | 0.0006 |
| lha32pr | MCI Stable_vs_AD | MCI Stable | AD | 0.186 | -0.058 | 0.242 | 0.0006 |
| rh9p | MCI Stable_vs_AD | MCI Stable | AD | 0.185 | -0.049 | 0.241 | 0.0006 |
| lhV3CD | MCI Stable_vs_AD | MCI Stable | AD | 0.135 | -0.104 | 0.241 | 0.0006 |
| rhPGs | MCI Stable_vs_AD | MCI Stable | AD | 0.126 | -0.117 | 0.241 | 0.0006 |
| rhPFm | MCI Stable_vs_AD | MCI Stable | AD | 0.189 | -0.043 | 0.239 | 0.0006 |
| lhp32 | MCI Stable_vs_AD | MCI Stable | AD | 0.214 | -0.017 | 0.239 | 0.0006 |
| rhSCEF | MCI Stable_vs_AD | MCI Stable | AD | 0.106 | -0.118 | 0.235 | 0.0010 |
| lhIP0 | MCI Stable_vs_AD | MCI Stable | AD | 0.108 | -0.119 | 0.234 | 0.0006 |
| lhTPOJ2 | MCI Stable_vs_AD | MCI Stable | AD | 0.177 | -0.050 | 0.229 | 0.0006 |
| rhTPOJ2 | MCI Stable_vs_AD | MCI Stable | AD | 0.217 | -0.002 | 0.228 | 0.0010 |
| rh47l | MCI Stable_vs_AD | MCI Stable | AD | 0.224 | 0.018 | 0.224 | 0.0010 |
| rh31a | MCI Stable_vs_AD | MCI Stable | AD | 0.253 | 0.031 | 0.223 | 0.0013 |
| rh9m | MCI Stable_vs_AD | MCI Stable | AD | 0.187 | -0.030 | 0.221 | 0.0017 |
| rhIPS1 | MCI Stable_vs_AD | MCI Stable | AD | 0.200 | -0.016 | 0.221 | 0.0006 |
| rh10v | MCI Stable_vs_AD | MCI Stable | AD | 0.239 | 0.026 | 0.219 | 0.0013 |
| rhPI | MCI Stable_vs_AD | MCI Stable | AD | 0.250 | 0.057 | 0.215 | 0.0010 |
| rh25 | MCI Stable_vs_AD | MCI Stable | AD | 0.219 | 0.004 | 0.214 | 0.0010 |
| lh52 | MCI Stable_vs_AD | MCI Stable | AD | 0.197 | -0.026 | 0.212 | 0.0013 |
| lhIP1 | MCI Stable_vs_AD | MCI Stable | AD | 0.136 | -0.070 | 0.210 | 0.0010 |
| lhV4 | MCI Stable_vs_AD | MCI Stable | AD | 0.095 | -0.119 | 0.209 | 0.0010 |
| lhs32 | MCI Stable_vs_AD | MCI Stable | AD | 0.143 | -0.058 | 0.208 | 0.0020 |
| rhVMV3 | MCI Stable_vs_AD | MCI Stable | AD | 0.161 | -0.038 | 0.208 | 0.0017 |
| lhOP2-3 | MCI Stable_vs_AD | MCI Stable | AD | 0.142 | -0.049 | 0.205 | 0.0013 |
| rh52 | MCI Stable_vs_AD | MCI Stable | AD | 0.247 | 0.045 | 0.205 | 0.0010 |
| rhFOP5 | MCI Stable_vs_AD | MCI Stable | AD | 0.155 | -0.045 | 0.205 | 0.0023 |
| rhPH | MCI Stable_vs_AD | MCI Stable | AD | 0.212 | 0.015 | 0.204 | 0.0017 |
| lhLO2 | MCI Stable_vs_AD | MCI Stable | AD | 0.037 | -0.164 | 0.204 | 0.0017 |
| rha24pr | MCI Stable_vs_AD | MCI Stable | AD | 0.287 | 0.102 | 0.203 | 0.0020 |
| lhTPOJ1 | MCI Stable_vs_AD | MCI Stable | AD | 0.141 | -0.052 | 0.203 | 0.0023 |
| lh10v | MCI Stable_vs_AD | MCI Stable | AD | 0.229 | 0.026 | 0.203 | 0.0010 |
| lh23d | MCI Stable_vs_AD | MCI Stable | AD | 0.217 | 0.016 | 0.202 | 0.0039 |
| rhMT | MCI Stable_vs_AD | MCI Stable | AD | 0.188 | -0.007 | 0.201 | 0.0020 |
| rhPFt | MCI Stable_vs_AD | MCI Stable | AD | 0.165 | -0.023 | 0.200 | 0.0013 |
| lhFOP5 | MCI Stable_vs_AD | MCI Stable | AD | 0.138 | -0.058 | 0.200 | 0.0020 |
| lhPGs | MCI Stable_vs_AD | MCI Stable | AD | 0.091 | -0.102 | 0.199 | 0.0010 |
| rhMI | MCI Stable_vs_AD | MCI Stable | AD | 0.153 | -0.043 | 0.198 | 0.0023 |
| lhPGi | MCI Stable_vs_AD | MCI Stable | AD | 0.184 | 0.001 | 0.197 | 0.0010 |
| lhOP1 | MCI Stable_vs_AD | MCI Stable | AD | 0.184 | 0.000 | 0.193 | 0.0030 |
| lhPFt | MCI Stable_vs_AD | MCI Stable | AD | 0.185 | 0.000 | 0.192 | 0.0042 |
| rhp24 | MCI Stable_vs_AD | MCI Stable | AD | 0.240 | 0.051 | 0.190 | 0.0036 |
| lhIP2 | MCI Stable_vs_AD | MCI Stable | AD | 0.116 | -0.073 | 0.190 | 0.0033 |
| rh47s | MCI Stable_vs_AD | MCI Stable | AD | 0.171 | -0.013 | 0.188 | 0.0052 |
| lh8Ad | MCI Stable_vs_AD | MCI Stable | AD | 0.205 | 0.024 | 0.186 | 0.0030 |
| lhPH | MCI Stable_vs_AD | MCI Stable | AD | 0.220 | 0.030 | 0.186 | 0.0033 |
| rhFOP4 | MCI Stable_vs_AD | MCI Stable | AD | 0.237 | 0.054 | 0.184 | 0.0054 |
| rhV3CD | MCI Stable_vs_AD | MCI Stable | AD | 0.112 | -0.076 | 0.184 | 0.0042 |
| rhPCV | MCI Stable_vs_AD | MCI Stable | AD | 0.188 | 0.009 | 0.183 | 0.0045 |
| lhPHA3 | MCI Stable_vs_AD | MCI Stable | AD | 0.231 | 0.047 | 0.182 | 0.0033 |
| lh13l | MCI Stable_vs_AD | MCI Stable | AD | 0.189 | 0.018 | 0.182 | 0.0050 |
| lhFOP1 | MCI Stable_vs_AD | MCI Stable | AD | 0.111 | -0.060 | 0.180 | 0.0050 |
| lhV8 | MCI Stable_vs_AD | MCI Stable | AD | 0.144 | -0.032 | 0.180 | 0.0056 |
| rh6r | MCI Stable_vs_AD | MCI Stable | AD | 0.177 | 0.003 | 0.180 | 0.0052 |
| lh9a | MCI Stable_vs_AD | MCI Stable | AD | 0.142 | -0.032 | 0.180 | 0.0050 |
| rhOP4 | MCI Stable_vs_AD | MCI Stable | AD | 0.158 | -0.021 | 0.179 | 0.0056 |
| rhLIPd | MCI Stable_vs_AD | MCI Stable | AD | 0.093 | -0.079 | 0.179 | 0.0054 |
| lh25 | MCI Stable_vs_AD | MCI Stable | AD | 0.164 | -0.007 | 0.178 | 0.0048 |
| rh6ma | MCI Stable_vs_AD | MCI Stable | AD | 0.108 | -0.068 | 0.178 | 0.0071 |
| lh10d | MCI Stable_vs_AD | MCI Stable | AD | 0.138 | -0.039 | 0.178 | 0.0045 |
| rhV4t | MCI Stable_vs_AD | MCI Stable | AD | 0.124 | -0.047 | 0.178 | 0.0065 |
| rhPSL | MCI Stable_vs_AD | MCI Stable | AD | 0.132 | -0.038 | 0.178 | 0.0069 |
| lhIg | MCI Stable_vs_AD | MCI Stable | AD | 0.140 | -0.027 | 0.175 | 0.0069 |
| rhSTSvp | MCI Stable_vs_AD | MCI Stable | AD | 0.194 | 0.031 | 0.175 | 0.0071 |
| rhPFcm | MCI Stable_vs_AD | MCI Stable | AD | 0.132 | -0.040 | 0.175 | 0.0076 |
| rhSTV | MCI Stable_vs_AD | MCI Stable | AD | 0.111 | -0.058 | 0.174 | 0.0068 |
| lhSCEF | MCI Stable_vs_AD | MCI Stable | AD | 0.115 | -0.053 | 0.174 | 0.0054 |
| rh6a | MCI Stable_vs_AD | MCI Stable | AD | 0.199 | 0.029 | 0.174 | 0.0069 |
| rhs32 | MCI Stable_vs_AD | MCI Stable | AD | 0.197 | 0.028 | 0.173 | 0.0061 |
| lh45 | MCI Stable_vs_AD | MCI Stable | AD | 0.214 | 0.054 | 0.172 | 0.0073 |
| lh7Pm | MCI Stable_vs_AD | MCI Stable | AD | 0.087 | -0.081 | 0.171 | 0.0076 |
| lhFST | MCI Stable_vs_AD | MCI Stable | AD | 0.123 | -0.045 | 0.170 | 0.0063 |
| rhLO2 | MCI Stable_vs_AD | MCI Stable | AD | 0.106 | -0.067 | 0.170 | 0.0087 |
| rhMBelt | MCI Stable_vs_AD | MCI Stable | AD | 0.165 | 0.003 | 0.170 | 0.0073 |
| rhLO1 | MCI Stable_vs_AD | MCI Stable | AD | 0.073 | -0.095 | 0.163 | 0.0104 |
| lh9-46d | MCI Stable_vs_AD | MCI Stable | AD | 0.165 | 0.005 | 0.163 | 0.0106 |
| rhIg | MCI Stable_vs_AD | MCI Stable | AD | 0.145 | -0.011 | 0.163 | 0.0104 |
| rh7m | MCI Stable_vs_AD | MCI Stable | AD | 0.154 | -0.007 | 0.161 | 0.0134 |
| rh9-46d | MCI Stable_vs_AD | MCI Stable | AD | 0.096 | -0.059 | 0.160 | 0.0123 |
| rhOP1 | MCI Stable_vs_AD | MCI Stable | AD | 0.127 | -0.029 | 0.158 | 0.0150 |
| lhv23ab | MCI Stable_vs_AD | MCI Stable | AD | 0.105 | -0.049 | 0.156 | 0.0170 |
| lhVMV3 | MCI Stable_vs_AD | MCI Stable | AD | 0.150 | -0.001 | 0.156 | 0.0150 |
| lhSTSva | MCI Stable_vs_AD | MCI Stable | AD | 0.119 | -0.035 | 0.156 | 0.0159 |
| lhRSC | MCI Stable_vs_AD | MCI Stable | AD | 0.103 | -0.056 | 0.155 | 0.0158 |
| rh47m | MCI Stable_vs_AD | MCI Stable | AD | 0.127 | -0.022 | 0.154 | 0.0134 |
| rhLBelt | MCI Stable_vs_AD | MCI Stable | AD | 0.165 | 0.012 | 0.154 | 0.0154 |
| rh23d | MCI Stable_vs_AD | MCI Stable | AD | 0.143 | -0.010 | 0.154 | 0.0134 |
| lhMST | MCI Stable_vs_AD | MCI Stable | AD | 0.061 | -0.093 | 0.154 | 0.0161 |
| rhs6-8 | MCI Stable_vs_AD | MCI Stable | AD | 0.133 | -0.019 | 0.154 | 0.0143 |
| lhA5 | MCI Stable_vs_AD | MCI Stable | AD | 0.168 | 0.020 | 0.152 | 0.0172 |
| lhLO3 | MCI Stable_vs_AD | MCI Stable | AD | 0.063 | -0.093 | 0.151 | 0.0214 |
| lhPoI1 | MCI Stable_vs_AD | MCI Stable | AD | 0.203 | 0.049 | 0.149 | 0.0169 |
| rhV4 | MCI Stable_vs_AD | MCI Stable | AD | 0.129 | -0.023 | 0.147 | 0.0222 |
| rhPGp | MCI Stable_vs_AD | MCI Stable | AD | 0.042 | -0.109 | 0.145 | 0.0223 |
| lhPIT | MCI Stable_vs_AD | MCI Stable | AD | 0.107 | -0.036 | 0.145 | 0.0226 |
| lhPFcm | MCI Stable_vs_AD | MCI Stable | AD | 0.164 | 0.021 | 0.145 | 0.0232 |
| rh8C | MCI Stable_vs_AD | MCI Stable | AD | 0.205 | 0.060 | 0.144 | 0.0272 |
| rh6mp | MCI Stable_vs_AD | MCI Stable | AD | 0.193 | 0.049 | 0.143 | 0.0293 |
| rhPIT | MCI Stable_vs_AD | MCI Stable | AD | 0.154 | 0.010 | 0.143 | 0.0248 |
| lhMI | MCI Stable_vs_AD | MCI Stable | AD | 0.116 | -0.023 | 0.142 | 0.0296 |
| rh9a | MCI Stable_vs_AD | MCI Stable | AD | 0.110 | -0.023 | 0.141 | 0.0263 |
| lhV4t | MCI Stable_vs_AD | MCI Stable | AD | 0.078 | -0.057 | 0.140 | 0.0312 |
| lhMBelt | MCI Stable_vs_AD | MCI Stable | AD | 0.173 | 0.040 | 0.139 | 0.0312 |
| lhIPS1 | MCI Stable_vs_AD | MCI Stable | AD | 0.121 | -0.016 | 0.139 | 0.0307 |
| lhOP4 | MCI Stable_vs_AD | MCI Stable | AD | 0.143 | 0.010 | 0.139 | 0.0339 |
| rhV8 | MCI Stable_vs_AD | MCI Stable | AD | 0.115 | -0.024 | 0.139 | 0.0337 |
| rhFFC | MCI Stable_vs_AD | MCI Stable | AD | 0.151 | 0.017 | 0.139 | 0.0296 |
| lhPCV | MCI Stable_vs_AD | MCI Stable | AD | 0.177 | 0.046 | 0.137 | 0.0356 |
| lh7m | MCI Stable_vs_AD | MCI Stable | AD | 0.138 | 0.008 | 0.136 | 0.0339 |
| lh31pv | MCI Stable_vs_AD | MCI Stable | AD | 0.221 | 0.091 | 0.135 | 0.0334 |
| rhd23ab | MCI Stable_vs_AD | MCI Stable | AD | 0.085 | -0.048 | 0.135 | 0.0339 |
| rhp10p | MCI Stable_vs_AD | MCI Stable | AD | 0.094 | -0.037 | 0.134 | 0.0339 |
| rhRSC | MCI Stable_vs_AD | MCI Stable | AD | 0.072 | -0.061 | 0.132 | 0.0393 |
| lhPBelt | MCI Stable_vs_AD | MCI Stable | AD | 0.192 | 0.068 | 0.131 | 0.0389 |
| rhV3B | MCI Stable_vs_AD | MCI Stable | AD | 0.124 | -0.006 | 0.131 | 0.0434 |
| lhMT | MCI Stable_vs_AD | MCI Stable | AD | 0.140 | 0.009 | 0.130 | 0.0408 |
| rhIFJa | MCI Stable_vs_AD | MCI Stable | AD | 0.102 | -0.026 | 0.130 | 0.0445 |
| rhFOP1 | MCI Stable_vs_AD | MCI Stable | AD | 0.087 | -0.037 | 0.129 | 0.0420 |
| lh44 | MCI Stable_vs_AD | MCI Stable | AD | 0.142 | 0.014 | 0.129 | 0.0481 |
| rhVMV1 | MCI Stable_vs_AD | MCI Stable | AD | 0.115 | -0.008 | 0.128 | 0.0479 |
| lhSTV | MCI Stable_vs_AD | MCI Stable | AD | 0.073 | -0.052 | 0.128 | 0.0484 |
| rhVIP | MCI Stable_vs_AD | MCI Stable | AD | 0.141 | 0.015 | 0.127 | 0.0483 |
| lha10p | MCI Stable_vs_AD | MCI Stable | AD | 0.083 | -0.041 | 0.127 | 0.0447 |
| rh45 | MCI Stable_vs_AD | MCI Stable | AD | 0.153 | 0.032 | 0.127 | 0.0466 |
| lhp9-46v | MCI Stable_vs_AD | MCI Stable | AD | 0.185 | 0.066 | 0.126 | 0.0481 |
| lhTF | MCI Stable_vs_AD | MCI Stable | AD | 0.103 | 0.232 | -0.128 | 0.0481 |
| rh2 | MCI Stable_vs_AD | MCI Stable | AD | 0.115 | 0.252 | -0.136 | 0.0334 |
| lhPeEc | MCI Stable_vs_AD | MCI Stable | AD | 0.041 | 0.183 | -0.139 | 0.0317 |
| lhTGv | MCI Stable_vs_AD | MCI Stable | AD | -0.004 | 0.146 | -0.148 | 0.0197 |
| lh3a | MCI Stable_vs_AD | MCI Stable | AD | 0.122 | 0.271 | -0.154 | 0.0150 |
| rh1 | MCI Stable_vs_AD | MCI Stable | AD | 0.156 | 0.324 | -0.165 | 0.0075 |
| rhPeEc | MCI Stable_vs_AD | MCI Stable | AD | -0.067 | 0.106 | -0.174 | 0.0061 |
| lh1 | MCI Stable_vs_AD | MCI Stable | AD | 0.175 | 0.353 | -0.181 | 0.0063 |
| lh5m | MCI Stable_vs_AD | MCI Stable | AD | 0.064 | 0.245 | -0.186 | 0.0030 |
| lhH | MCI Stable_vs_AD | MCI Stable | AD | -0.292 | 0.006 | -0.190 | 0.0013 |
| rhPreS | MCI Stable_vs_AD | MCI Stable | AD | -0.148 | 0.045 | -0.199 | 0.0020 |
| rhTGd | MCI Stable_vs_AD | MCI Stable | AD | -0.028 | 0.169 | -0.200 | 0.0017 |
| rhV6 | MCI Stable_vs_AD | MCI Stable | AD | 0.003 | 0.203 | -0.201 | 0.0013 |
| rh3a | MCI Stable_vs_AD | MCI Stable | AD | 0.137 | 0.359 | -0.212 | 0.0013 |
| lhPreS | MCI Stable_vs_AD | MCI Stable | AD | -0.144 | 0.093 | -0.240 | 0.0006 |
| lhTE1a | MCI Stable_vs_AD | MCI Stable | AD | 0.012 | 0.265 | -0.256 | 0.0010 |
| lhEC | MCI Stable_vs_AD | MCI Stable | AD | -0.088 | 0.174 | -0.257 | 0.0006 |
| rhH | MCI Stable_vs_AD | MCI Stable | AD | -0.232 | 0.109 | -0.260 | 0.0006 |
| rhEC | MCI Stable_vs_AD | MCI Stable | AD | -0.130 | 0.153 | -0.281 | 0.0006 |
| lhV2 | MCI Stable_vs_AD | MCI Stable | AD | -0.187 | 0.098 | -0.289 | 0.0006 |
| lhTGd | MCI Stable_vs_AD | MCI Stable | AD | -0.087 | 0.230 | -0.310 | 0.0006 |
| rhV2 | MCI Stable_vs_AD | MCI Stable | AD | -0.311 | 0.008 | -0.328 | 0.0006 |
| lhV1 | MCI Stable_vs_AD | MCI Stable | AD | -0.185 | 0.261 | -0.428 | 0.0006 |
| rh3b | MCI Stable_vs_AD | MCI Stable | AD | -0.024 | 0.427 | -0.443 | 0.0006 |
| lh3b | MCI Stable_vs_AD | MCI Stable | AD | -0.020 | 0.436 | -0.457 | 0.0006 |
| rhV1 | MCI Stable_vs_AD | MCI Stable | AD | -0.312 | 0.206 | -0.506 | 0.0006 |
| rhPeEc | MCI Stable_vs_MCI Progressive | MCI Stable | MCI Progressive | -0.067 | 0.194 | -0.258 | 0.0360 |
| rhs32 | MCI Stable_vs_MCI Progressive | MCI Stable | MCI Progressive | 0.197 | -0.039 | 0.252 | 0.0360 |
| rhIPS1 | MCI Stable_vs_MCI Progressive | MCI Stable | MCI Progressive | 0.200 | -0.073 | 0.276 | 0.0360 |
| lhIP0 | MCI Stable_vs_MCI Progressive | MCI Stable | MCI Progressive | 0.108 | -0.194 | 0.321 | 0.0360 |

## **APOE And Normative MIND Analyses**

We conducted ordinary least squares (OLS) regressions to evaluate the effect of APOE genotype on the number of extreme **positive** and **negative** deviations in regional MIND z-scores, using ε3 homozygotes as the reference group and controlling for age and sex. For both outcomes, we also examined interaction terms with age and sex.

The sex composition for the groups were as follows: ε3 homozygotes: N=289 females and N=267 males; ε2 carriers: N=39 females and N=54 males; ε4 heterozygotes: N=283 females and N=234 males; ε4 homozygotes: N=62 females and N=71 males. For the analyses, males were coded as 1 and females as 0.

Supplementary Table 4 shows the comparison of nested models for both positive and negative deviation counts. For positive deviations, the basic additive model provided the best fit (lowest AIC = 11,130.3), with no significant improvement from interaction terms. For negative deviations, the sex interaction model was the best (AIC = 10,397.7), showing significant improvement over the basic model (p = 0.040).

**Supplementary Table 4** – Summary of the comparison of nested APOE models for both positive and negative deviation counts

| **Outcome** | **Model** | **Log-Likelihood** | **AIC** |
| --- | --- | --- | --- |
| Positive Deviation Count | Basic | -5559.14 | 11130.28 |
| Positive Deviation Count | Age Interaction | -5557.69 | 11133.38 |
| Positive Deviation Count | Sex Interaction | -5557.56 | 11133.11 |
| Positive Deviation Count | Full Interaction | -5555.14 | 11142.27 |
| Negative Deviation Count | Basic | -5193.99 | 10399.99 |
| Negative Deviation Count | Age Interaction | -5192.6 | 10403.2 |
| Negative Deviation Count | Sex Interaction | -5189.85 | 10397.7 |
| Negative Deviation Count | Full Interaction | -5183.57 | 10399.13 |

Supplementary Table 5 below presents the full regression results for the best-fitting models. For positive deviations, only ε2 carriers showed significant differences from ε3 homozygotes (β = 4.76, p = 0.016). For negative deviations, the key finding was the significant APOE × sex interaction for ε4 homozygotes (β = -7.04, p = 0.006), indicating that the effect of ε4 homozygosity is largely confined to females.

**Supplementary Table 5 –** Full results from the best fitting models assessing APOE genotype effects on cortical deviation burden.

| **Outcome** | **Predictor** | **Coeff.** | **SE** | **t-stat** | **p-val** | **95% CI Lower** | **95% CI Upper** |
| --- | --- | --- | --- | --- | --- | --- | --- |
| Positive Deviation Count (Basic Model) | Intercept | 1.666 | 4.126 | 0.404 | 0.686 | -6.429 | 9.761 |
| Positive Deviation Count (Basic Model) | APOE: e2 carriers | 4.762 | 1.972 | 2.415 | 0.016 | 0.894 | 8.63 |
| Positive Deviation Count (Basic Model) | APOE: e4 heterozygotes | 0.739 | 1.071 | 0.69 | 0.49 | -1.362 | 2.84 |
| Positive Deviation Count (Basic Model) | APOE: e4 homozygotes | -1.261 | 1.703 | -0.741 | 0.459 | -4.601 | 2.079 |
| Positive Deviation Count (Basic Model) | Age | 0.087 | 0.055 | 1.598 | 0.11 | -0.02 | 0.194 |
| Positive Deviation Count (Basic Model) | Sex | 2.517 | 0.975 | 2.582 | 0.01 | 0.604 | 4.43 |
| Negative Deviation Count (Sex Interaction Model) | Intercept | 16.457 | 3.159 | 5.209 | <0.01 | 10.259 | 22.654 |
| Negative Deviation Count (Sex Interaction Model) | APOE: e2 carriers | 1.563 | 2.261 | 0.691 | 0.49 | -2.872 | 5.998 |
| Negative Deviation Count (Sex Interaction Model) | APOE: e4 heterozygotes | 0.236 | 1.104 | 0.214 | 0.831 | -1.931 | 2.403 |
| Negative Deviation Count (Sex Interaction Model) | APOE: e4 homozygotes | 7.485 | 1.86 | 4.024 | <0.01 | 3.836 | 11.133 |
| Negative Deviation Count (Sex Interaction Model) | Sex | -1.843 | 1.12 | -1.645 | 0.1 | -4.041 | 0.355 |
| Negative Deviation Count (Sex Interaction Model) | APOE x Sex: e2 carriers | -2.159 | 2.994 | -0.721 | 0.471 | -8.033 | 3.715 |
| Negative Deviation Count (Sex Interaction Model) | APOE x Sex: e4 heterozygotes | -0.282 | 1.617 | -0.175 | 0.861 | -3.454 | 2.89 |
| Negative Deviation Count (Sex Interaction Model) | APOE x Sex: e4 homozygotes | -7.042 | 2.556 | -2.755 | 0.006 | -12.056 | -2.027 |
| Negative Deviation Count (Sex Interaction Model) | Age | -0.121 | 0.041 | -2.92 | 0.004 | -0.201 | -0.04 |

Supplementary Table 6 reports post-hoc pairwise comparisons using Tukey's HSD test. For positive deviations, only the comparison between ε2 carriers and ε4 homozygotes was significant (p = 0.046). For negative deviations, ε4 homozygotes differed significantly from both ε3 homozygotes (p = 0.009) and ε4 heterozygotes (p = 0.018).

**Supplementary Table 6** - Post-hoc Tukey HSD tests comparing APOE genotype groups on deviation counts.

| **Outcome** | **Group 1** | **Group 2** | **Mean Difference** | **p-value (adj)** | **CI Lower** | **CI Upper** |
| --- | --- | --- | --- | --- | --- | --- |
| Positive Deviation Count | e2 carriers | e3 homozygotes | -4.748 | 0.075 | -9.809 | 0.313 |
| Positive Deviation Count | e2 carriers | e4 heterozygotes | -4.147 | 0.155 | -9.235 | 0.942 |
| Positive Deviation Count | e2 carriers | e4 homozygotes | -6.188 | 0.046 | -12.295 | -0.082 |
| Positive Deviation Count | e3 homozygotes | e4 heterozygotes | 0.601 | 0.944 | -2.159 | 3.361 |
| Positive Deviation Count | e3 homozygotes | e4 homozygotes | -1.44 | 0.831 | -5.801 | 2.921 |
| Positive Deviation Count | e4 heterozygotes | e4 homozygotes | -2.041 | 0.63 | -6.434 | 2.351 |
| Negative Deviation Count | e2 carriers | e3 homozygotes | -0.492 | 0.988 | -4.336 | 3.351 |
| Negative Deviation Count | e2 carriers | e4 heterozygotes | -0.239 | 0.999 | -4.103 | 3.625 |
| Negative Deviation Count | e2 carriers | e4 homozygotes | 3.568 | 0.196 | -1.069 | 8.205 |
| Negative Deviation Count | e3 homozygotes | e4 heterozygotes | 0.254 | 0.99 | -1.842 | 2.35 |
| Negative Deviation Count | e3 homozygotes | e4 homozygotes | 4.06 | 0.009 | 0.749 | 7.372 |
| Negative Deviation Count | e4 heterozygotes | e4 homozygotes | 3.807 | 0.018 | 0.471 | 7.142 |

## **Neurobiology Maps**

We report additional analyses with cytoarchitectonic, microstructural, and transcriptional maps. While not the primary focus of our hypotheses, these complementary measures extend the scope of our findings and may inspire future directions for translating imaging markers of AD into biological mechanisms (**Supplementary Figure 10**). Full results of the spin-tests for these analyses are in **Supplementary Table 7** below and detailed descriptions of the maps in **subsection 6.1**.


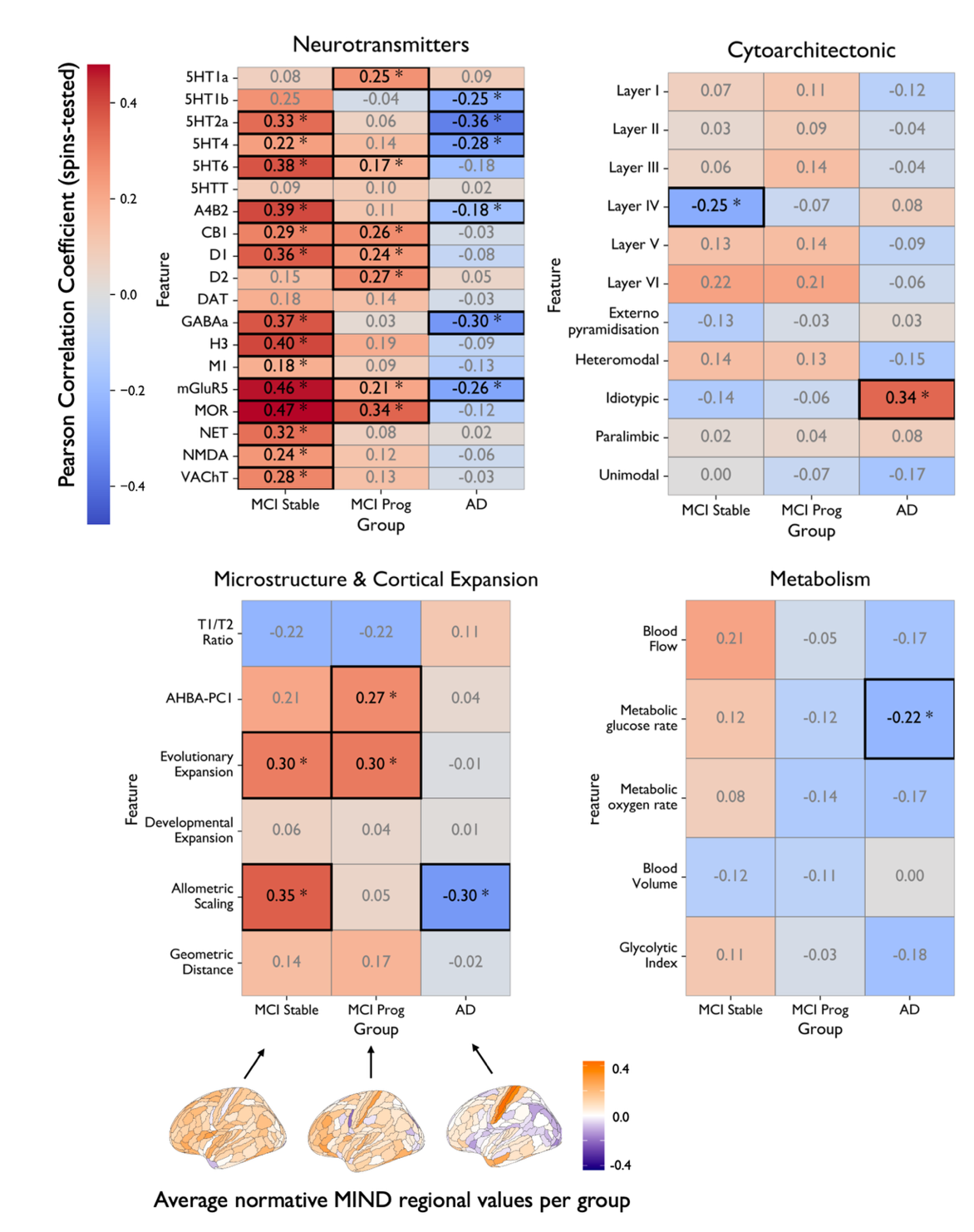


**Supplementary Figure 10 - Biological decoding of group-average deviation maps.** Heatmaps show Pearson correlation coefficients (r) between average regional MIND deviation maps (Z-scores) for each diagnostic group (columns: MCI Stable, MCI Progressive, AD) and a range of biological reference maps (rows), spanning four domains: neurotransmitter receptor density, cytoarchitecture, metabolism, and microstructure/ cortical expansion. All MIND values are averaged across both hemispheres (left and right). Correlations were tested for statistical significance using spin permutation testing (N = 5000) to control for spatial autocorrelation; significant values (p < 0.05, spin-corrected) are marked with asterisks and outlined in black. Colour indicates the direction and magnitude of the correlation (red = positive, blue = negative). Group-average deviation maps are visualized below for reference with their corresponding legend. AHBA = Allen Human Brain Atlas. * indicates statistically significant differences (p < 0.05).

**Supplementary Table 7** - Full details of the spin-test correlation analyses (N=5000 spins, Hungarian Method)

| **Feature** | **MCI Stable pval (spin)** | **MCI Progressive pval (spin)** | **AD pval (spin)** |
| --- | --- | --- | --- |
| **Metabolic oxygen rate** | 0.572 | 0.233 | 0.136 |
| **Metabolic glucose rate** | 0.374 | 0.327 | 0.029 |
| **Blood Volume** | 0.189 | 0.347 | 0.985 |
| **Blood Flow** | 0.082 | 0.709 | 0.106 |
| **Evolutionary Expansion** | 0.007 | 0.007 | 0.953 |
| **Developmental Expansion** | 0.538 | 0.673 | 0.944 |
| **Glycolytic Index** | 0.361 | 0.802 | 0.058 |
| **Cortical Thickness** | 0.109 | 0.076 | 0.322 |
| **Cortical Curvature** | 0.208 | 0.757 | 0.763 |
| **AHBA-PC1** | 0.17 | 0.04 | 0.724 |
| **Neurosynth-PC1** | 0.472 | 0.258 | 0.908 |
| **T1/T2 Ratio** | 0.07 | 0.075 | 0.32 |
| **Allometric Scaling** | <0.001 | 0.534 | <0.001 |
| **Geometric Distance** | 0.403 | 0.246 | 0.852 |
| **5HT1a** | 0.664 | 0.035 | 0.389 |
| **5HT1b** | 0.052 | 0.766 | 0.018 |
| **5HT2a** | 0.001 | 0.516 | 0.001 |
| **5HT4** | 0.021 | 0.261 | 0.017 |
| **5HT6** | <0.001 | 0.043 | 0.066 |
| **5HTT** | 0.499 | 0.316 | 0.817 |
| **A4B2** | <0.001 | 0.375 | 0.039 |
| **CB1** | 0.01 | 0.011 | 0.716 |
| **D1** | <0.001 | 0.017 | 0.358 |
| **D2** | 0.302 | 0.022 | 0.638 |
| **DAT** | 0.253 | 0.189 | 0.672 |
| **GABAa** | 0.003 | 0.745 | 0.006 |
| **H3** | <0.001 | 0.083 | 0.324 |
| **M1** | 0.043 | 0.381 | 0.289 |
| **mGluR5** | <0.001 | 0.038 | 0.011 |
| **MOR** | <0.001 | 0.001 | 0.282 |
| **NET** | 0.013 | 0.491 | 0.887 |
| **NMDA** | 0.026 | 0.166 | 0.456 |
| **VAChT** | 0.016 | 0.13 | 0.779 |
| **Layer I** | 0.576 | 0.30 | 0.248 |
| **Layer II** | 0.794 | 0.438 | 0.66 |
| **Layer III** | 0.622 | 0.177 | 0.662 |
| **Layer IV** | 0.042 | 0.69 | 0.361 |
| **Layer V** | 0.261 | 0.217 | 0.367 |
| **Layer VI** | 0.057 | 0.065 | 0.559 |
| **Externo-pyramidisation** | 0.294 | 0.868 | 0.783 |
| **Heteromodal** | 0.139 | 0.287 | 0.169 |
| **Idiotypic** | 0.144 | 0.535 | 0.002 |
| **Paralimbic** | 0.85 | 0.667 | 0.411 |
| **Unimodal** | 0.993 | 0.518 | 0.093 |

## **Detailed Descriptions of the Maps Used**

Biological reference maps were drawn from established open-access repositories and covered a wide range of cortical properties. These included neurotransmitter receptor density distributions derived from PET meta-analyses (e.g., for serotonin 5-HT2a, dopamine D1, GABAa, and nicotinic α4β2 receptors —originally compiled by Hansen et al. (2022)). For cytoarchitectonic features, we used information available from the BigBrain Atlas (Wagstyl et al., 2020) alongside available maps used in previous work (Mesulam, 1998; Paquola et al., 2019; Vogel et al., 2024) that provided information about cortical metabolism (cerebral blood flow, glucose metabolism), microstructural proxies such as myelin (T1w/T2w), cortical hierarchy (Mesulam), developmental/evolutionary expansion maps (Sydnor et al., 2021), and for transcriptomic the first principal component from the Allen Human Brain Atlas (Hawrylycz et al., 2012). All these maps were retrieved from prior original work (Hansen et al., 2022; Markello et al., 2022a; Mesulam, 1998; Paquola et al., 2019; Sydnor et al., 2021; Vogel et al., 2024; Wagstyl et al., 2020) or from the *neuromaps* toolbox (Markello et al., 2022b).

Detailed descriptions for each are provided below:

**Neurotransmitters**To create a detailed cortical atlas of receptor distributions, researchers from the MNI (Hansen, Shafiei, Markello, et al., 2022) combined PET data for 19 receptors, transporters, and binding sites across nine neurotransmitter systems: dopamine, norepinephrine, serotonin, acetylcholine, glutamate, GABA, histamine, cannabinoids, and opioids. The paper provides a comprehensive overview of the datasets, including tracer information, receptor/transporter types, participant numbers, demographics, scanner specifications, modelling details, reference regions, scan durations, and source references. All data were obtained from healthy participants (n = 1,238; 718 males and 520 females), with group-average maps generated at the study level. Each PET image was normalised to the MNI-ICBM 152 nonlinear 2009c asymmetric template. When multiple tracer averages were available for the same receptor or transporter (e.g., 5-HT1B, D2, mGluR5, VAChT), weighted averages were calculated after confirming high similarity between maps. After parcellation, all receptor/transporter maps were combined into a receptor-by-region matrix that represented standardised density estimates.

**Myelination**
To estimate intracortical myelin, we utilised processed T1w/T2w ratio maps from the Human Connectome Project (<https://www.humanconnectome.org/>) (HCP, S1200 release), which were provided in surface-based CIFTI format for 417 unrelated individuals (193 males; ages 22–37 years). Images were acquired on a Siemens Skyra 3T scanner using both a T1-weighted MPRAGE and a T2-weighted SPACE sequence, each with an isotropic resolution of 0.7 mm. The complete acquisition protocols and preprocessing steps are publicly accessible (Van Essen et al., 2013).

**Gene expression PC1**
Gene expression data were obtained from the Allen Human Brain Atlas (Hawrylycz et al., 2012) and processed using abagen (Markello et al., 2021). From the resulting region-by-gene matrix, 11,560 genes with differential stability above 0.1 were retained. The first principal component of gene expression, termed the “gene gradient”, was then extracted from the left hemisphere and subsequently mirrored onto the right hemisphere. This gradient has been linked to patterns of cell type distribution and cell-specific gene expression, indicating that it reflects the cellular architecture of the brain (Hansen, Shafiei, Vogel, et al., 2022).

**Developmental expansion**To understand developmental differences in cortical structure, we utilised previously generated maps that compared MRI data from infants and adults. Participant characteristics, as well as MRI acquisition and image-processing procedures, have been described in detail elsewhere (Hill et al., 2010). The study included 12 healthy, term-born infants (mean gestational age, 39 weeks; six males and six females) from uncomplicated pregnancies, as well as 12 neurologically normal, right-handed young adults (aged 18–24 years; six males and six females). Cortical surface reconstructions were generated from these subjects. Reconstructions from the infant group were compared with those from the adult group. A map of cortical surface expansion was created by evaluating the average proportion of total surface area assigned to each cortical tile in adults relative to its corresponding proportion in infants.

**Evolutionary expansion**Prior work ran a series of analyses to facilitate cross-species comparisons of cortical organisation (Hill et al., 2010). A surface atlas from a single macaque monkey (F99) was aligned with the adult PALS-B12 atlas using a combination of structural and functional homologies. Subsequently, a previous map demonstrating evolutionary cortical enlargement between the macaque and the human adult was aligned to the PALS-TA24 atlas. Finally, maps illustrating postnatal surface area growth and evolutionary expansion were smoothed over 10 iterations with a 5-mm kernel using an average-neighbour method.

**Allometric scaling**Across primate evolution and human development, changes in brain size are linked to alterations in the relative proportions of different brain regions. To explore this in humans, 2,904 structural MRI scans were previously analysed from two groups: 1,373 cross-sectional scans from the Philadelphia Neurodevelopmental Cohort (ages 8–23 years, 3 Tesla) and 1,531 longitudinal scans from 792 participants in the NIH cohort (ages 5–25 years, 1.5 Tesla) (Reardon et al., 2018). Local surface area at approximately 80,000 cortical points per scan (“vertex area”) was measured using an automated image-processing pipeline, and vertex-specific scaling was estimated with semiparametric generalised additive models. Within this framework, coefficients of 1 indicated linear scaling, meaning the region grows proportionally with overall brain size; values greater than 1 indicated positive allometric scaling, where a region expands disproportionately and becomes relatively larger in bigger brains; and values less than 1 indicated negative allometric scaling, where a region grows more slowly and occupies a proportionally smaller share as brain size increases.

**Geodesic distance**To assess the spatial centrality or isolation of cortical regions, data from the Human Connectome Project (HCP) was used to calculate the average surface-traversing distance from each vertex to all others (Margulies et al., 2016). Geodesic distances were determined using an algorithm that estimates the shortest path between two nodes on a triangular surface mesh. Unlike the more commonly used Dijkstra’s algorithm, this exact geodesic approach produces distance values that are independent of mesh density.

**Cytoarchitecture**To examine the histological features of the human cerebral cortex, we used **BigBrain**, a three-dimensional atlas of a post-mortem male brain (aged 65 years). BigBrain was generated from the digital reconstruction of ultrahigh-resolution (20 μm) cell body–stained sections and is publicly accessible at <https://ftp.bigbrainproject.org/>. The cerebral cortex was segmented into six layers using a convolutional neural network trained on manually segmented samples from expert anatomists. Laminar thickness data are available through the BigBrainWarp toolbox (<https://bigbrainwarp.readthedocs.io>). In addition, **externopyramidisation**—defined as the ratio of supragranular to infragranular pyramidal neuron soma size—was used as a measure of laminar specialisation. Detailed analytical procedures have been described elsewhere (Wagstyl et al., 2020).

**Cortical hierarchy**

The Mesulam classes (Mesulam, 1998) represent both the anatomical laminar structure and characteristic patterns of cortical connectivity, offering a framework to link structural organisation with functional network architecture. The Mesulam classification distinguishes four levels of laminar differentiation—**idiotypic, unimodal, heteromodal, and paralimbic**—based on a combination of neuroanatomical, electrophysiological, and behavioural studies in humans and non-human primates. The assignments of laminar differentiation to the cortical surface were carried out manually (Paquola et al., 2019).

**Brain metabolism**Positron emission tomography (PET) has previously been used (Vaishnavi et al., 2010) to quantify cerebral blood flow (CBF), cerebral blood volume (CBV), cerebral metabolic rate for oxygen (CMRO₂), and cerebral metabolic rate for glucose (CMRGlu) in 33 healthy, right-handed adults during a resting, awake state with eyes closed. Regional glucose metabolism was assessed with [¹⁸F]-labelled fluorodeoxyglucose (FDG), while oxygen metabolism required three separate scans using [¹⁵O]-labelled water, carbon monoxide, and oxygen. For each subject, regional CMRO₂ and CMRGlu values were normalised to a whole-brain mean of 1 (local-to-global ratio), and the group results were subsequently averaged in a standard atlas space. **To quantify aerobic glycolysis, the conventional approach of calculating the oxygen–glucose index (OGI), which is defined as the molar ratio of oxygen consumption to glucose utilisation, was employed.**

**References for Supplementary Neurobiology Maps**

Hansen, J. Y., Shafiei, G., Markello, R. D., Smart, K., Cox, S. M. L., Nørgaard, M., Beliveau, V., Wu, Y., Gallezot, J.-D., Aumont, É., Servaes, S., Scala, S. G., DuBois, J. M., Wainstein, G., Bezgin, G., Funck, T., Schmitz, T. W., Spreng, R. N., Galovic, M., . . . Misic, B. (2022). Mapping neurotransmitter systems to the structural and functional organization of the human neocortex. *Nature Neuroscience*, *25*(11), 1569-1581. <https://doi.org/10.1038/s41593-022-01186-3>

Hawrylycz, M. J., Lein, E. S., Guillozet-Bongaarts, A. L., Shen, E. H., Ng, L., Miller, J. A., van de Lagemaat, L. N., Smith, K. A., Ebbert, A., Riley, Z. L., Abajian, C., Beckmann, C. F., Bernard, A., Bertagnolli, D., Boe, A. F., Cartagena, P. M., Chakravarty, M. M., Chapin, M., Chong, J., . . . Jones, A. R. (2012). An anatomically comprehensive atlas of the adult human brain transcriptome. *Nature*, *489*(7416), 391-399. <https://doi.org/10.1038/nature11405>

Hill, J., Inder, T., Neil, J., Dierker, D., Harwell, J., & Van Essen, D. (2010). Similar patterns of cortical expansion during human development and evolution. *Proceedings of the National Academy of Sciences of the United States of America*, *107*(29), 13135-13140. <https://doi.org/10.1073/pnas.1001229107>

Margulies, D. S., Ghosh, S. S., Goulas, A., Falkiewicz, M., Huntenburg, J. M., Langs, G., Bezgin, G., Eickhoff, S. B., Castellanos, F. X., Petrides, M., Jefferies, E., & Smallwood, J. (2016). Situating the default-mode network along a principal gradient of macroscale cortical organization. *Proceedings of the National Academy of Sciences of the United States of America*, *113*(44), 12574-12579. <https://doi.org/10.1073/pnas.1608282113>

Markello, R. D., Arnatkeviciute, A., Poline, J. B., Fulcher, B. D., Fornito, A., & Misic, B. (2021). Standardizing workflows in imaging transcriptomics with the abagen toolbox. *Elife*, *10*. <https://doi.org/10.7554/eLife.72129>

Markello RD, Hansen JY, Liu ZQ, et al. neuromaps: structural and functional interpretation of brain maps. *Nat Methods*. 2022;19(11):1472-1479. doi:10.1038/s41592-022-01625-w

Mesulam, M. M. (1998). From sensation to cognition. *Brain*, *121 ( Pt 6)*, 1013-1052. <https://doi.org/10.1093/brain/121.6.1013>

Paquola, C., Bethlehem, R. A., Seidlitz, J., Wagstyl, K., Romero-Garcia, R., Whitaker, K. J., Vos de Wael, R., Williams, G. B., Vértes, P. E., Margulies, D. S., Bernhardt, B., & Bullmore, E. T. (2019). Shifts in myeloarchitecture characterise adolescent development of cortical gradients. *Elife*, *8*. <https://doi.org/10.7554/eLife.50482>

Reardon, P. K., Seidlitz, J., Vandekar, S., Liu, S., Patel, R., Park, M. T. M., Alexander-Bloch, A., Clasen, L. S., Blumenthal, J. D., Lalonde, F. M., Giedd, J. N., Gur, R. C., Gur, R. E., Lerch, J. P., Chakravarty, M. M., Satterthwaite, T. D., Shinohara, R. T., & Raznahan, A. (2018). Normative brain size variation and brain shape diversity in humans. *Science*, *360*(6394), 1222-1227. <https://doi.org/10.1126/science.aar2578>

Vaishnavi, S. N., Vlassenko, A. G., Rundle, M. M., Snyder, A. Z., Mintun, M. A., & Raichle, M. E. (2010). Regional aerobic glycolysis in the human brain. *Proceedings of the National Academy of Sciences of the United States of America*, *107*(41), 17757-17762. <https://doi.org/10.1073/pnas.1010459107>

Van Essen, D. C., Smith, S. M., Barch, D. M., Behrens, T. E., Yacoub, E., & Ugurbil, K. (2013). The WU-Minn Human Connectome Project: an overview. *Neuroimage*, *80*, 62-79. <https://doi.org/10.1016/j.neuroimage.2013.05.041>

Wagstyl, K., Larocque, S., Cucurull, G., Lepage, C., Cohen, J. P., Bludau, S., Palomero-Gallagher, N., Lewis, L. B., Funck, T., Spitzer, H., Dickscheid, T., Fletcher, P. C., Romero, A., Zilles, K., Amunts, K., Bengio, Y., & Evans, A. C. (2020). BigBrain 3D atlas of cortical layers: Cortical and laminar thickness gradients diverge in sensory and motor cortices. *PLoS Biol*, *18*(4), e3000678. <https://doi.org/10.1371/journal.pbio.3000678>

## **Postmortem Neuropathology Analyses**

From the full NACC dataset, we identified *N*=558 participants with both MRI data and completed postmortem information. After excluding participants with invalid pathology scores (missing or not possible to score: 8 = Not assessed, - 9 = Missing/unknown, -4 = Not available) and restricting to those with complete data for all covariates, our final analytic sample included *N=*240 participants. The demographic and clinical characteristics of this subset are summarized in Supplementary Table 8 below, with the distribution of ABC scores across diagnoses in Supplementary Figure 11.

**Supplementary Table 8**- Demographic and Clinical Characteristics of the Autopsy Sample (N=240)

| **Characteristic** | **Overall (N=240)** | **ABC 0 (N=20)** | **ABC 1 (N=13)** | **ABC 2 (N=40)** | **ABC 3 (N=167)** |
| --- | --- | --- | --- | --- | --- |
| **Diagnosis, n (%)** |  |  |  |  |  |
| AD | 144 (60.0%) | 5 (25.0%) | 6 (46.2%) | 14 (35.0%) | 119 (71.3%) |
| MCI Progressive | 69 (28.8%) | 6 (30.0%) | 4 (30.8%) | 17 (42.5%) | 42 (25.1%) |
| MCI Stable | 27 (11.2%) | 9 (45.0%) | 3 (23.1%) | 9 (22.5%) | 6 (3.6%) |
| **Age at MRI, mean (SD)** | 76.3 (10.2) | 72.1 (10.5) | 75.8 (9.7) | 75.4 (10.3) | 77.2 (10.0) |
| **Gender, n (%)** |  |  |  |  |  |
| Female | 142 (59.2%) | 12 (60.0%) | 7 (53.8%) | 24 (60.0%) | 99 (59.3%) |
| Male | 98 (40.8%) | 8 (40.0%) | 6 (46.2%) | 16 (40.0%) | 68 (40.7%) |
| **Time to death (years), mean (SD)** | 3.2 (2.5) | 3.5 (2.7) | 3.3 (2.4) | 3.4 (2.6) | 3.1 (2.4) |
| **Negative deviations, mean (SD)** | 9.5 (18.6) | 7.2 (5.4) | 5.3 (4.6) | 6.9 (9.2) | 10.9 (21.5) |
| **Positive deviations, mean (SD)** | 10.2 (12.3) | 10.5 (11.7) | 9.8 (14.0) | 10.3 (11.5) | 10.1 (12.5) |


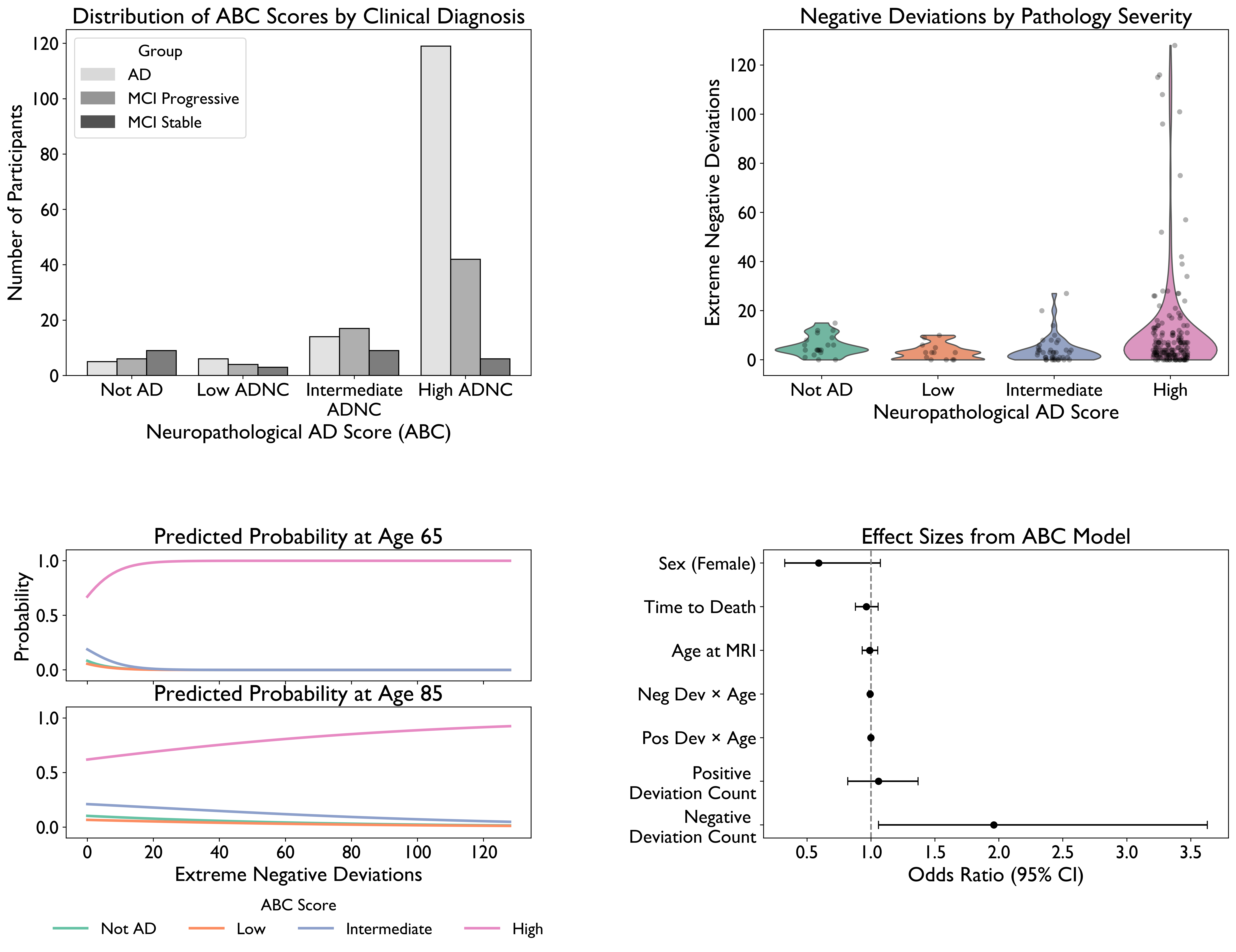


**Supplementary Figure 11**- Histogram showing the distribution of ABC scores (0-3) across diagnostic groups (AD, MCI Progressive, and MCI Stable). Note the higher prevalence of elevated ABC scores (score 3) in the AD group compared to MCI groups.

Neuropathological analyses were performed according to the National Institute on Aging–Alzheimer's Association (NIA-AA) guidelines for the assessment of Alzheimer's disease neuropathologic change (ADNC). All cases underwent standardized autopsy procedures at NACC-affiliated centres. The **ABC** scoring system integrates three key pathological processes:

- **A score (Amyloid-β deposition)**: Based on Thal phase, ranging from 0 (no amyloid) to 5 (widespread distribution)
- **B score (Braak neurofibrillary tangle stage)**: Based on tau pathology distribution, ranging from 0 (none) to 6 (extensive neocortical)
- **C score (CERAD neuritic plaque score)**: Measures neuritic plaque density, ranging from 0 (none) to 3 (frequent)

These individual scores were used to derive the composite ABC score (NPADNC in the NACC dataset), a summary measure of AD pathological change ranging from 0 (not AD) to 3 (high-level AD neuropathologic change).

### **Statistical Analysis Approach**

We analysed the relationship between structural brain deviations and neuropathological measures using ordinal logistic regression models implemented in the statsmodels Python package (version 0.14.0). To account for the ordinal nature of the ABC score, we used the following model specifications:

#### Basic Model

$$logit(P(ABC\_score \leq k)) = \alphaₖ - (\beta₁ \times neg\_dev\_count + \beta₂ \times pos\_dev\_count + \beta₃ \times age\_at\_MRI + \beta₄ \times sex + \beta₅ \times time\_to\_death)$$

#### Age Interaction Model

$$logit(P(ABC\_score \leq k)) = \alphaₖ - (\beta₁ \times neg\_dev\_count + \beta₂ \times pos\_dev\_count + \beta₃ \times age\_at\_MRI + \beta₄ \times sex + \beta₅ \times time\_to\_death + \beta₆ \times neg\_dev\_count \times age\_at\_MRI + \beta₇ \times pos\_dev\_count \times age\_at\_MRI)$$

#### Time-to-Death Interaction Model

$$logit(P(ABC\_score \leq k)) = \alphaₖ - (\beta₁ \times neg\_dev\_count + \beta₂ \times pos\_dev\_count + \beta₃ \times age\_at\_MRI + \beta₄ \times sex + \beta₅ \times time\_to\_death + \beta₆ \times neg\_dev\_count \times time\_to\_death + \beta₇ \times pos\_dev\_count \times time\_to\_death)$$

Where:

- neg_dev_count and pos_dev_count are the total count of extreme negative and positive deviations (below -1.96 and above 1.96)
- age_at_MRI is the participant's age at MRI acquisition
- sex is coded as 0 (female) or 1 (male)
- time_to_death is the time interval between MRI acquisition and death
- αₖ are the threshold parameters for each level k of the ABC score

Model comparison was performed using the Akaike Information Criterion (AIC), with lower values indicating better relative model fit. The same modelling approach was applied separately to the individual A, B, and C component scores.

### **Full Model Results for ABC Score Prediction**

**Supplementary Table 9** – Full model results (best model) for the ABC Score Prediction

| 1. **Basic Model (AIC = 428.7)** | | | | | |
| --- | --- | --- | --- | --- | --- |
| **Parameter** | **Coefficient** | **Std Error** | **z-value** | **p-value** | **OR [95% CI]** |
| num_extreme_negative_deviations | 0.0536 | 0.026 | 2.056 | 0.040 | 1.06 [1.00-1.11] |
| num_extreme_positive_deviations | 0.0199 | 0.013 | 1.570 | 0.116 | 1.02 [0.99-1.05] |
| age_at_MRI | -0.0539 | 0.019 | -2.841 | 0.004 | 0.95 [0.91-0.98] |
| sex | -0.5456 | 0.302 | -1.806 | 0.071 | 0.58 [0.32-1.05] |
| time_to_death | -0.0534 | 0.046 | -1.160 | 0.246 | 0.95 [0.87-1.04] |
| 0.0/1.0 | -6.7700 | 1.642 | -4.122 | <0.001 | - |
| 1.0/2.0 | -0.5532 | 0.272 | -2.031 | 0.042 | - |
| 2.0/3.0 | 0.0883 | 0.149 | 0.594 | 0.552 | - |

| 1. **Age Interaction Model (AIC = 427.8)** | | | | | |
| --- | --- | --- | --- | --- | --- |
| **Parameter** | **Coefficient** | **Std Error** | **z-value** | **p-value** | **OR [95% CI]** |
| num_extreme_negative_deviations | 0.6732 | 0.314 | 2.142 | 0.032 | 1.96 [1.06-3.63] |
| num_extreme_positive_deviations | 0.0566 | 0.131 | 0.432 | 0.666 | 1.06 [0.82-1.37] |
| age_at_MRI | -0.0096 | 0.031 | -0.309 | 0.758 | 0.99 [0.93-1.05] |
| sex | -0.5247 | 0.304 | -1.726 | 0.084 | 0.59 [0.33-1.07] |
| time_to_death | -0.0375 | 0.047 | -0.798 | 0.425 | 0.96 [0.88-1.06] |
| negdev_x_age | -0.0077 | 0.004 | -2.040 | 0.041 | 0.99 [0.98-1.00] |
| posdev_x_age | -0.0005 | 0.002 | -0.271 | 0.786 | 1.00 [1.00-1.00] |
| 0.0/1.0 | -3.1284 | 2.561 | -1.221 | 0.222 | - |
| 1.0/2.0 | -0.5542 | 0.272 | -2.035 | 0.042 | - |
| 2.0/3.0 | 0.0952 | 0.148 | 0.643 | 0.520 | - |

| 1. **Time-to-Death Interaction Model (AIC = 429.9)** | | | | | |
| --- | --- | --- | --- | --- | --- |
| **Parameter** | **Coefficient** | **Std Error** | **z-value** | **p-value** | **OR [95% CI]** |
| num_extreme_negative_deviations | 0.1325 | 0.069 | 1.930 | 0.054 | 1.14 [1.00-1.31] |
| num_extreme_positive_deviations | 0.0625 | 0.037 | 1.709 | 0.087 | 1.06 [0.99-1.14] |
| age_at_MRI | -0.0530 | 0.019 | -2.746 | 0.006 | 0.95 [0.91-0.99] |
| sex | -0.5503 | 0.302 | -1.822 | 0.068 | 0.58 [0.32-1.04] |
| time_to_death | 0.0348 | 0.069 | 0.506 | 0.613 | 1.04 [0.90-1.18] |
| negdev_x_ttd | -0.0103 | 0.007 | -1.385 | 0.166 | 0.99 [0.98-1.00] |
| posdev_x_ttd | -0.0053 | 0.004 | -1.295 | 0.195 | 0.99 [0.99-1.00] |
| 0.0/1.0 | -6.0308 | 1.711 | -3.526 | <0.001 | - |
| 1.0/2.0 | -0.5562 | 0.273 | -2.041 | 0.041 | - |
| 2.0/3.0 | 0.0966 | 0.148 | 0.651 | 0.515 | - |

To examine whether the relationship between structural deviation patterns and pathology burden differed by clinical diagnosis, we also stratified our analyses by diagnostic group (AD, MCI Progressive, MCI Stable). The results (Supplementary Table 10) revealed consistent patterns across diagnostic groups, with negative deviations showing positive associations with ABC score in both AD and MCI Progressive groups, although with varying effect sizes. In the AD group, there was a stronger effect of negative deviations (OR = 3.48, 95% CI: 0.75-16.01) and a more pronounced age interaction, suggesting that younger AD patients with high deviation counts may have particularly severe pathology. The MCI Stable group showed an opposite pattern (OR = 0.28), although with wide confidence intervals reflecting the small sample size (N=27).

**Supplementary Table 10** - ABC Models Stratified by Diagnostic Group

#### **A) AD Group (N=144, Model: Age Interaction)**

| Parameter | Coefficient | Std Error | z-value | p-value | OR [95% CI] |
| --- | --- | --- | --- | --- | --- |
| num_extreme_negative_deviations | 1.2478 | 0.780 | 1.599 | 0.110 | 3.48 [0.75-16.01] |
| num_extreme_positive_deviations | 0.0562 | 0.326 | 0.172 | 0.863 | 1.06 [0.56-2.01] |
| age_at_MRI | -0.0565 | 0.062 | -0.916 | 0.360 | 0.95 [0.84-1.07] |
| sex[male] | -0.7897 | 0.572 | -1.381 | 0.167 | 0.45 [0.15-1.39] |
| time_to_death | -0.0340 | 0.088 | -0.389 | 0.697 | 0.97 [0.81-1.15] |
| negdev_x_age | -0.0145 | 0.009 | -1.572 | 0.116 | 0.99 [0.97-1.00] |
| posdev_x_age | -0.0002 | 0.004 | -0.049 | 0.961 | 1.00 [0.99-1.01] |

#### **B) MCI Progressive Group (N=69, Model: Age Interaction)**

| Parameter | Coefficient | Std Error | z-value | p-value | OR [95% CI] |
| --- | --- | --- | --- | --- | --- |
| num_extreme_negative_deviations | 0.5179 | 0.500 | 1.036 | 0.300 | 1.68 [0.63-4.47] |
| num_extreme_positive_deviations | 0.1462 | 0.287 | 0.509 | 0.611 | 1.16 [0.66-2.03] |
| age_at_MRI | 0.0346 | 0.066 | 0.528 | 0.598 | 1.04 [0.91-1.18] |
| sex[male] | -0.2695 | 0.506 | -0.533 | 0.594 | 0.76 [0.28-2.06] |
| time_to_death | 0.1095 | 0.094 | 1.168 | 0.243 | 1.12 [0.93-1.34] |
| negdev_x_age | -0.0062 | 0.006 | -0.988 | 0.323 | 0.99 [0.98-1.01] |
| posdev_x_age | -0.0022 | 0.004 | -0.562 | 0.574 | 1.00 [0.99-1.01] |

#### **C) MCI Stable Group (N=27, Model: Age Interaction)**

| **Parameter** | **Coefficient** | **Std Error** | **z-value** | **p-value** | **OR [95% CI]** |
| --- | --- | --- | --- | --- | --- |
| num_extreme_negative_deviations | -1.2809 | 1.464 | -0.875 | 0.382 | 0.28 [0.02-4.93] |
| num_extreme_positive_deviations | 0.7670 | 0.967 | 0.793 | 0.428 | 2.15 [0.32-14.33] |
| age_at_MRI | 0.0311 | 0.114 | 0.272 | 0.786 | 1.03 [0.82-1.29] |
| sex[male] | -0.8382 | 0.931 | -0.901 | 0.368 | 0.43 [0.07-2.68] |
| time_to_death | -0.1304 | 0.112 | -1.163 | 0.245 | 0.88 [0.70-1.09] |
| negdev_x_age | 0.0137 | 0.017 | 0.812 | 0.417 | 1.01 [0.98-1.05] |
| posdev_x_age | -0.0104 | 0.012 | -0.849 | 0.396 | 0.99 [0.97-1.01] |

### **Results from Models of Individual A, B, C Components**

To investigate which aspects of AD pathology were most strongly associated with structural deviations, we examined models for each component of the ABC score (Supplementary Table 11). The B score (Braak stage) showed the strongest association with negative deviations, particularly when accounting for age interactions. This suggests that the tau-related aspects of AD pathology may be more closely linked to structural network disruptions than amyloid pathology. The C score (neuritic plaque density) showed minimal association with deviation counts, suggesting that structural deviation patterns may not be strongly related to this aspect of AD pathology.

**Supplementary Table 11 –** Results from models of individual A, B and C Components

#### **A) A Score Model (AIC = 593.6)**

| **Parameter** | **Coefficient** | **Std Error** | **z-value** | **p-value** | **OR [95% CI]** |
| --- | --- | --- | --- | --- | --- |
| num_extreme_negative_deviations | 0.0111 | 0.009 | 1.272 | 0.204 | 1.01 [0.99-1.03] |
| num_extreme_positive_deviations | 0.0031 | 0.007 | 0.422 | 0.673 | 1.00 [0.99-1.02] |
| age_at_MRI | -0.0442 | 0.016 | -2.805 | 0.005 | 0.96 [0.93-0.99] |
| sex[male] | -0.6911 | 0.268 | -2.580 | 0.010 | 0.50 [0.30-0.85] |
| time_to_death | -0.0544 | 0.041 | -1.317 | 0.188 | 0.95 [0.87-1.03] |

#### **B) B Score Model with Age Interaction (AIC = 651.8)**

| **Parameter** | **Coefficient** | **Std Error** | **z-value** | **p-value** | **OR [95% CI]** |
| --- | --- | --- | --- | --- | --- |
| num_extreme_negative_deviations | 0.2534 | 0.196 | 1.294 | 0.196 | 1.29 [0.88-1.89] |
| num_extreme_positive_deviations | -0.1087 | 0.077 | -1.408 | 0.159 | 0.90 [0.77-1.04] |
| age_at_MRI | -0.0539 | 0.026 | -2.110 | 0.035 | 0.95 [0.90-1.00] |
| sex[male] | -0.4626 | 0.268 | -1.728 | 0.084 | 0.63 [0.37-1.06] |
| time_to_death | 0.0207 | 0.042 | 0.494 | 0.621 | 1.02 [0.94-1.11] |
| negdev_x_age | -0.0027 | 0.002 | -1.100 | 0.271 | 1.00 [0.99-1.00] |
| posdev_x_age | 0.0016 | 0.001 | 1.495 | 0.135 | 1.00 [1.00-1.00] |

#### **C) C Score Model (AIC = 489.3)**

| **Parameter** | **Coefficient** | **Std Error** | **z-value** | **p-value** | **OR [95% CI]** |
| --- | --- | --- | --- | --- | --- |
| num_extreme_negative_deviations | -0.0024 | 0.007 | -0.362 | 0.718 | 1.00 [0.99-1.01] |
| num_extreme_positive_deviations | -0.0005 | 0.008 | -0.066 | 0.947 | 1.00 [0.98-1.02] |
| age_at_MRI | -0.0593 | 0.017 | -3.437 | 0.001 | 0.94 [0.91-0.97] |
| sex[male] | -0.7757 | 0.285 | -2.723 | 0.006 | 0.46 [0.26-0.80] |
| time_to_death | -0.0508 | 0.043 | -1.188 | 0.235 | 0.95 [0.87-1.03] |
